# Supplementary material for: Molecular basis for inner kinetochore configuration through RWD domain–peptide interactions
Source: EMBO J. 2017 Oct 18;36(23):3458–82. doi: 10.15252/embj.201796636 (PMC5709738; doi:10.15252/embj.201796636)
Supplement: Supplementary file 1 — Appendix [file EMBJ-36-3458-s001.pdf]

Appendix

Appendix Figure S1: Reconstituted *K. lactis* Nkp1-Nkp2, and sedimentation-equilibrium ultracentrifugation data analyses of *K. lactis* Ctf19-Mcm21 or *K. lactis* COMA 2

Appendix Figure S2: Subunit topology of COMA 5

Appendix Figure S3: Structured segments and flexible elements of COMA proteins in COMA-Nkp1-Nkp2, and relevance of Okp1 structured segments for *S. cerevisiae* clone-viability 8

Appendix Figure S4: Reconstituted minimized Ame1-Okp1 variants 15

Appendix Figure S5: Molecular interaction sites in Nkp1 or Nkp2 17

Appendix Figure S6: Binding sites of Okp1, Ctf19, or Mcm21 in COMA 23

Appendix Figure S7: Reconstituted Ctf19-Mcm21-Okp1 variants that bind Ctf19-Mcm21, or Ctf19-Mcm21 and Nkp1-Nkp2, and crystallographic analyses of crystals of Ctf19-Mcm21 bound with Okp1 segment 29

Appendix Figure S8: Relevance of Ctf19-Mcm21 binding motif for COMA assembly and inner kinetochore subunit-interactions 32

Appendix Figure S9: Relevance of Ctf19-Mcm21 binding motif for cell viability, chromatid segregation, or chromosome segregation 35

Appendix Figure S10: Multiple sequence alignment of Ame1 proteins from yeasts and CENP-U proteins from animals 39

Appendix supplementary material and methods 42

Appendix supplementary references 61

Appendix Figure S1: Reconstituted *K. lactis* Nkp1-Nkp2, and sedimentation-equilibrium ultracentrifugation data analyses of *K. lactis* Ctf19-Mcm21 or *K. lactis* COMA

A) Representative size-exclusion chromatography (SEC) chromatogram showing absorbance at 260 nm or 280 nm (for this chromatograph and the following ones, absorbances are in units of 1000^{-1} (mAU)), and image of Coomassie-Blue stained SDS-PAGE gel that shows fractions from principal SEC peak of recombinant *K. lactis* Nkp1-Nkp2. For this and the following SDS-PAGE gel images, molecular masses of molecular mass standards (lane denoted Mm. S.) are indicated in kilo Dalton (kDa). We stained most SDS-PAGE gels with Coomassie-Blue, unless stated otherwise. **B)** Graph with measured differential refractive index (relative scale) and molar mass calculated with multi-angle light scattering (MALS) data of *K. lactis* Nkp1-Nkp2 eluting from SEC column (SEC-MALS). Expected molar mass for Nkp1-Nkp2 heterodimer: 42822 g mol^{-1}; our experimentally determined molar mass from the principal elution peak: 39250 g mol^{-1} ± 318 g mol^{-1} (mean ± standard deviation from a single SEC-MALS experiment). **C)** Sedimentation-equilibrium ultracentrifugation data analysis of full-length *K. lactis* COMA. We show the absorbance measured at 280 nm (signal (AU)) from samples at three different concentrations, after centrifugation sequentially for 44 hrs at 8000 rpm, followed by 28 hrs at 10000 rpm, followed by 28 hrs at 15000 rpm, followed by 24 hrs at 23000 rpm. For this plot and the plot we show in **D**, radius (cm) is the distance across the sample cell that we derived absorbance measurements from for our fit. When we fit our data of COMA (with ‘global fit’) to a monomer-dimer self-association model, we calculated a molar mass of ~ 195 g mol^{-1}—between the molar mass that we expected for monomeric COMA and the molar mass for dimeric COMA. When we fixed the molar mass to monomeric COMA in our calculations, and allowed the association constant for dimerization to vary, we calculated a dissociation constant (K_d) of ~ 0.07 μM. Residuals for the plots at the bottom and those that we show in **D** are from differences of individual data points from calculated values from our data fit. **D)** Sedimentation-equilibrium ultracentrifugation data analysis of full-length *K. lactis* Ctf19-Mcm21. We show the absorbance measured at 280 nm from samples at three different concentrations, after centrifugation sequentially for 40 hrs at 9000 rpm, followed by 28 hrs at 12500 rpm, followed by 24 hrs at 15000 rpm, followed by 24 hrs at 18000 rpm, followed by 20 hrs at 22000 rpm. We used these data (in a ‘global fit’) with a ‘single species of interacting system’ model to calculate the molar mass of Ctf19-Mcm21.

Appendix Figure S1

A

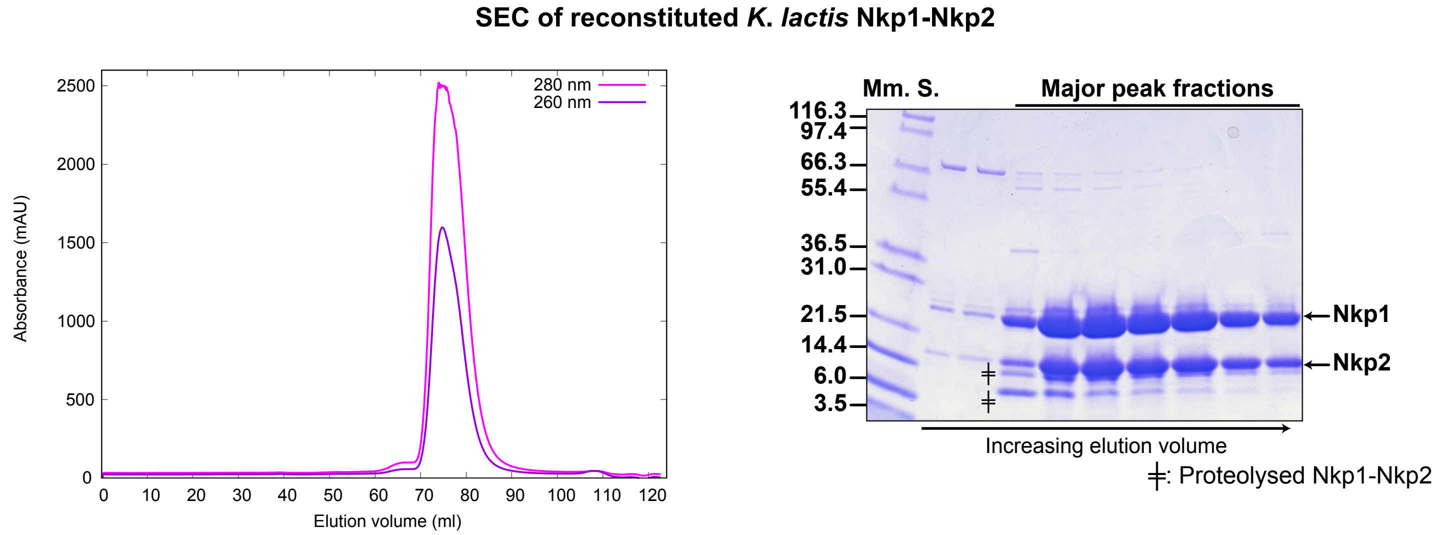

B

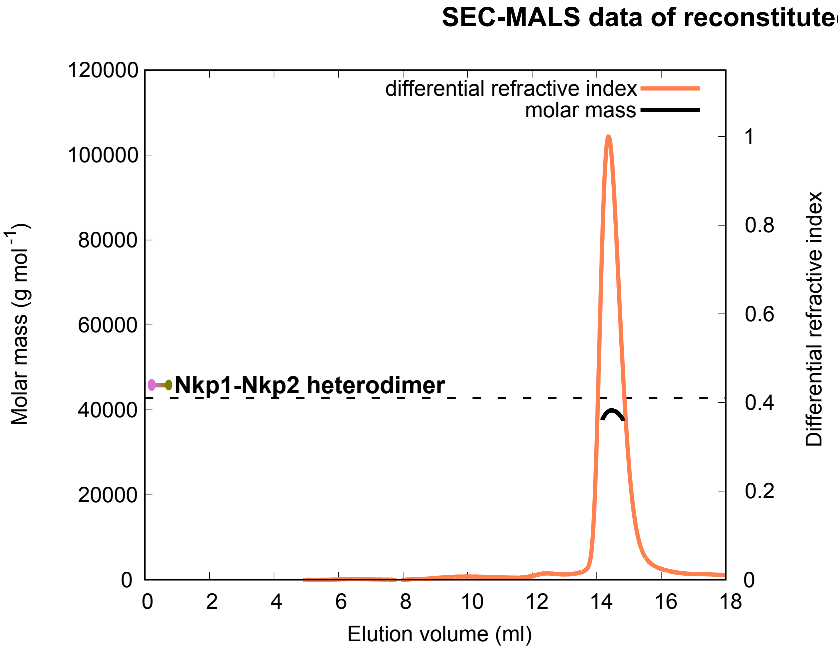

C **Sedimentation-equilibrium ultracentrifugation of *K. lactis* COMA**

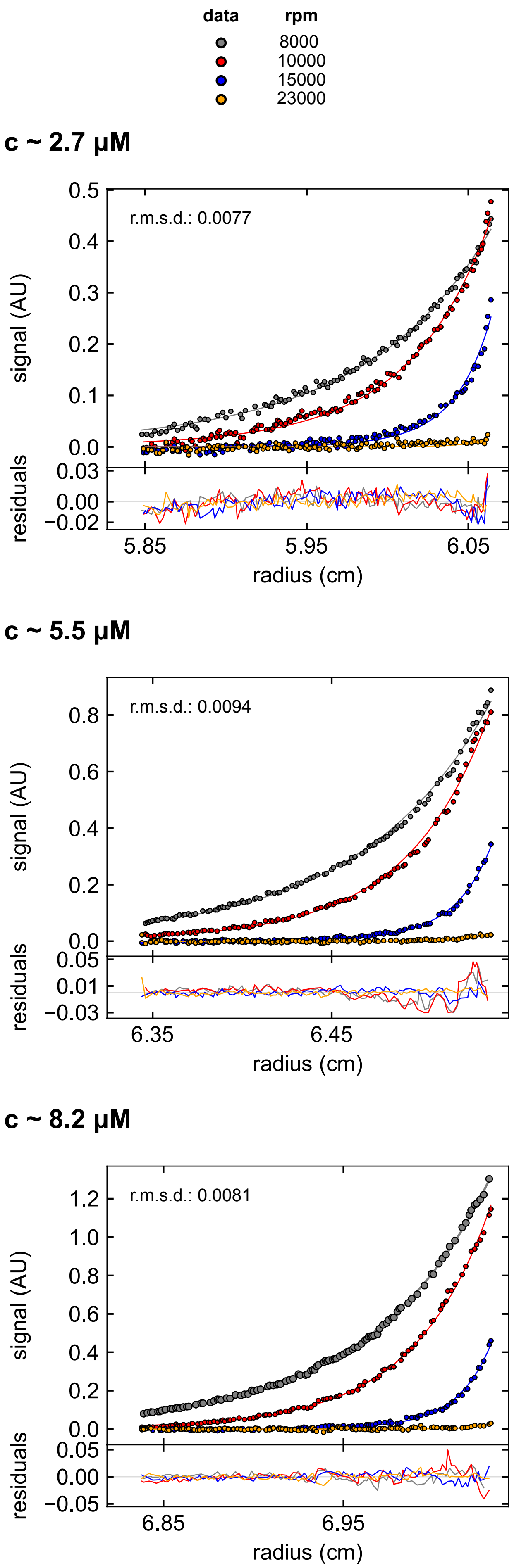

Expected molar mass of monomeric COMA: 142.3 g mol^{-1}

D **Sedimentation-equilibrium ultracentrifugation of *K. lactis* Ctf19-Mcm21**

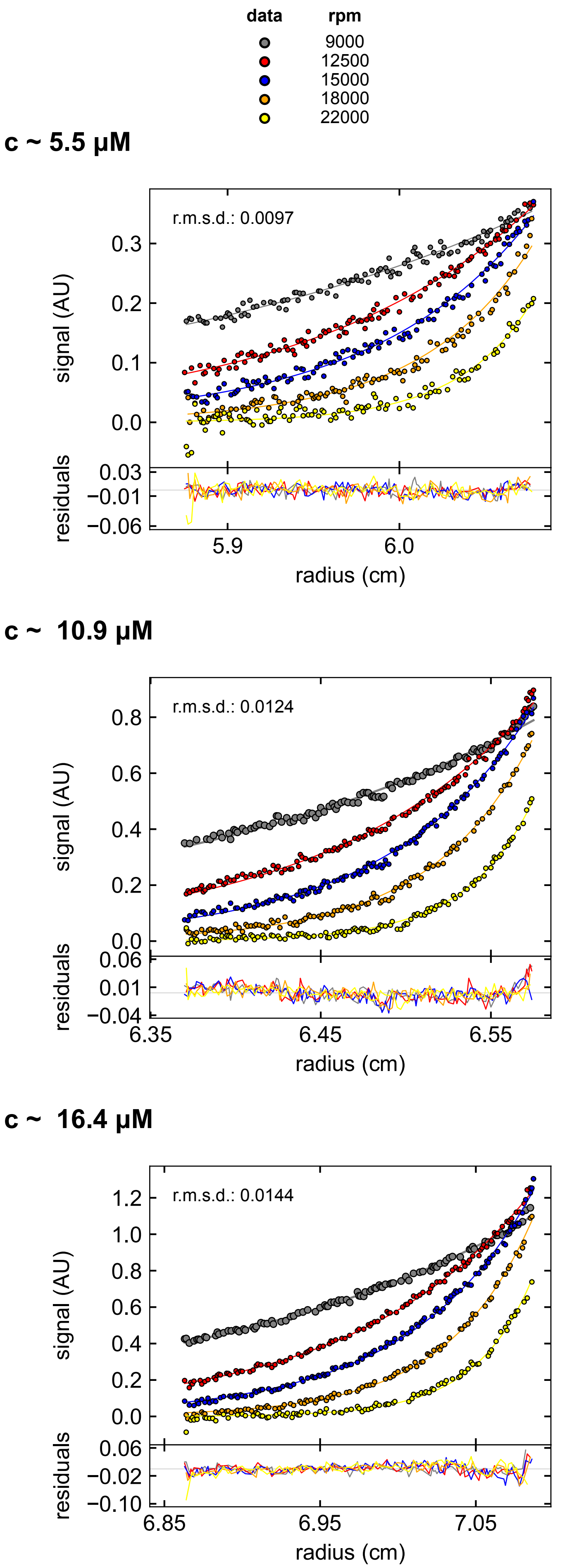

Calculated molar mass of monomeric Ctf19-Mcm21: 67.1 g mol^{-1}

Expected molar mass of monomeric Ctf19-Mcm21: 64.6 g mol^{-1}

Appendix Figure S2: Subunit topology of COMA

A) Representative nanoflow mass-spectrum of *K. lactis* COMA (bottom spectrum), which we acquired by spraying in 200 mM ammonium acetate pH 4.0. We selected the 28+ charge state of COMA for tandem mass-spectrometry (upper spectrum), before applying high collision energy in the gas phase. We observed masses corresponding to Ame1-Ctf19-Okp1, Ame1-Mcm21-Okp1, or Ctf19-Mcm21-Okp1 in the m z^{-1} range of 7000–14000. Inset mass spectra show enlarged spectrum area excerpts (from tandem mass-spectrum), which are scaled to the peak with the highest intensity in the shown area. For this spectrum and that we show in **B**, charge states of assigned masses are indicated above peaks. **Inset table** shows identified proteins or protein assemblies, their measured mass values as mean ± standard deviation from multiple peaks in a peak series assigned to the same molecular species; and the respective expected mass. Expected mass for Mcm21 includes N-terminal SNA residual. DnaK: *E. coli* DnaK chaperone that co-purified with COMA (see **Fig 1A**). **B)** Mass spectrum of COMA acquired by spraying in 100 mM ammonium acetate pH 3.7. We selected the 24+ charge state of Ctf19-Mcm21-Okp1 for tandem mass-spectrometry. Expected mass for Mcm21 includes polyhistidine tag. We attribute Mcm21 dimers in our mass spectrum to the presence of the polyhistidine tag. **C)** Plots of hydrodynamic radii (R_h; in nanometer) derived from fitting autocorrelation functions to typical dynamic light scattering data of COMA, Nkp1-Nkp2, or COMA-Nkp1-Nkp2; plotted against light scattering intensity that is shown on a relative scale. Light scattering signal-intensity corresponding to a specific peak is divided by the total signal intensity of our measurement, multiplied by 100. We derived hydrodynamic radius values that we show at the bottom from the major peak (with % polydispersity in brackets). **D)** Typical electron micrograph of negatively stained particles of COMA; scale bar: 100 nm.

Appendix Figure S2

A

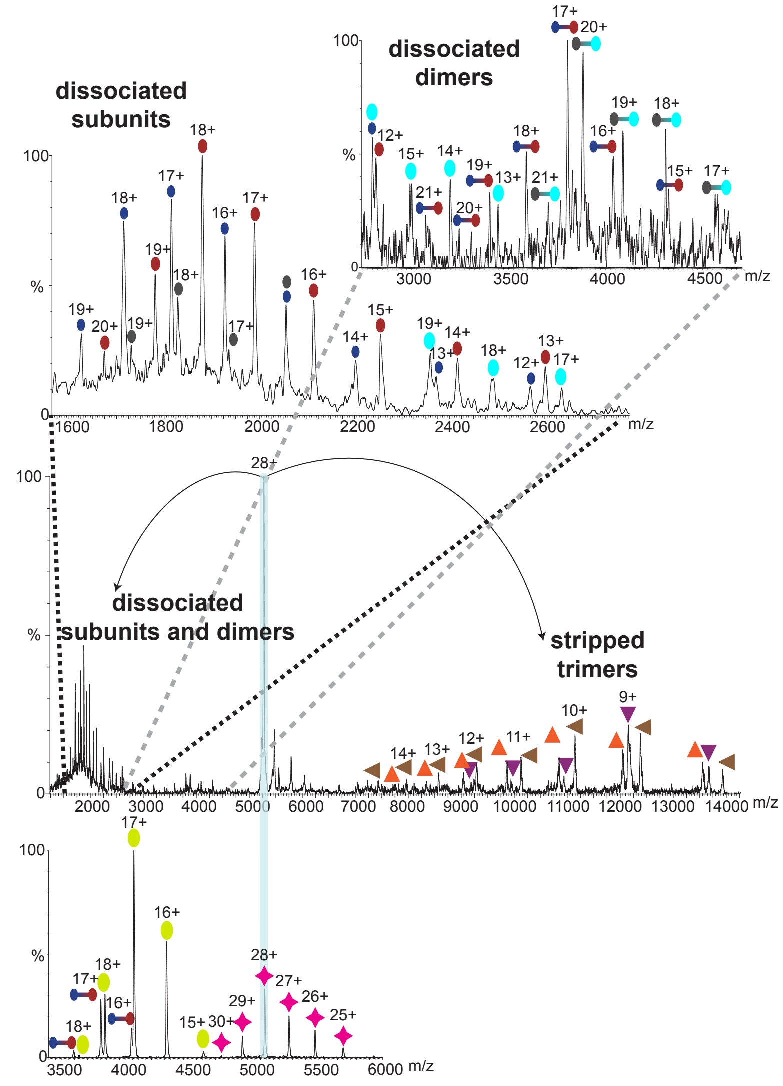

Protein or protein assembly	Expected molecular mass (Da)	Measured molecular mass (Da)
Ame1	32853	32882 ± 20
Ctf19	30805	30832 ± 14
Mcm21	33793	33793 ± 3
Okp1	44886	44845 ± 16
Ame1-Okp1	77739	77656 ± 24
Ctf19-Mcm21	64599	64640 ± 49
Ame1-Ctf19-Okp1	108544	108463 ± 66
Ame1-Mcm21-Okp1	111532	111417 ± 33
Ctf19-Mcm21-Okp1	109485	109425 ± 25
Ame1-Ctf19-Mcm21-Okp1 (COMA)	142337	142469 ± 25
DnaK (from *Escherichia coli*)	69115	69098 ± 16

B

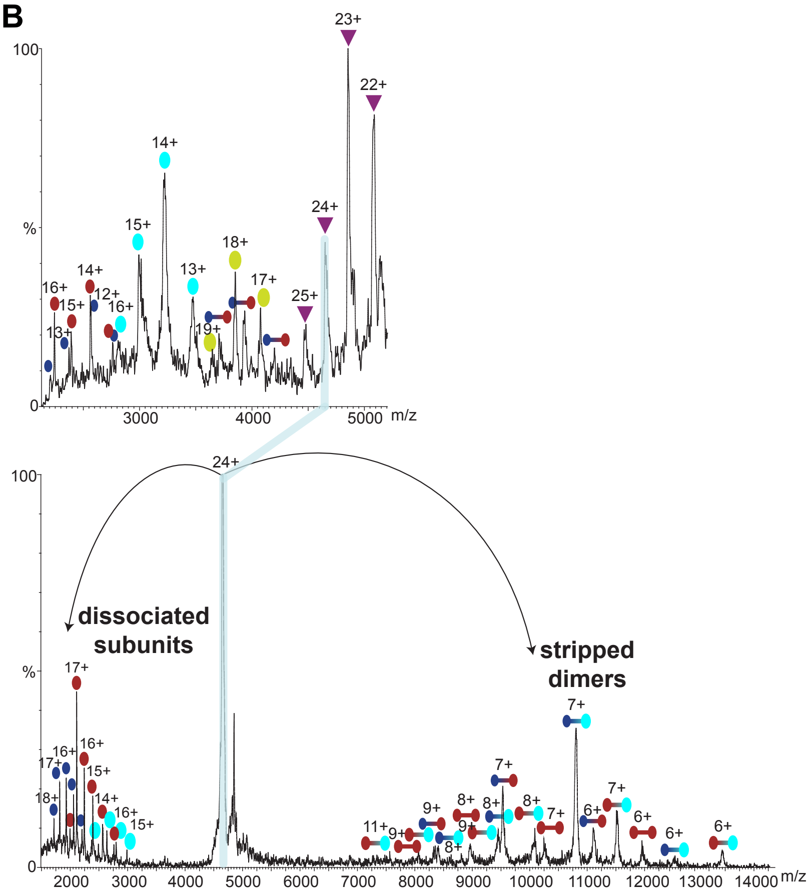

Protein or protein assembly	Expected molecular mass (Da)	Measured molecular mass (Da)
Ctf19	30805	30819 ± 21
Mcm21	35845	35854 ± 12
Okp1	44886	44791 ± 17
Ctf19-Mcm21	66650	66677 ± 53
Ctf19-Okp1	75691	75552 ± 59
Mcm21-Mcm21	71689	71718 ± 9
Mcm21-Okp1	80731	80687 ± 14
Ctf19-Mcm21-Okp1	111536	111605 ± 33
DnaK (from *Escherichia coli*)	69115	n. d.

C

Hydrodynamic radii of COMA, Nkp1-Nkp2 and COMA-Nkp1-Nkp2 from dynamic light scattering measurements

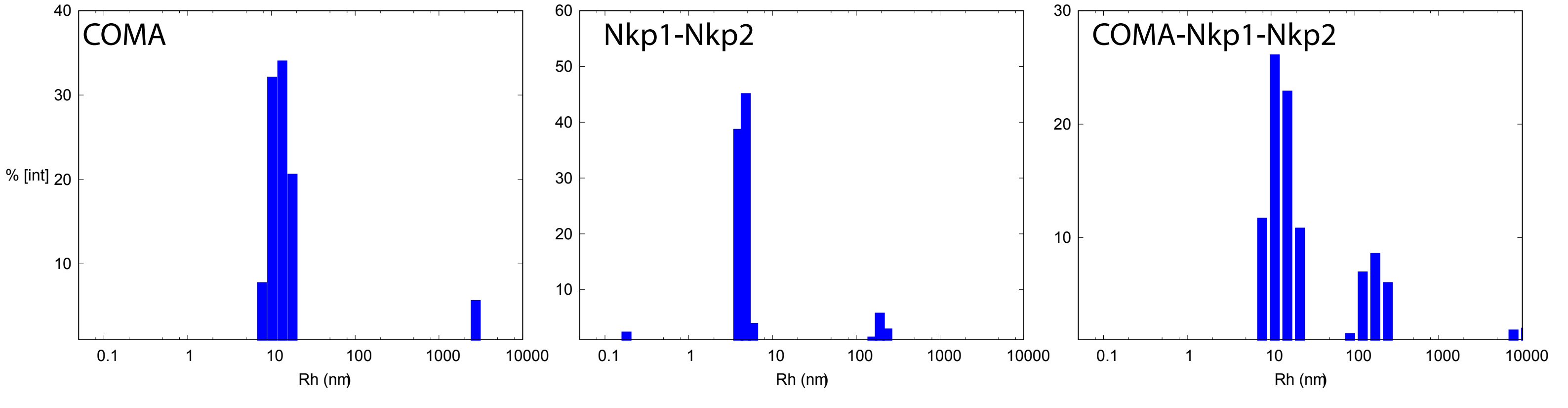

Rh(COMA): 12.9 nm (25 %)

Rh(Nkp1-Nkp2): 4.4 nm (10 %)

Rh(COMA-Nkp1-Nkp2): 13.7 nm (33 %)

D

Electron micrograph of negatively stained COMA particles

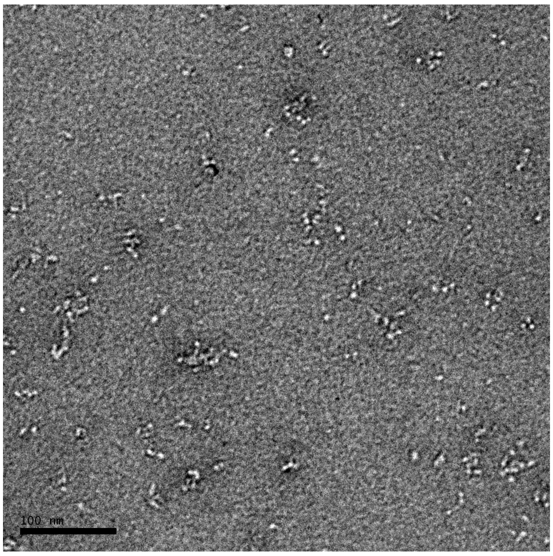

Appendix Figure S3: Structured segments and flexible elements of COMA proteins in COMA-Nkp1-Nkp2, and relevance of Okp1 structured segments for *S. cerevisiae* clone-viability

A,B,C) Representative plots showing deuterium-exchanged peptides, after 10 sec, 60 sec, or 1200 sec of deuterium exchange of Ctf19 (**A**), Mcm21 (**B**), or Okp1 (**C**), each in COMA or in COMA-Nkp1-Nkp2. General formatting is as for **Fig 3**. The globular D-RWD domains of Ctf19 or Mcm21 are indicated with thicker lines on top of the plot. Mcm21 that we used for our experiments had an N-terminal SNA residual—residue numbering starts with the native starting methionine. Formatting for Okp1-sequence representation is as for **Fig 3A**. **D)** Representative images of spores from our tetrad dissection of heterozygous diploid *S. cerevisiae* clones with an *Okp1* version that lacks the coding region (see **Fig EV5**) for core (*Okp1*/*Okp1_coreΔ*)—Okp1 residues 162–189, or segment 2 (*Okp1*/*Okp1_segment2Δ*)—Okp1 residues 236–265, after two days of growth on solid YPD agar at 30 °C. Bottom: representative images of spores from our tetrad dissection of heterozygous diploid *S. cerevisiae* clones with an integrated version of full-length *Okp1 (Okp1_fl)*, or *Okp1* versions that lack the coding region for Okp1 segment 1 (*Okp1*/*Okp1_segment1Δ*)—Okp1 residues 325–337, or segment 3 (*Okp1/Okp1_segment3Δ*)—Okp1 residues 353–400, after growth at 25 °C or 30 °C on solid YPD agar. **E)** Representative plots showing deuterium-exchanged peptides, after 10 sec, 60 sec, or 1200 sec of deuterium exchange of Ame1 in COMA or COMA-Nkp1-Nkp2. Formatting is as for **Fig 3B**. **F)** Multiple sequence alignment of Ame1 orthologues from budding yeasts *Kluyveromyces lactis* (Refseq accession-code: XP_452444.1), *Saccharomyces cerevisiae* (NP_009770.3), *Vanderwaltozyma polyspora* (XP_001643640.1), *Zygosaccharomyces rouxii* (XP_002495879.1), *Eremothecium gossypii* (NP_982693.1), *Lachancea thermotolerans* (XP_002556178.1), *Candida glabrata* (GenBank accession code: KTB16991.1). General formatting is analogous to that for alignment that we show in **Fig EV5**. Structured segments and predicted coiled coil are as in **Fig 3B**. Positions of proteolysis sites from fragments from our limited proteolysis experiments (see **Tables EV1**;**2**) are indicated with vertical arrows. The MIND binding site that was previously identified (Hornung et al, 2014) is highlighted too.

Appendix Figure S3

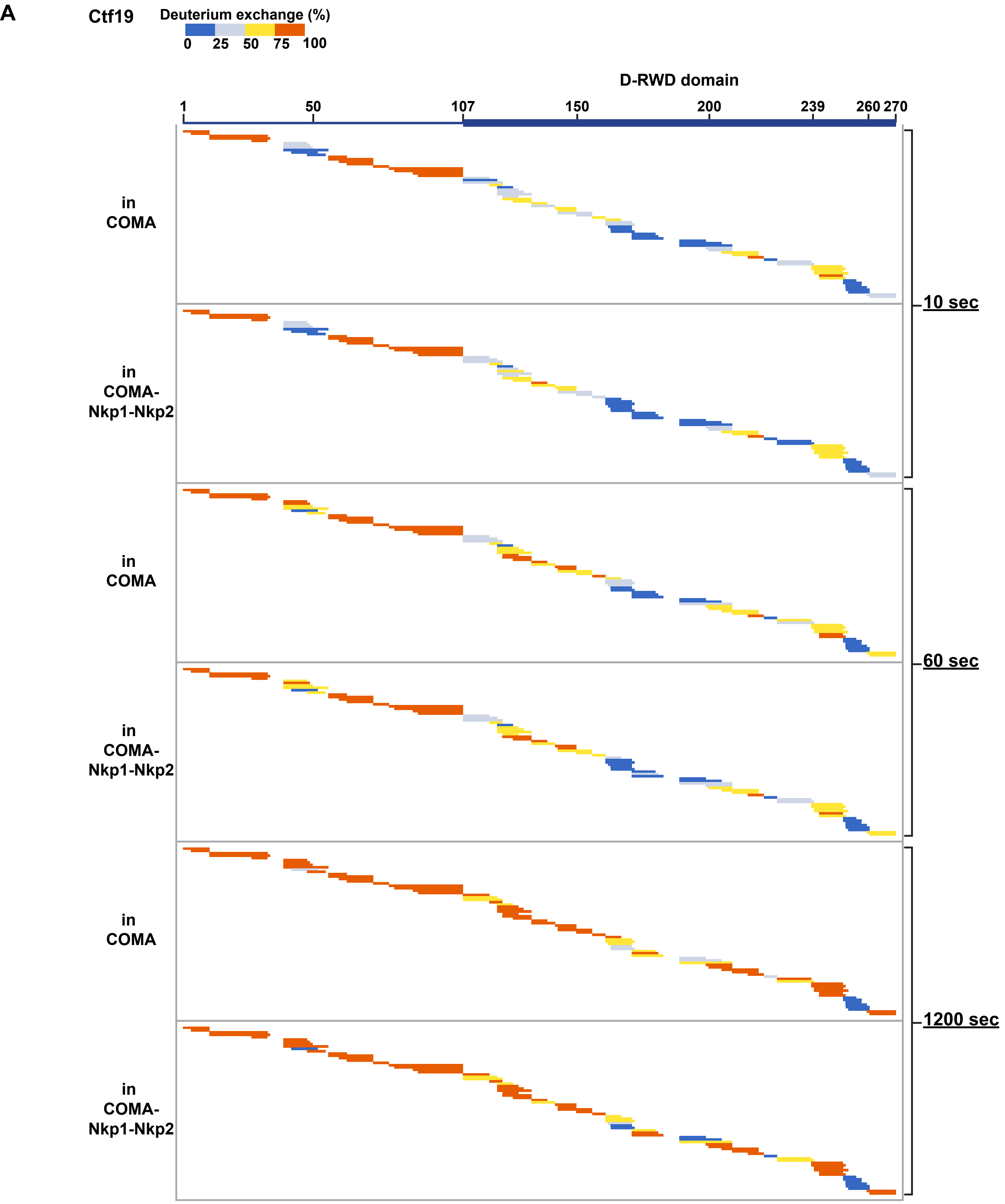

B

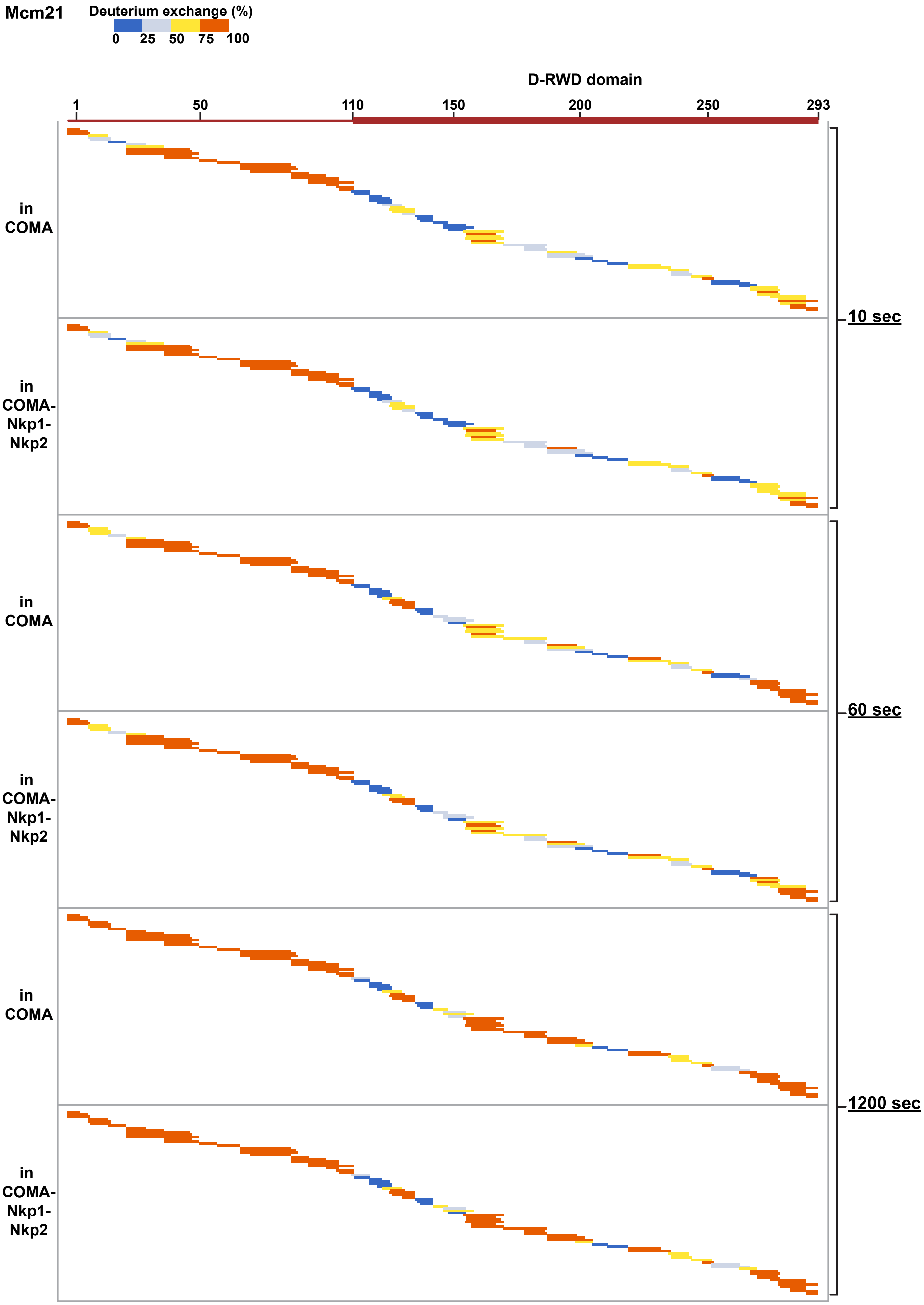

Mcm21
Deuterium exchange (%)
0
25
50
75
100
D-RWD domain
1
50
110
150
200
250
293
in COMA
in COMA-Nkp1-Nkp2
in COMA
in COMA-Nkp1-Nkp2
in COMA
in COMA-Nkp1-Nkp2
10 sec
60 sec
1200 sec

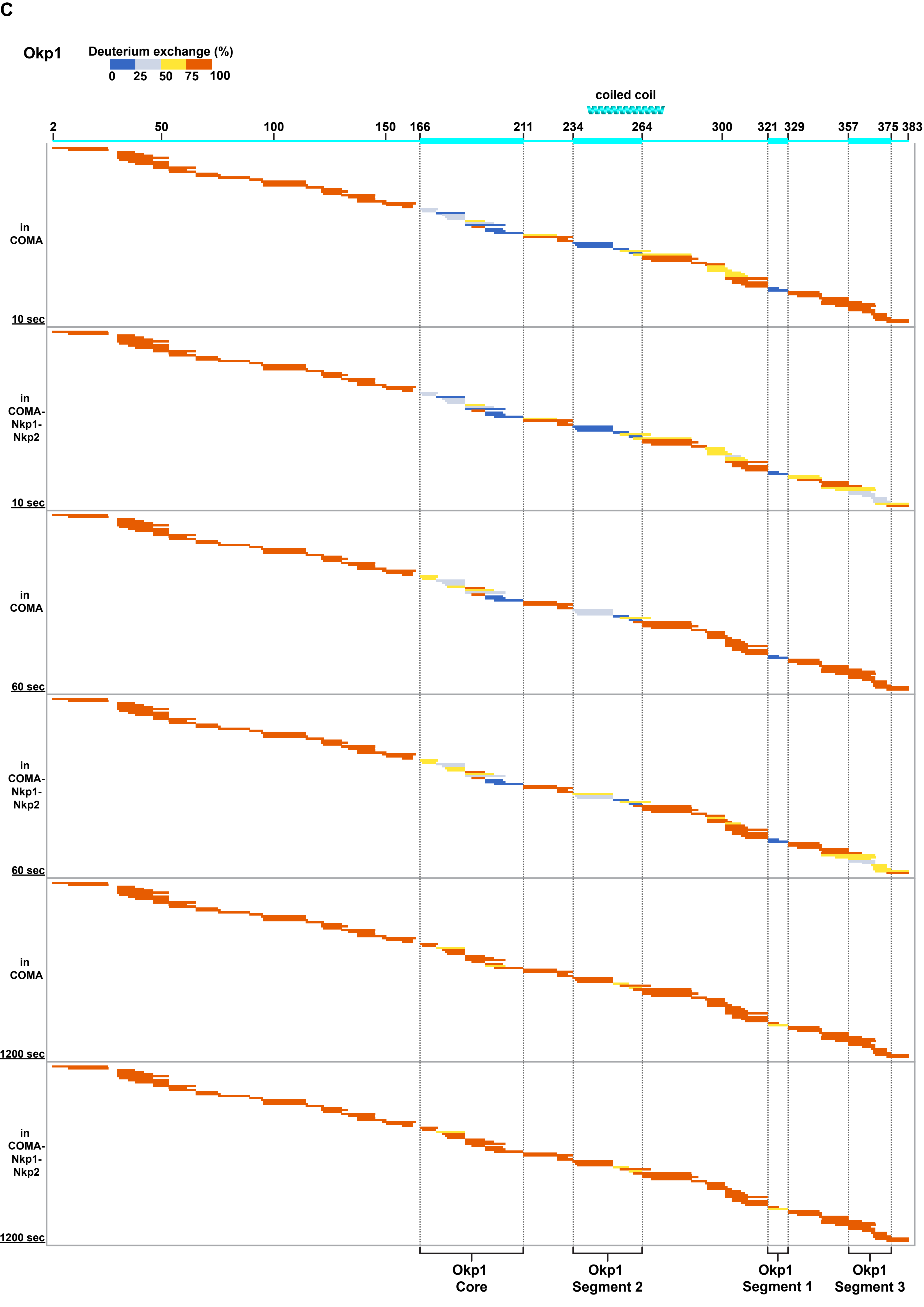
C
Okp1
Deuterium exchange (%)
0 25 50 75 100
coiled coil
2 50 100 150 166 211 234 264 300 321 329 357 375 383
in COMA
10 sec
in COMA-Nkp1-Nkp2
10 sec
in COMA
60 sec
in COMA-Nkp1-Nkp2
60 sec
in COMA
1200 sec
in COMA-Nkp1-Nkp2
1200 sec
Okp1 Core
Okp1 Segment 2
Okp1 Segment 1
Okp1 Segment 3

D

Spores from dissected tetrads of heterozygous diploid *S. cerevisiae* clones with different Okp1 versions

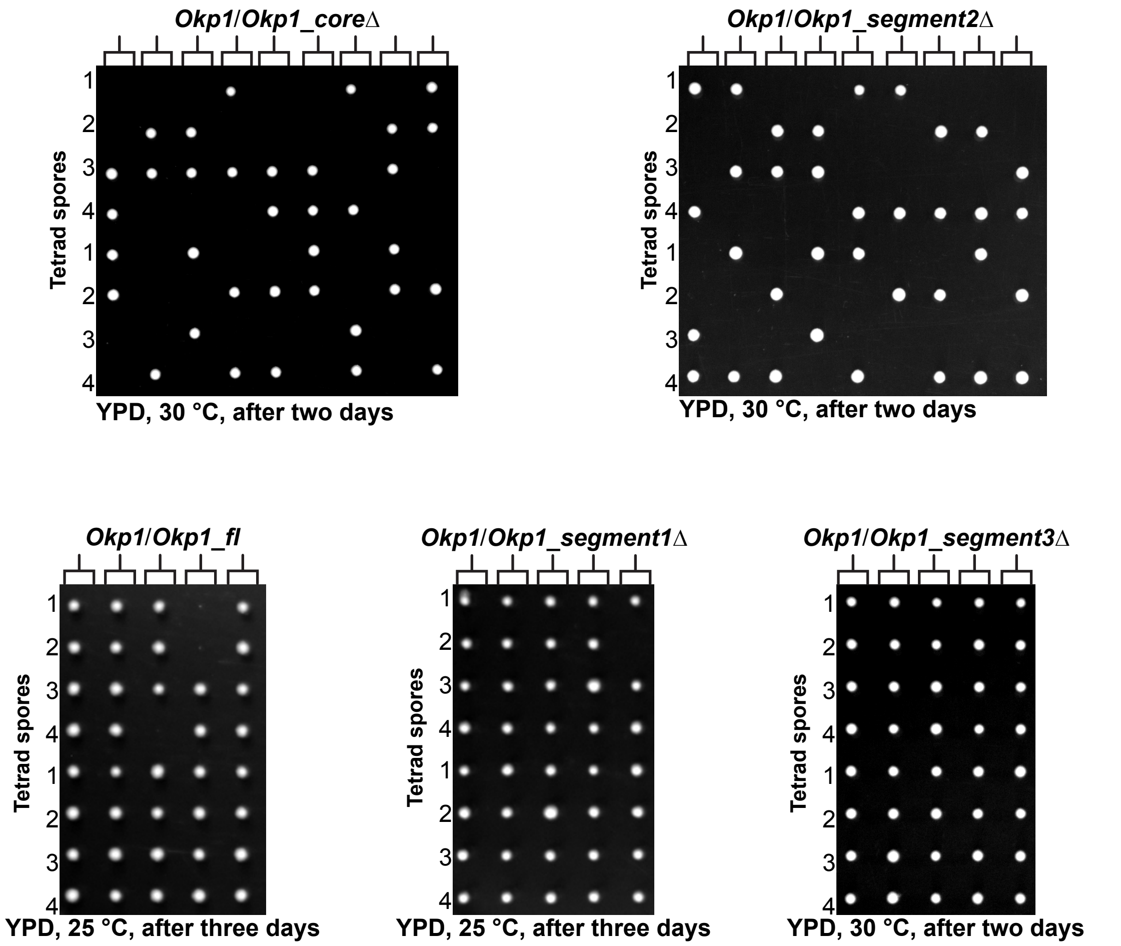

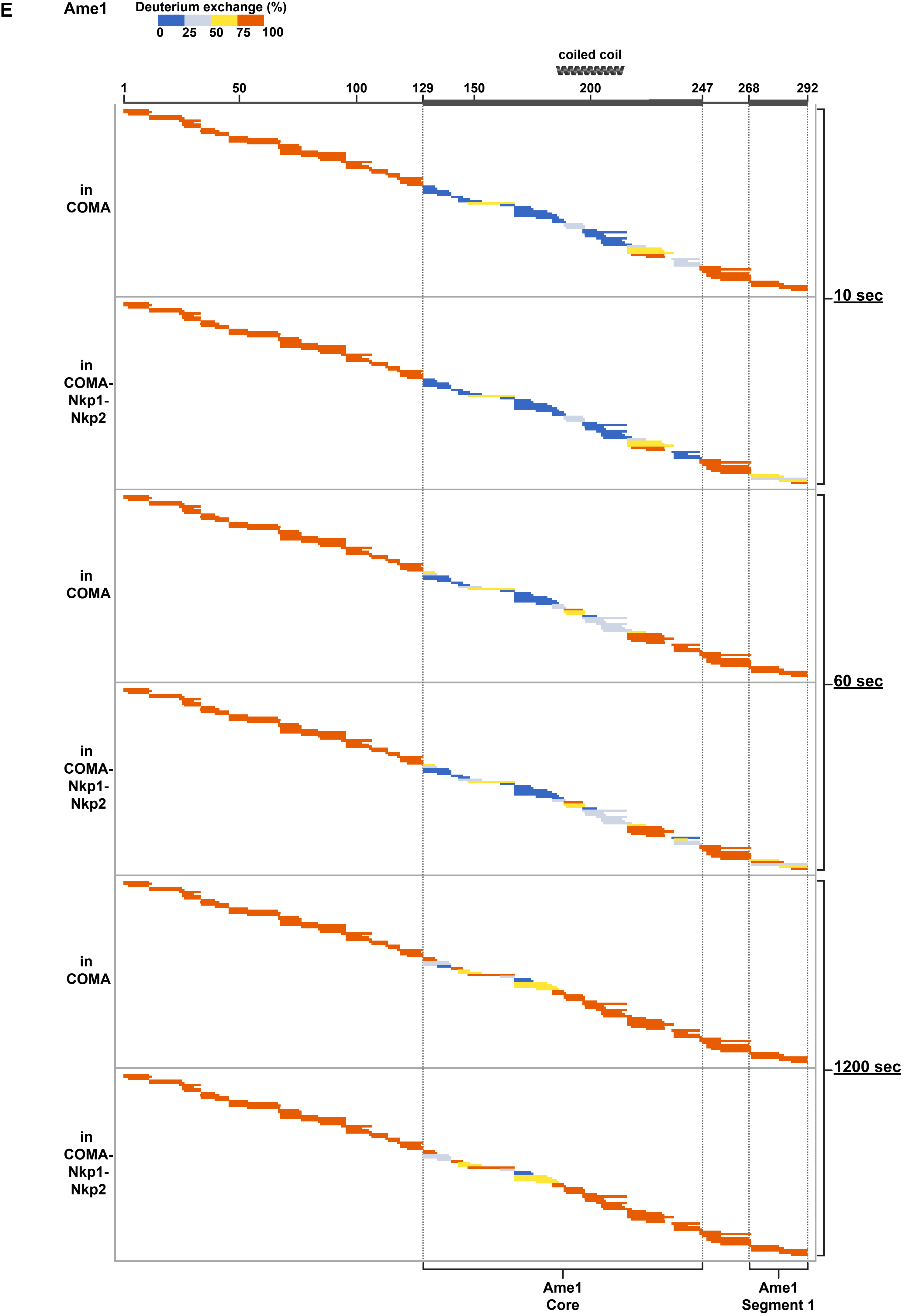

E
Ame1
Deuterium exchange (%)
0
25
50
75
100
coiled coil
1
50
100
129
150
200
247
268
292
in
COMA
in
COMA-
Nkp1-
Nkp2
10 sec
60 sec
1200 sec
Ame1
Core
Ame1
Segment 1

F

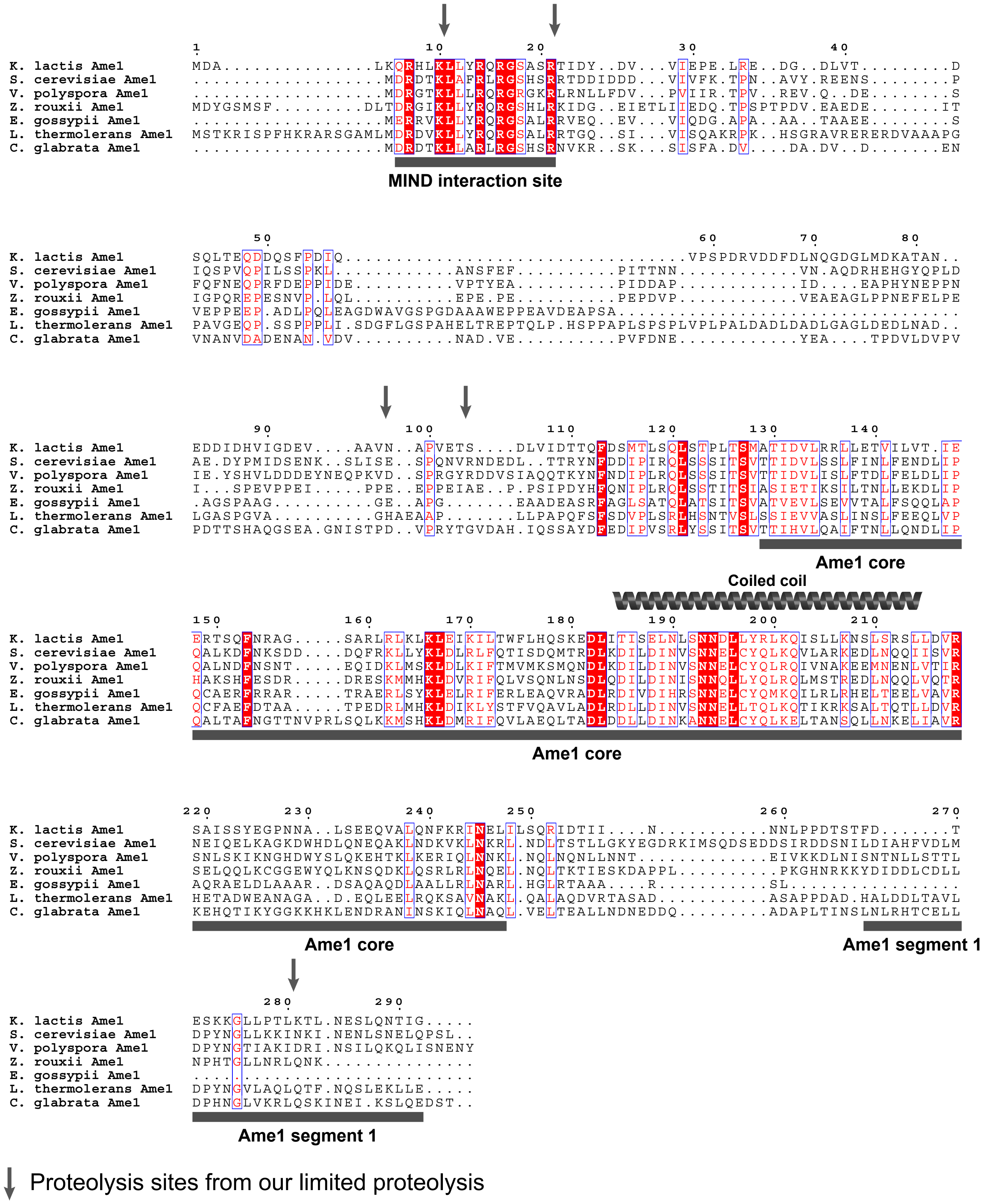

K. lactis Ame1
S. cerevisiae Ame1
V. polyspora Ame1
Z. rouxii Ame1
E. gossypii Ame1
L. thermolerans Ame1
C. glabrata Ame1
MIND interaction site
Ame1 core
Coiled coil
Ame1 core
Ame1 core
Ame1 segment 1
Ame1 segment 1
Proteolysis sites from our limited proteolysis

Appendix Figure S4: Reconstituted minimized Ame1-Okp1 variants
A,B) Representative SEC chromatograms showing absorbance at 260 nm and 280 nm, and images of SDS-PAGE gel with fractions from principal SEC peak from purification of two of our recombinant *K. lactis* Ame1-Okp1 variants with truncated Ame1 (Ame1 residues 98–225: Ame1_98–225 or Ame1 residues 1–260: Ame1_1–260) and Okp1 that is N-terminally and C-terminally truncated (Okp1 residues 123–308: Okp1_123–308 or Okp1 residues 106–308: Okp1_106–308). The SEC elution-volume range of principal peak fractions that we analysed on SDS-PAGE is indicated below the chromatogram.

We could not obtain substantial amounts of soluble full-length *K. lactis* Ame1-Okp1 from expression of an *Okp1-Ame1* dicistron.

Appendix Figure S4

SEC of reconstituted *K. lactis* Ame1-Okp1 variants

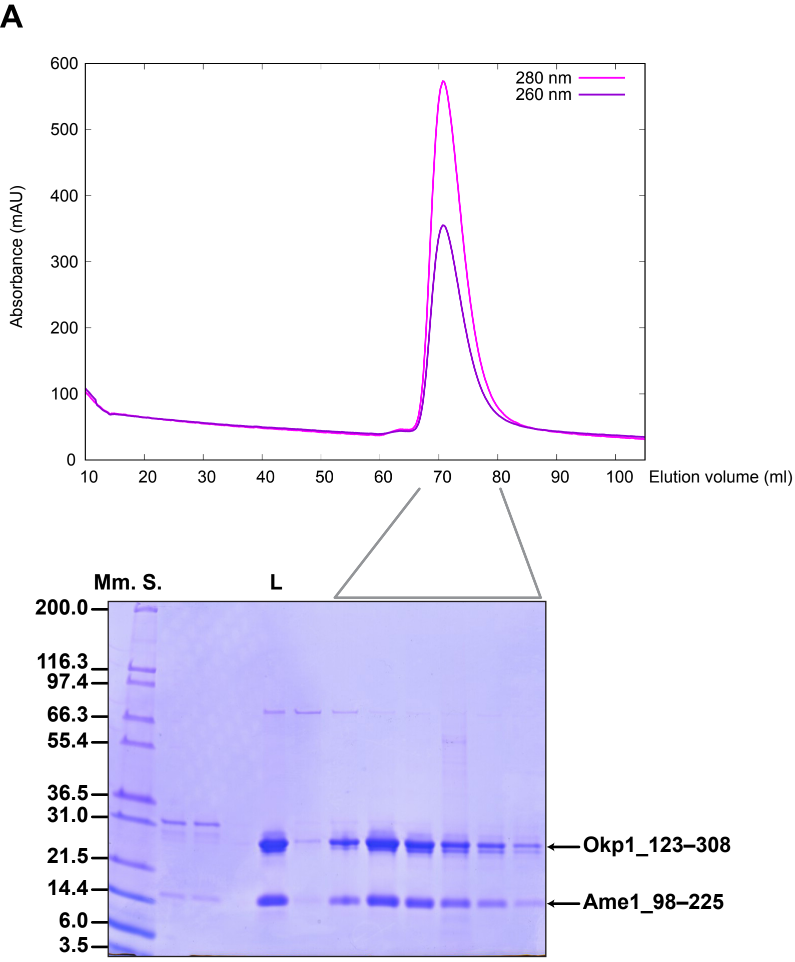

Mm. S.: Molecular mass standards
L: loaded on size-exclusion chromatography column

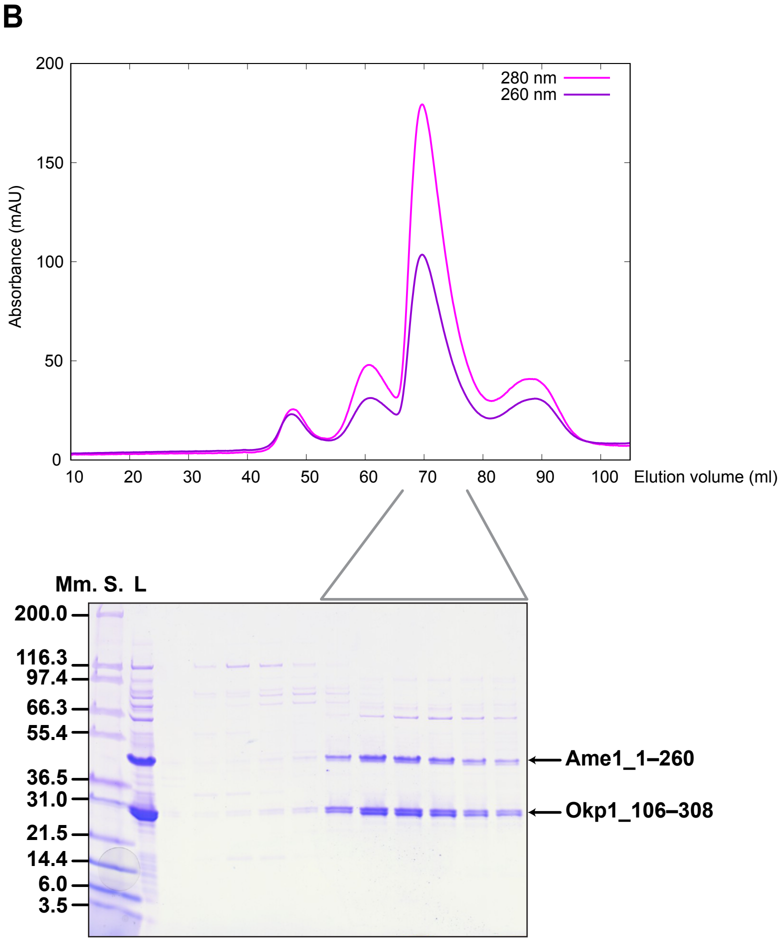

Mm. S.: Molecular mass standards
L: loaded on size-exclusion chromatography column

Appendix Figure S5: Molecular interaction sites in Nkp1 or Nkp2

A, B) Representative plots showing deuterium-exchanged peptides, after 10 sec, 60 sec, or 1200 sec of deuterium exchange of *K. lactis* Nkp1 (**A**) or *K. lactis* Nkp2 (**B**), each in Nkp1-Nkp2 or in COMA-Nkp1-Nkp2. Nkp1 has an N-terminal SNA residual; numbering starts with the native methionine. General formatting is as we describe for **Fig 3**. **C)** Multiple sequence alignment of Nkp1 orthologues from budding yeasts *Kluyveromyces lactis* (Refseq data base accession-code: XP_454075.1), *Saccharomyces cerevisiae* (NP_010671.4), *Vanderwaltozyma polyspora* (XP_001646030.1), *Zygosaccharomyces rouxii* (XP_002495060.1), *Lachancea thermotolerans* (XP_002554852.1), *Candida glabrata* (XP_444906.1). General formatting for alignment is as for **Fig EV5**. Proteolysis sites from our limited proteolysis experiments (**Tables EV1**;**2**;**3**) we show as vertical arrows. **D)** Multiple sequence alignment of Nkp2 orthologues from budding yeasts *Kluyveromyces lactis* (Refseq data base accession-code: XP_453021.1), *Saccharomyces cerevisiae* (NP_013419.1), *Vanderwaltozyma polyspora* (XP_001645198.1), *Zygosaccharomyces rouxii* (XP_002498459.1), *Lachancea lanzarotensis* (GenBank data base accession code: CEP61777.1), *Candida glabrata* (GenBank data base accession code: KTA95770.1). Through analyses of our mass spectra of samples from our limited proteolysis of *K. lactis* COMA-Nkp1-Nkp2, we found Nkp1 and Nkp2 to be the least protease-sensitive proteins in COMA-Nkp1-Nkp2 (**Tables EV1**;**2**; **Fig EV4**). With our limited proteolysis experiments, we found that Nkp1-Nkp2 is especially protease-sensitive in the C-terminal part of Nkp1 and Nkp2, in the vicinity of residue 150 in Nkp1, and residue 116 in Nkp2 (**Table EV3**). Residues around these sites of Nkp1 and Nkp2–possibly in projecting loops–are probable contacts for Okp1-Ame1 C-termini, because the regions surrounding these sites are stabilized in COMA-Nkp1-Nkp2 (see **A** or **B**). Nkp1-Nkp2 spontaneously proteolysed in Nkp1-Nkp2; but was less prone to proteolysis when associated with COMA/Okp1-Ame1 (see **Fig 1A**; **Appendix Fig S1A**; **Table EV1**). With mass spectrometry, we found the following fragments of Nkp1 and Nkp2—the result of spontaneous proteolysis—in our purified Nkp1-Nkp2 sample: Nkp1 residues 1–60; and lower amounts of Nkp1 fragments with residues 1–57, 25–174 or 25–175; for Nkp2: 1–68, and a lower amount of an Nkp2 fragment with residues 1–67. **E)** Representative plots showing deuterium-exchanged peptides, after 10 sec, 60 sec, or 1200 sec of deuterium exchange of Nkp1 alone or Nkp1 in Nkp1-Nkp2. Nkp1 residues 1-60 are flexible in Nkp1, but stabilized in Nkp1-Nkp2. Our observations suggest that the Nkp1 N terminus contacts Nkp2. The Nkp1 C-terminal part, which becomes structured upon binding of Nkp1-Nkp2 to COMA, is partially ordered in Nkp1 alone, but is not ordered in Nkp1-Nkp2, suggesting conformational changes in Nkp1 upon binding to Nkp2. **F)** Representative SEC chromatogram, and image of SDS-PAGE gel with fractions from principal SEC peak, of an Nkp1-Nkp2 variant with N-terminally truncated Nkp1 (residues 13–210), and C-terminally truncated Nkp2 (residues 1–143); L: sample loaded on column. The N-terminal 12 residues of Nkp1 and C-terminal 8 residues in Nkp2 are dispensable for Nkp1-Nkp2 formation. But we found that Nkp2 (without C terminal 19 residues) did not co-purify with polyhistidine-tagged Nkp1 without its N terminal 30 residues (data not shown), when we co-expressed coding regions for these variants; consistent with our suggestion (see **E**) that the Nkp1 N terminus contacts Nkp2.

Appendix Figure S5

A

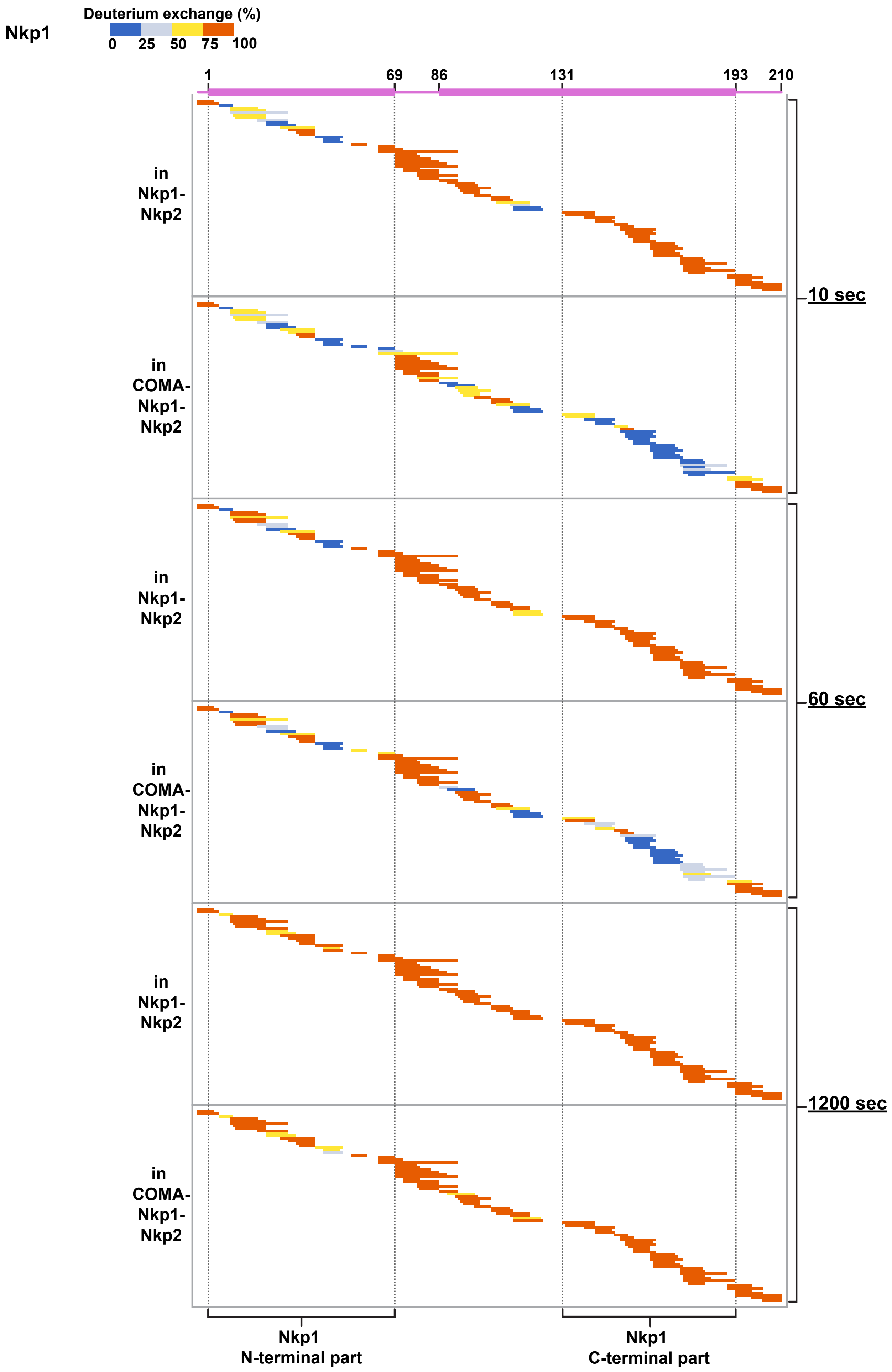

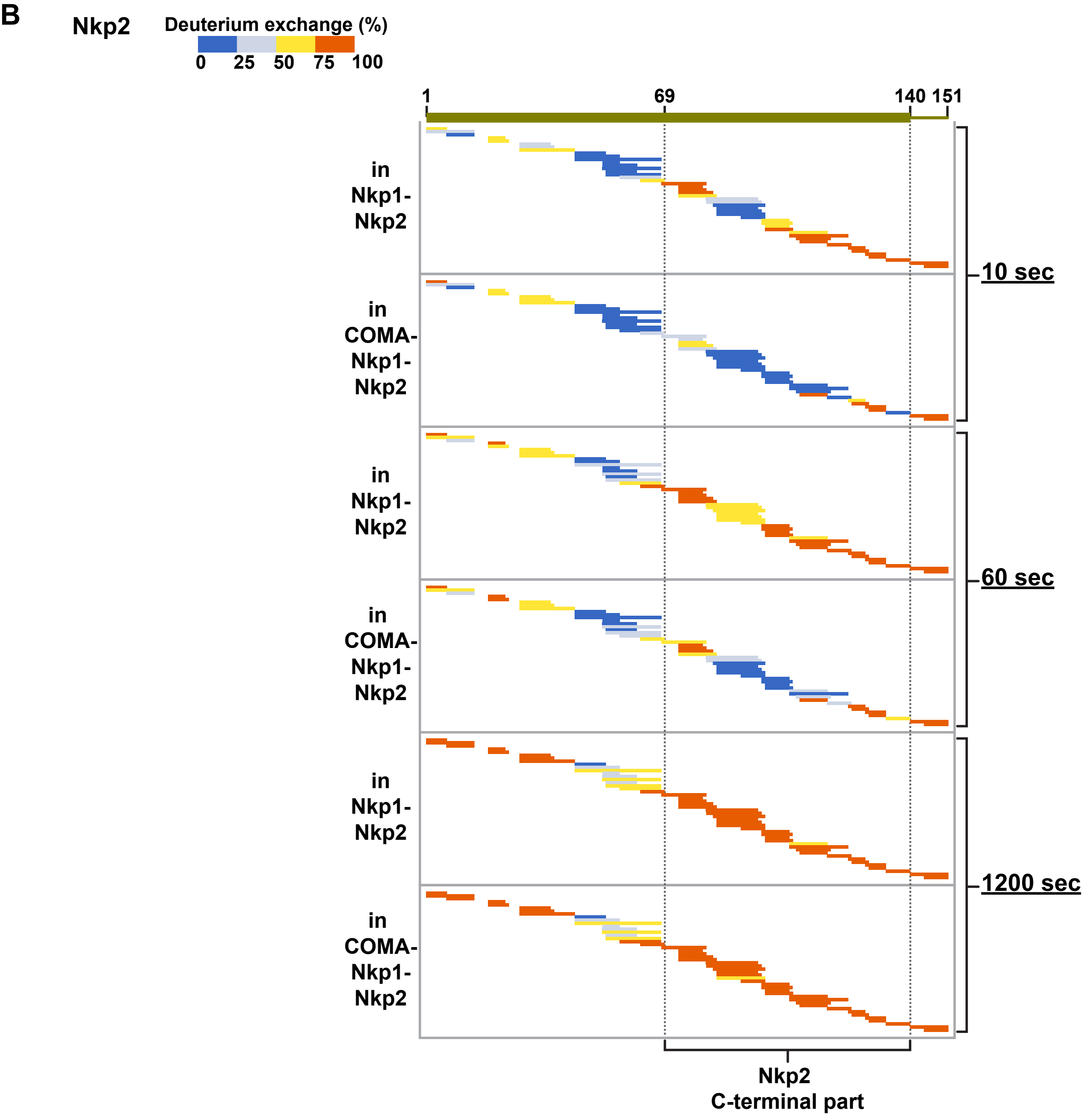
B
Nkp2
Deuterium exchange (%)
0
25
50
75
100
1
69
140
151
in Nkp1-Nkp2
in COMA-Nkp1-Nkp2
in Nkp1-Nkp2
in COMA-Nkp1-Nkp2
in Nkp1-Nkp2
in COMA-Nkp1-Nkp2
10 sec
60 sec
1200 sec
Nkp2 C-terminal part

C

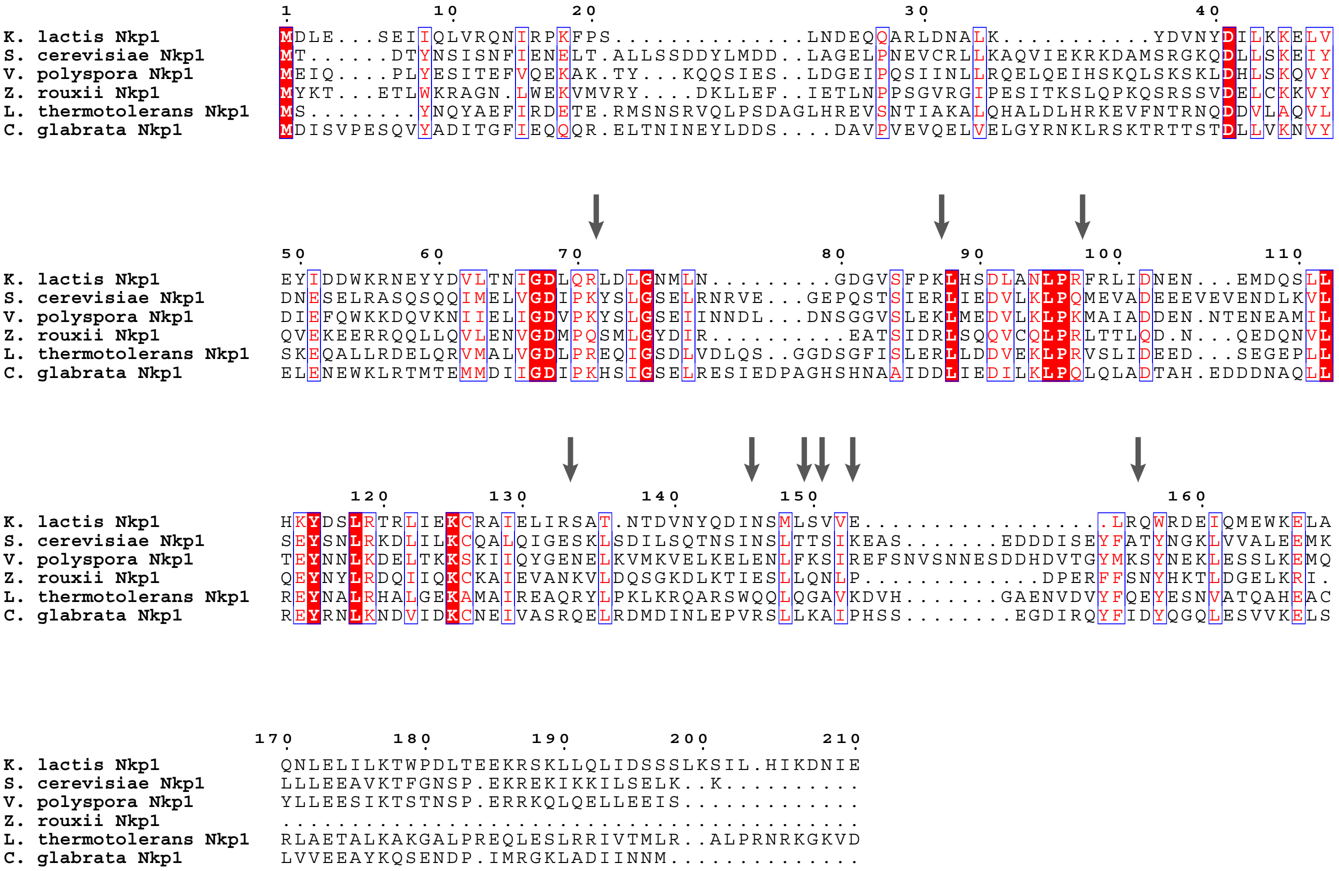

1 10 20 30 40
K. lactis Nkp1 MDLE...SEIIQLVRQNIRPKFPS..............LNDEQQARLDNALK...........YDVNYDILKKELV
S. cerevisiae Nkp1 MT......DTYNSISNFIENELT.ALLSSDDYLMDD..LAGELPNEVCRLLKAQVIEKRKDAMSRGKQDLLSKEIY
V. polyspora Nkp1 MEIQ....PLYESITEFVQEKAK.TY...KQQSIES..LDGEIPQSIINLLRQELQEIHSKQLSKSKLDHLSKQVY
Z. rouxii Nkp1 MYKT...ETLWKRAGN.LWEKVMVRY....DKLLEF..IETLNPPSGVRGIPESITKSLQPKQSRSSVDELCKKVY
L. thermotolerans Nkp1 MS........YNQYAEFIRDETE.RMSNSRVQLPSDAGLHREVSNTIAKALQHALDLHRKEVFNTRNQDDVLAQVL
C. glabrata Nkp1 MDISVPESQVYADITGFIEQQQR.ELTNINEYLDDS....DAVPVEVQELVELGYRNKLRSKTRTTSTDLLVKNVY
50 60 70 80 90 100 110
K. lactis Nkp1 EYIDDWKRNEYYDVLTNIGDLQRLDLGNMLN.........GDGVSFPKLHSDLANLPRFRLIDNEN...EMDQSLL
S. cerevisiae Nkp1 DNESELRASQSQQIMELVGDIPKYSLGSELRNRVE.....GEPQSTSIERLIEDVLKLPQMEVADEEEVEVENDLKVL
V. polyspora Nkp1 DIEFQWKKDQVKNIIELIGDVPKYSLGSEIINNDL...DNSGGVSLEKLMEDVLKLPKMAIADDEN.NTENEAMIL
Z. rouxii Nkp1 QVEKEERRQQLLQVLENVGDMPQSMLGYDIR..........EATSIDRLSQQVCQLPRLTTLQD.N...QEDQNVL
L. thermotolerans Nkp1 SKEQALLRDELQRVMALVGDLPREQIGSDLVDLQS..GGDSGFISLERLLDDVEKLPRVSLIDEED...SEGEPLL
C. glabrata Nkp1 ELENEWKLRTMTEMMDIIGDIPKHSIGSELRESIEDPAGHSHNAAIDDLIEDILKLPQLQLADTAH.EDDDNAQLL
120 130 140 150 160
K. lactis Nkp1 HKYDSLRTRLIEKCRAIELIRSAT.NTDVNYQDINSMLSVVE.................LRQWRDEIQMEWKELA
S. cerevisiae Nkp1 SEYSNLRKDLILKCQALQIGESKLSDILSQTNSINSLTTSIKEAS.......EDDDISEYFATYNGKLVVALEEMK
V. polyspora Nkp1 TEYNNLKDELTKKSKIIQYGENELKVMKVELKELENLFKSIREFSNVSNNESDDHDVTGYMKSYNEKLESSLKEMQ
Z. rouxii Nkp1 QEYNYLRDQIIQKCKAIEVANKVLDQSGKDLKTIESLLQNLP.............DPERFFSNYHKTLDGELKRI.
L. thermotolerans Nkp1 REYNALRHALGEKAMAIREAQRYLPKLKRQARSWQQLQGAVKDVH.......GAENVDVYFQEYESNVATQAHEAC
C. glabrata Nkp1 REYRNLKNDVIDKCNEIVASRQELRDMDINLEPVRSLLKAIPHSS........EGDIRQYFIDYQGQLESVVKELS
170 180 190 200 210
K. lactis Nkp1 QNLELILKTWPDLTEEKRSKLLQLIDSSSLKSIL.HIKDNIE
S. cerevisiae Nkp1 LLLEEAVKTFGNSP.EKREKIKKILSELK..K..........
V. polyspora Nkp1 YLLEESIKTSTNSP.ERRKQLQELLEEIS.............
Z. rouxii Nkp1 ....
L. thermotolerans Nkp1 RLAETALKAKGALPREQLESLRRIVTMLR..ALPRNRKGKVD
C. glabrata Nkp1 LVVEEAYKQSENDP.IMRGKLADIINNM..............

D

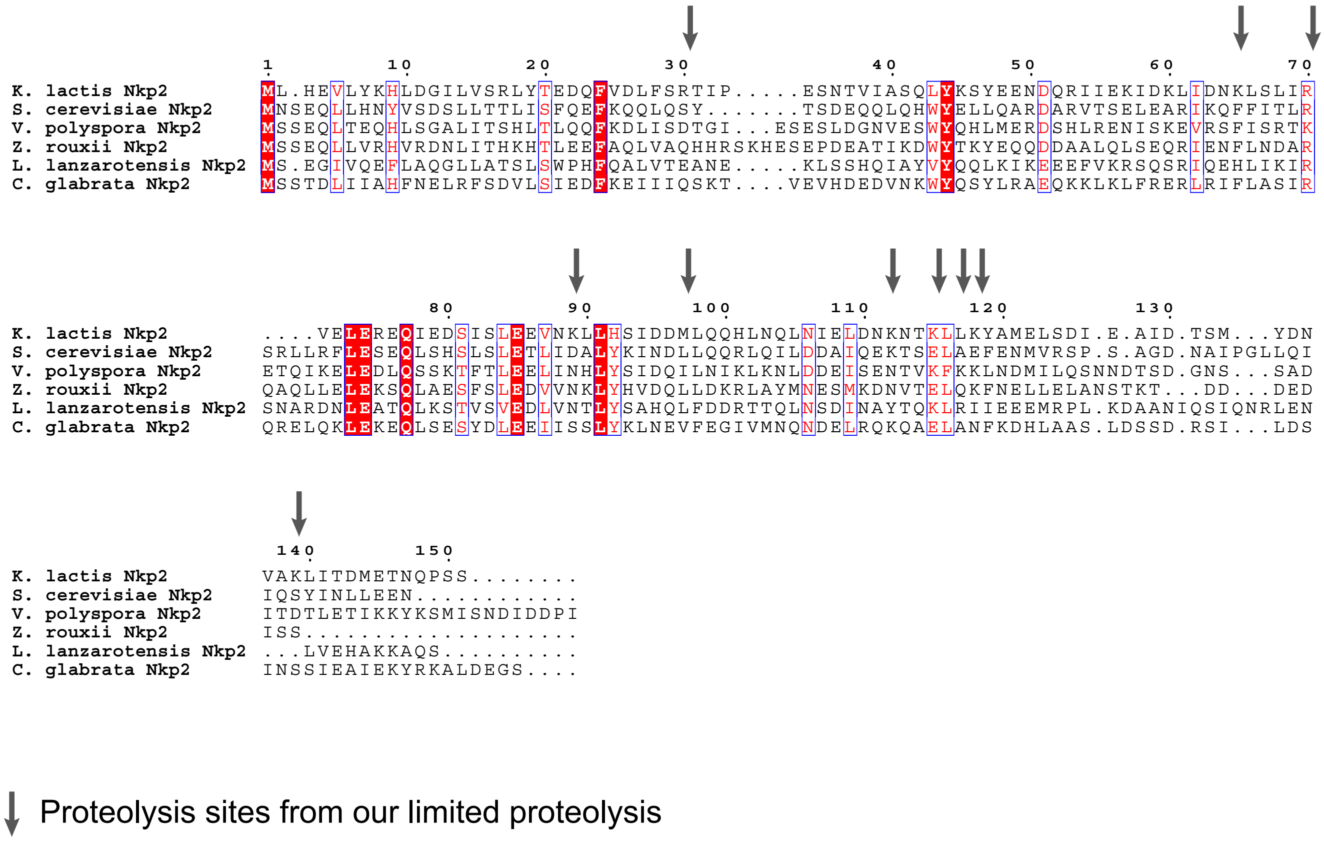

1 10 20 30 40 50 60 70
K. lactis Nkp2 ML.HEVLYKHLDGILVSRLYTEDQFVDLFSRTIP.....ESNTVIASQLYKSYEENDQRIIEKIDKLIDNKLSLIR
S. cerevisiae Nkp2 MNSEQLLHNYVSDSLLTTLISFQEFKQQLQSY.......TSDEQQLQHWYELLQARDARVTSELEARIKQFFITLR
V. polyspora Nkp2 MSSEQLTEQHLSGALITSHLTLQQFKDLISDTGI...ESESLDGNVESWYQHLMERDSHLRENISKEVRSFISRTK
Z. rouxii Nkp2 MSSEQLLVRHVRDNLITHKHTLEEFAQLVAQHHRSKHESEPDEATIKDWYTKYEQQDDAALQLSEQRIENFLNDAR
L. lanzarotensis Nkp2 MS.EGIVQEFLAQGLLATSLSWPHFQALVTEANE.....KLSSHQIAYVYQQLKIKEEEFVKRSQSRIQEHLIKIR
C. glabrata Nkp2 MSSTDLIIAHFNELRFSDVLSIEDFKEIIIQSKT....VEVHDEDVNKWYQSYLRAEQKKLKLFRERLRIFLASIR
80 90 100 110 120 130
K. lactis Nkp2 ....VELEREQIEDSISLEEVNKLLHSIDDMLQQHLNQLNIELDNKNTKLLKYAMELSDI.E.AID.TSM...YDN
S. cerevisiae Nkp2 SRLLRFLESEQLSHSLSLETLIDALYKINDLLQQRLQILDDAIQEKTSELAEFENMVRSP.S.AGD.NAIPGLLQI
V. polyspora Nkp2 ETQIKELEDLQSSKTFTLEELINHLYSIDQILNIKLKNLDDEISENTVKFKKLNDMILQSNNDTSD.GNS...SAD
Z. rouxii Nkp2 QAQLLELEKSQLAESFSLEDVVNKLYHVDQLLDKRLAYMNESMKDNVTELQKFNELLELANSTKT...DD...DED
L. lanzarotensis Nkp2 SNARDNLEATQLKSTVSVEDLVNTLYSAHQLFDDRTTQLNSDINAYTQKLRIIEEEMRPL.KDAANIQSIQNRLEN
C. glabrata Nkp2 QRELQKLEKEQLSESYDLEEIISSLYKLNEVFEGIVMNQNDELRQKQAELANFKDHLAAS.LDSSD.RSI...LDS
140 150
K. lactis Nkp2 VAKLITDMETNQPSS........
S. cerevisiae Nkp2 IQSYINLLEEN............
V. polyspora Nkp2 ITDTLETIKKYKSMISNDIDDPI
Z. rouxii Nkp2 ISS....................
L. lanzarotensis Nkp2 ...LVEHAKKAQS..........
C. glabrata Nkp2 INSSIEAIEKYRKALDEGS....
Proteolysis sites from our limited proteolysis

E

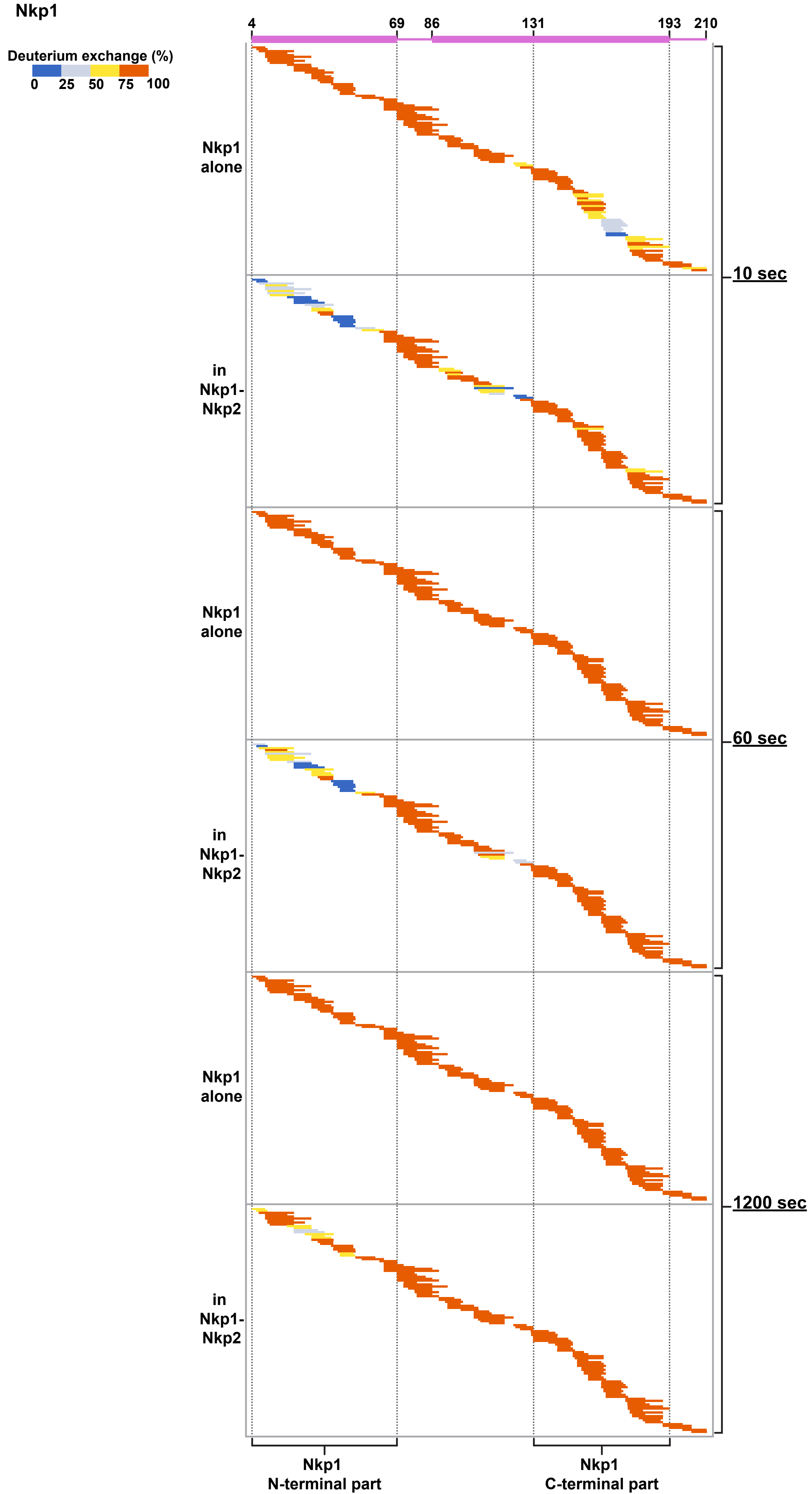
Nkp1
Deuterium exchange (%)
0 25 50 75 100
4 69 86 131 193 210
Nkp1 alone
in Nkp1-Nkp2
10 sec
60 sec
1200 sec
Nkp1 N-terminal part
Nkp1 C-terminal part

F

SEC of reconstituted *K. lactis* Nkp1-Nkp2 truncation variant

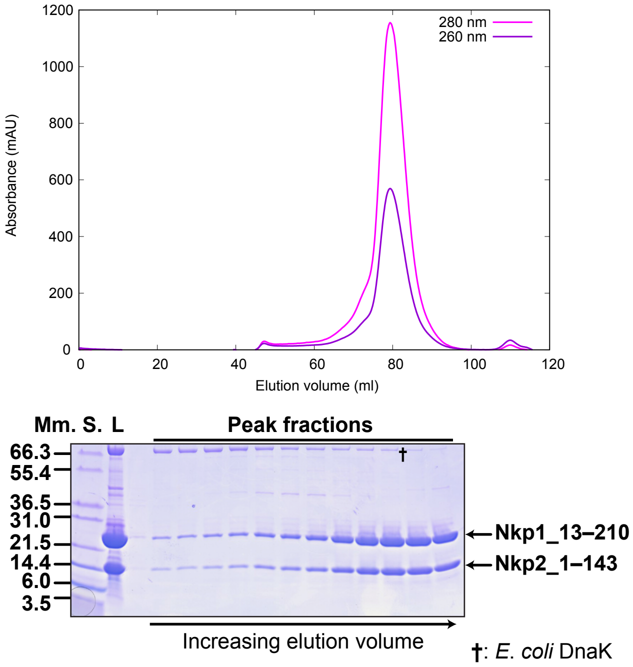

Appendix Figure S6: Binding sites of Okp1, Ctf19, or Mcm21 in COMA

A,B) Representative plots showing deuterium-exchanged peptides after 10 sec, 60 sec, or 1200 sec of deuterium exchange of Okp1 (**A**) or Ame1 (**B**), each in $Ame1_{1–260}$-$Okp1_{123–336}$ or in full COMA. **C)** Representative plots showing deuterium-exchanged peptides, after 10 sec, 60 sec, or 1200 sec of deuterium exchange of Okp1 (versions) in $Ctf19_{D\text{-}RWD}$-$Mcm21_{D\text{-}RWD}$-$Okp1_{229–336}$, in full-length COMA, or in $Ame1_{1–260}$-$Okp1_{123–336}$. **D,E)** Representative plots showing deuterium-exchanged peptides, after deuterium exchange for 10 sec, 60 sec, or 1200 sec of Ctf19 (**D**) or Mcm21 (**E**), each in in Ctf19-Mcm21 or in COMA. The globular D-RWD domains of Ctf19 or Mcm21 are indicated with thicker lines above the plots.

Based on our deuterium-exchange data of Ctf19-Mcm21, we re-assigned electron density for a protein fragment that is not directly connected with the globular Ctf19-Mcm21 D-RWD domains in our previously reported crystal structure of full-length *K. lactis* Ctf19-Mcm21 (Schmitzberger & Harrison, 2012), which we had determined with X-ray diffraction data to 3.9 Å resolution (PDB code 3ZXU). We previously interpreted this density as residues 69–87 of Ctf19. Our deuterium-exchange data, however, show that Ctf19 residues 36–55 are ordered in Ctf19-Mcm21 (see **D**). We thus think it is more plausible this density corresponds to residues 36–54.

Appendix Figure S6

A

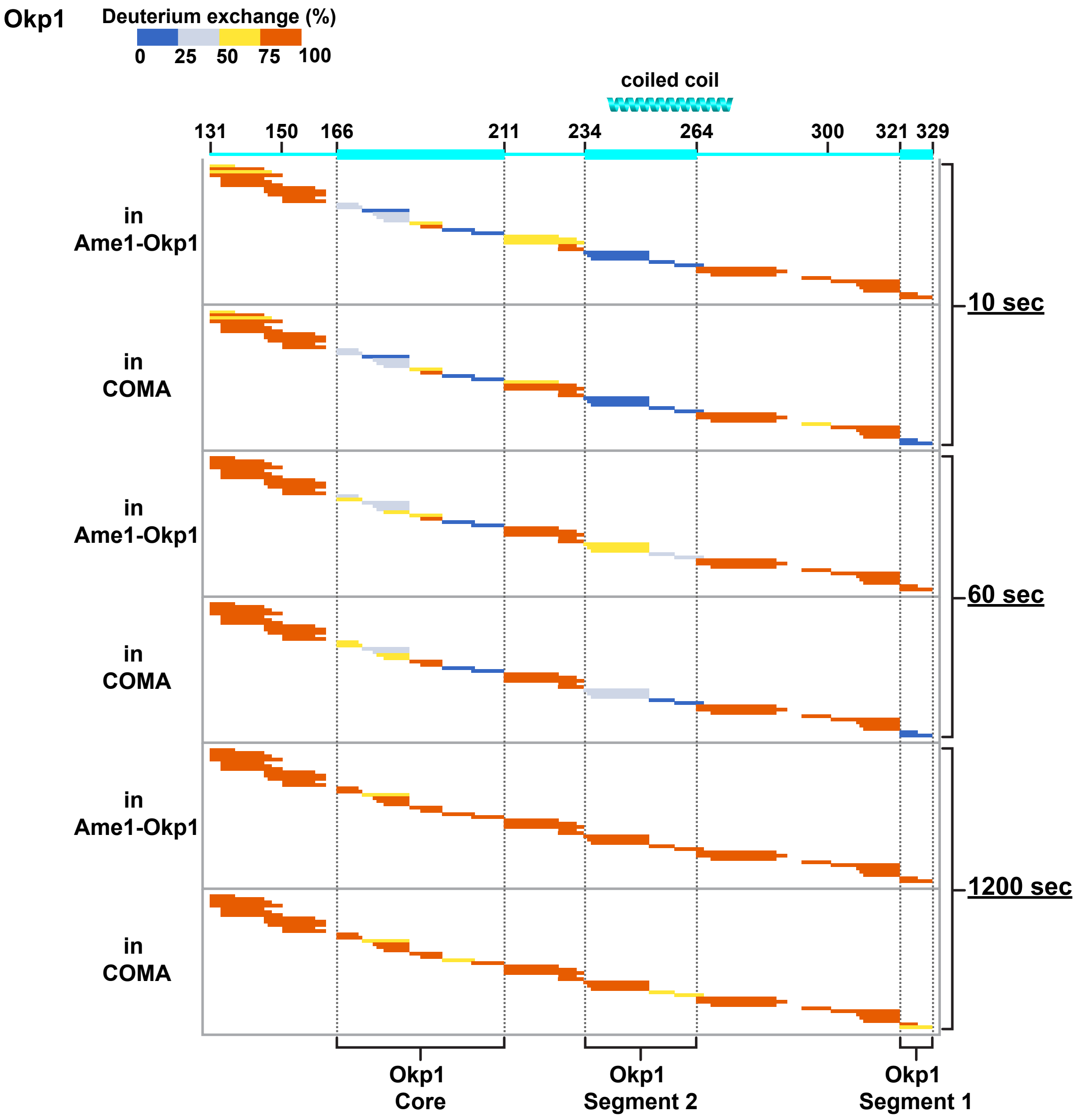

B

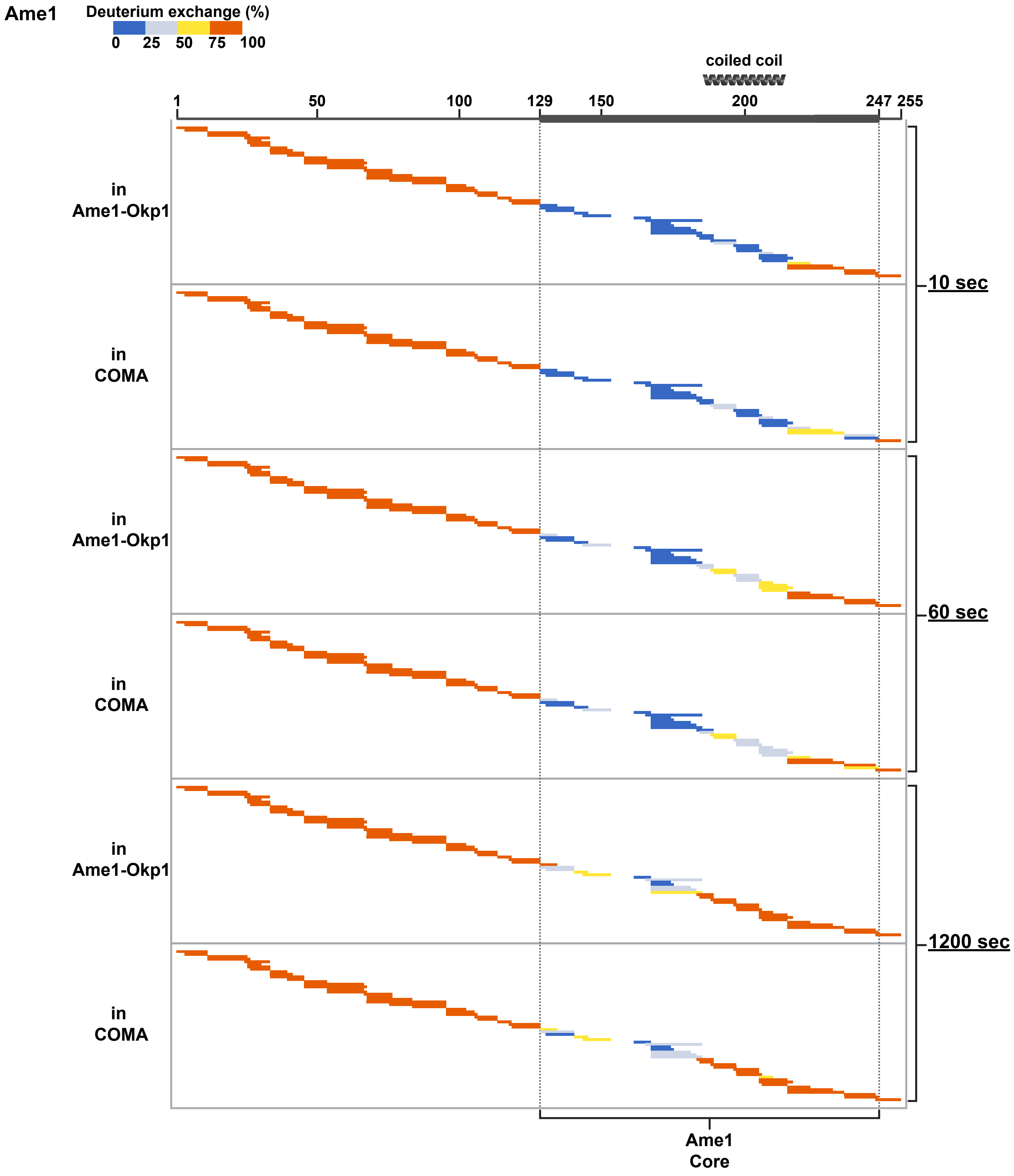

Ame1
Deuterium exchange (%)
0 25 50 75 100
coiled coil
1 50 100 129 150 200 247 255
in Ame1-Okp1
in COMA
in Ame1-Okp1
in COMA
in Ame1-Okp1
in COMA
10 sec
60 sec
1200 sec
Ame1 Core

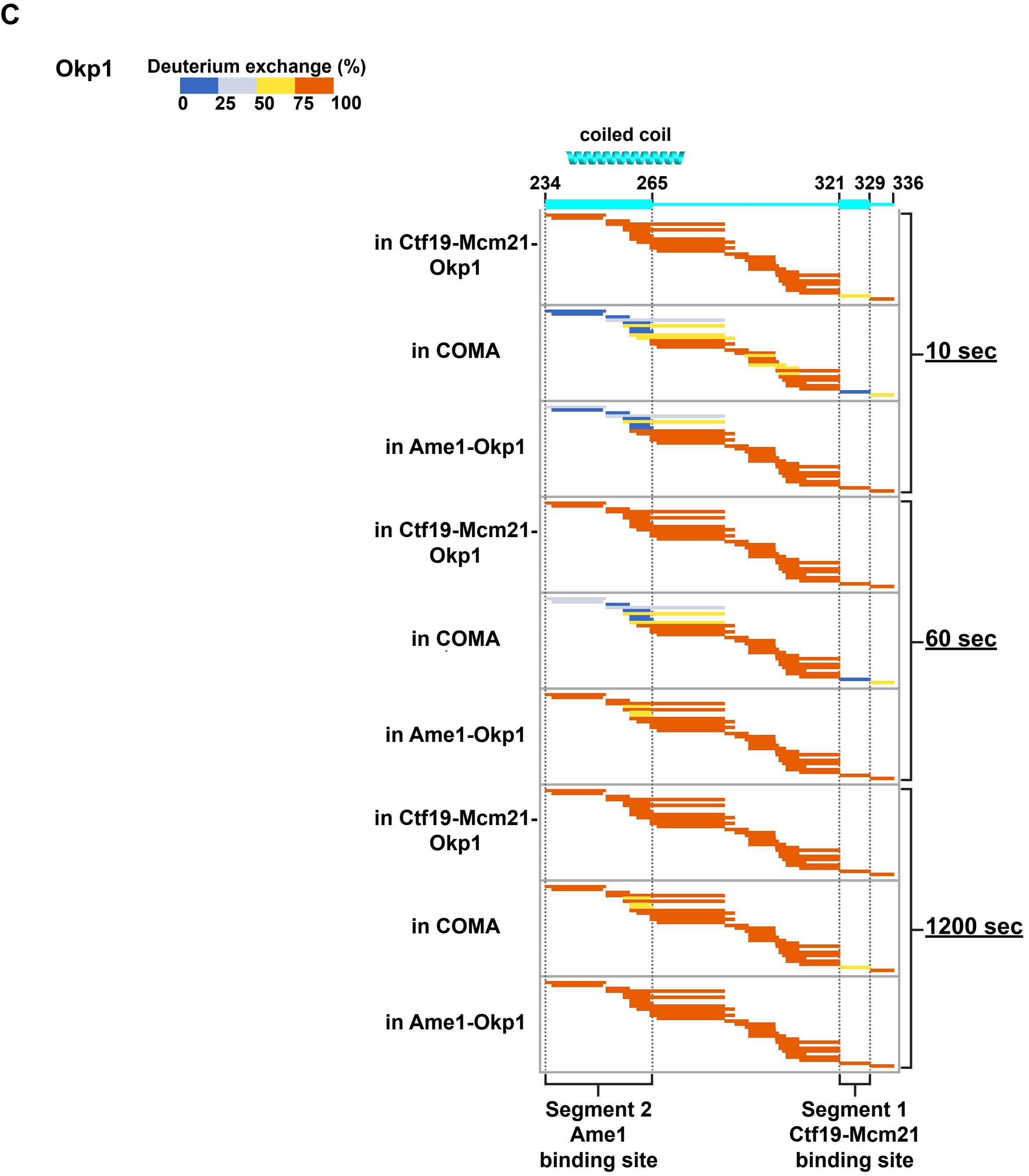
C
Okp1
Deuterium exchange (%)
0 25 50 75 100
coiled coil
234 265 321 329 336
in Ctf19-Mcm21-Okp1
in COMA
in Ame1-Okp1
10 sec
60 sec
1200 sec
Segment 2 Ame1 binding site
Segment 1 Ctf19-Mcm21 binding site

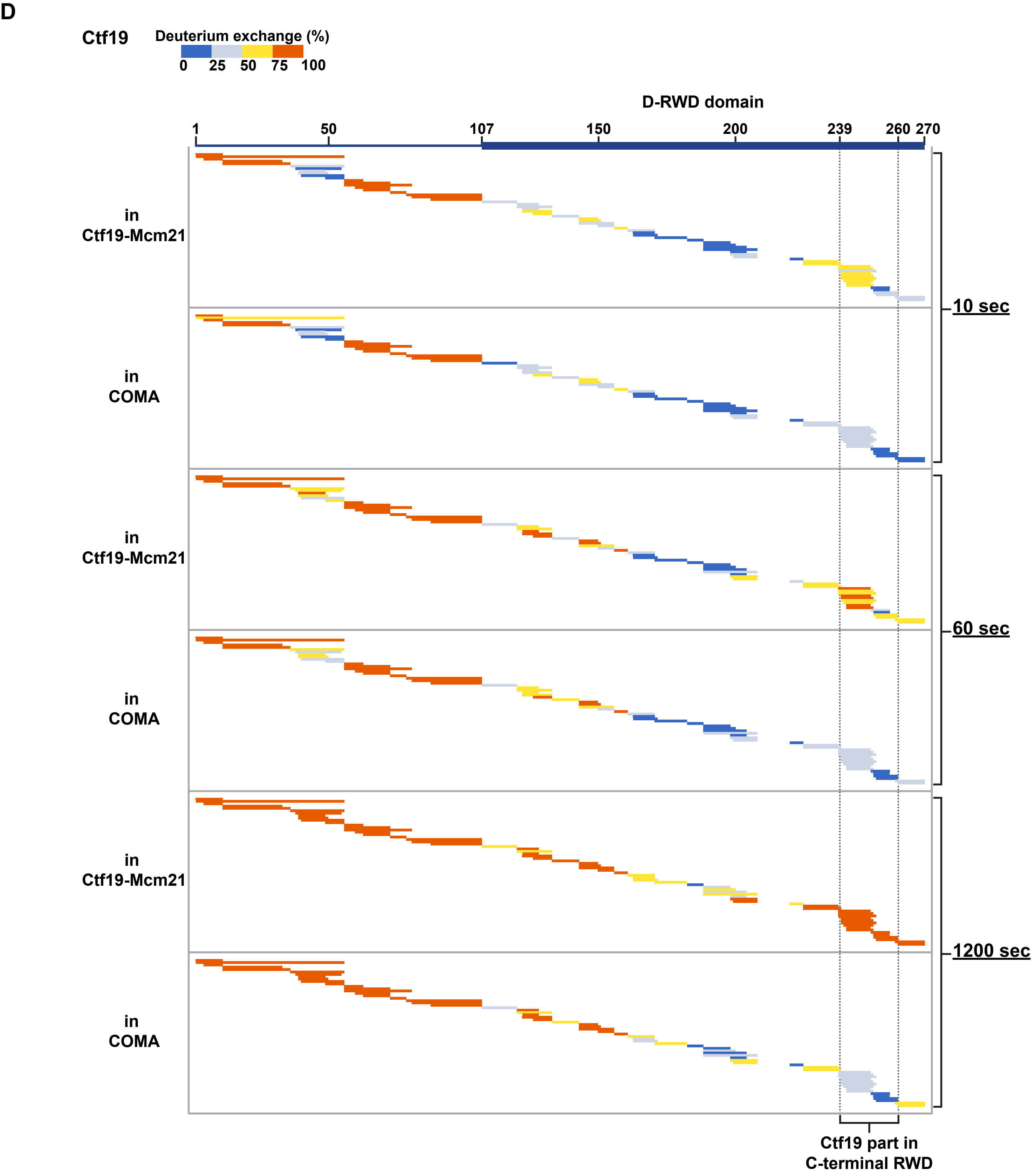
D
Ctf19
Deuterium exchange (%)
0
25
50
75
100
D-RWD domain
1
50
107
150
200
239
260
270
in Ctf19-Mcm21
in COMA
in Ctf19-Mcm21
in COMA
in Ctf19-Mcm21
in COMA
10 sec
60 sec
1200 sec
Ctf19 part in C-terminal RWD

E

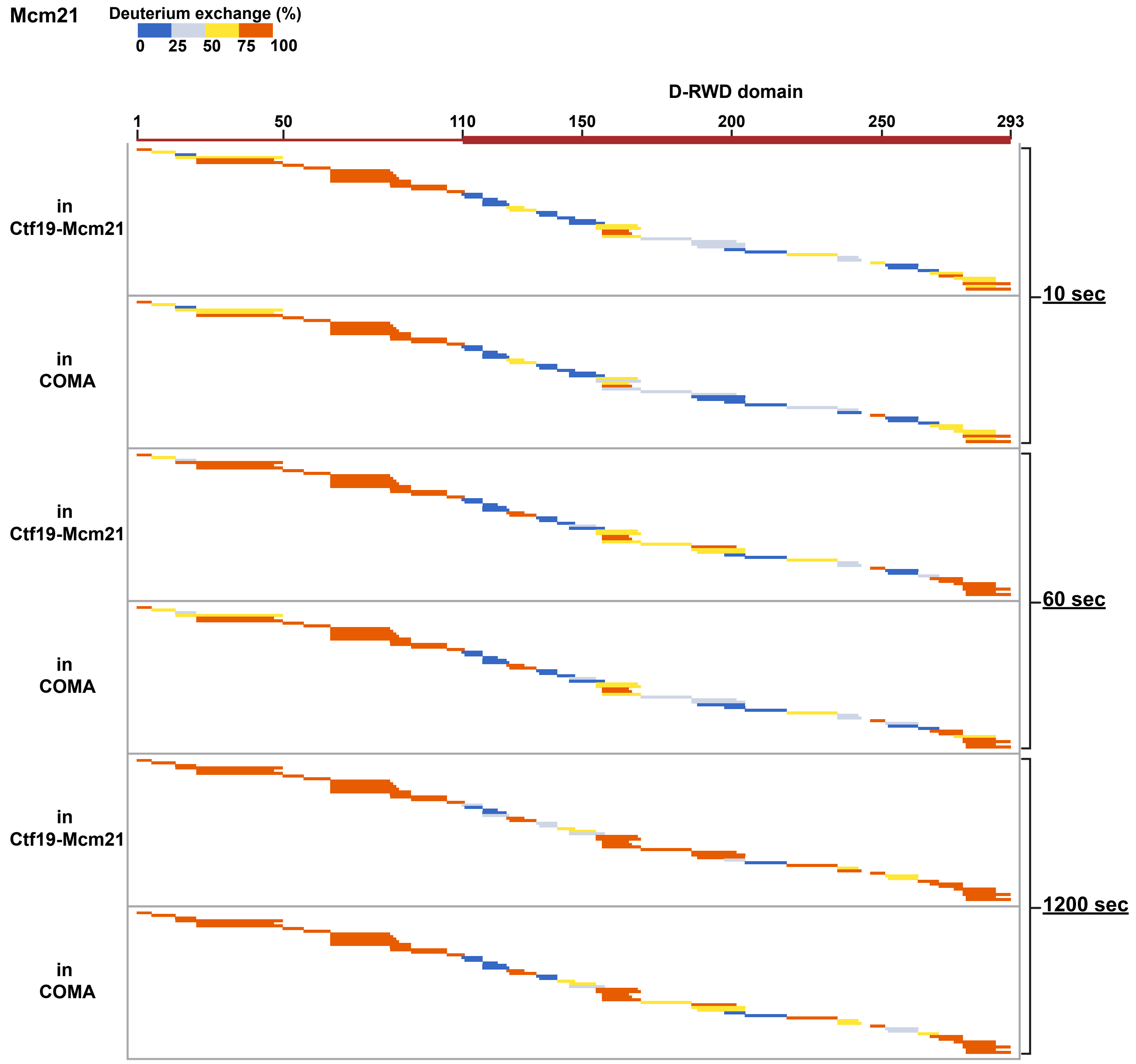
Mcm21
Deuterium exchange (%)
0 25 50 75 100
D-RWD domain
1 50 110 150 200 250 293
in Ctf19-Mcm21
in COMA
in Ctf19-Mcm21
in COMA
in Ctf19-Mcm21
in COMA
10 sec
60 sec
1200 sec

Appendix Figure S7: Reconstituted Ctf19-Mcm21-Okp1 variants that bind Ctf19-Mcm21, or Ctf19-Mcm21 and Nkp1-Nkp2, and crystallographic analyses of crystals of Ctf19-Mcm21 bound with Okp1 segment

A) Representative SEC chromatogram of $Ctf19_{D\text{-}RWD}$-$Mcm21_{D\text{-}RWD}$-$Okp1_{295–360}$, and image of SDS-PAGE gel with fractions from the principal SEC peak; L: sample loaded on column. **B)** Representative overlayed SEC chromatograms showing absorbance at 280 nm of $Ctf19_{D\text{-}RWD}$-$Mcm21_{D\text{-}RWD}$-$Okp1_{295–383}$ (CMO), Nkp1-Nkp2 (NN), or $Ctf19_{D\text{-}RWD}$-$Mcm21_{D\text{-}RWD}$-$Okp1_{295–383}$ combined with Nkp1-Nkp2 (CMO-NN); and image of SDS-PAGE gel with fractions from principal SEC peak of $Ctf19_{D\text{-}RWD}$-$Mcm21_{D\text{-}RWD}$-$Okp1_{295–383}$ combined with Nkp1-Nkp2. Nkp1-Nkp2 dissociated from $Ctf19_{D\text{-}RWD}$-$Mcm21_{D\text{-}RWD}$-$Okp1_{295–383}$ after storage at −80 °C. **C)** Difference Fourier electron-density map of type *mFo−DFc*, for the region around the Okp1 binding site in Ctf19-Mcm21, contoured at 2.7 σ (0.61 e^{-} $Å^{-3}$), that we show as blue mesh, and that we calculated after molecular replacement, automated rebuilding, and refinement of the Ctf19-Mcm21 D-RWD domains only—without modeling any Okp1 residues (thus unbiased for Okp1 fragment) or water molecules. For map calculation, we included X-ray diffraction data to 2.1 Å. We show the Ctf19-Mcm21 D-RWD domains as secondary structure cartoon-diagrams; and some sidechains in stick representation. Viewing perspective is similar as for **Fig 6D**. Two Ctf19-Mcm21-Okp1 assemblies are the asymmetric unit in our crystals. From our diffraction data, we modeled most of the Ctf19-Mcm21 D-RWD domains, except for a few residues at their C-termini, for which no substantial electron density is present. **D)** Image of silver-stained SDS-PAGE gel with fractions from our $Ctf19_{107–270}$-$Mcm21_{108–293}$-$Okp1_{295–360}$ crystals. We washed crystals in three separate drops with crystallization solution. Fractions from these drops are designated W1, W2, W3. After our washes, we dissolved crystals in SDS sample-buffer (lane designate Cr.); a fraction of purified $Ctf19_{107–270}$-$Mcm21_{108–293}$-$Okp1_{295–360}$ we show as control. **E)** Secondary structure cartoon-diagrams of the superposed structures of the D-RWD domains of Ctf19-Mcm21 (PDB code: 3ZXU; orange) and Ctf19-Mcm21 bound with Okp1 (our study; PDB code: 5MU3; colours are as for **Fig 6D**). We superposed the structures using the main-chain atoms of $Ctf19_{D\text{-}RWD}$-$Mcm21_{D\text{-}RWD}$. A few residues we show in stick representation. The relative arrangement of the two Ctf19-Mcm21 dimers that form the asymmetric unit in our $Ctf19_{D\text{-}RWD}$-$Mcm21_{D\text{-}RWD}$-$Okp1_{295–360}$ crystals differs from that of our previously reported crystal structure of full-length Ctf19-Mcm21 (Schmitzberger & Harrison, 2012). This observation suggests that the crystal contacts for both structures are not biologically relevant; i.e. for COMA multimerization. We observe structural differences between the two Ctf19-Mcm21 structures mostly in the C-terminal RWD domains (RWD-C). Because the Mcm21 D-RWD domains are structurally more similar between the two structures, and the differences for Ctf19 RWD-C are present for both molecules of the asymmetric unit, we assume the change in conformation in Ctf19 RWD-C with Okp1 bound relative to Ctf19 that is not bound with Okp1, is due to Okp1 binding. This assumption is consistent with the change in the hydrogen-bonding network of Ctf19 RWD-C that our hydrogen-deuterium exchange data show (**Fig 6B**).

Appendix Figure S7

A

SEC of Ctf19$_{D\text{-}RWD}$-Mcm21$_{D\text{-}RWD}$-Okp1 variant with Okp1 segment 1

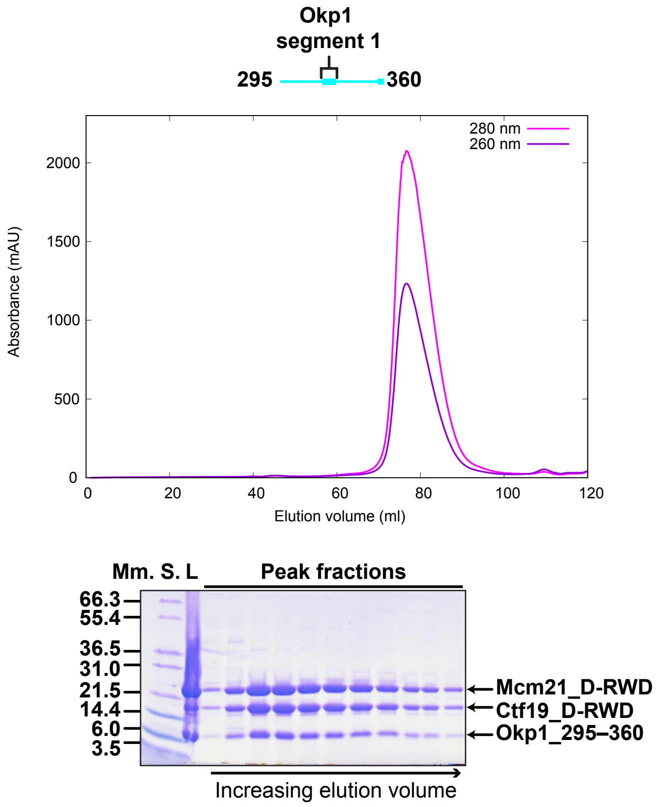

B

SEC of Ctf19$_{D\text{-}RWD}$-Mcm21$_{D\text{-}RWD}$-Okp1 variant with Okp1 segment 1 and Okp1 segment 3 + Nkp1-Nkp2

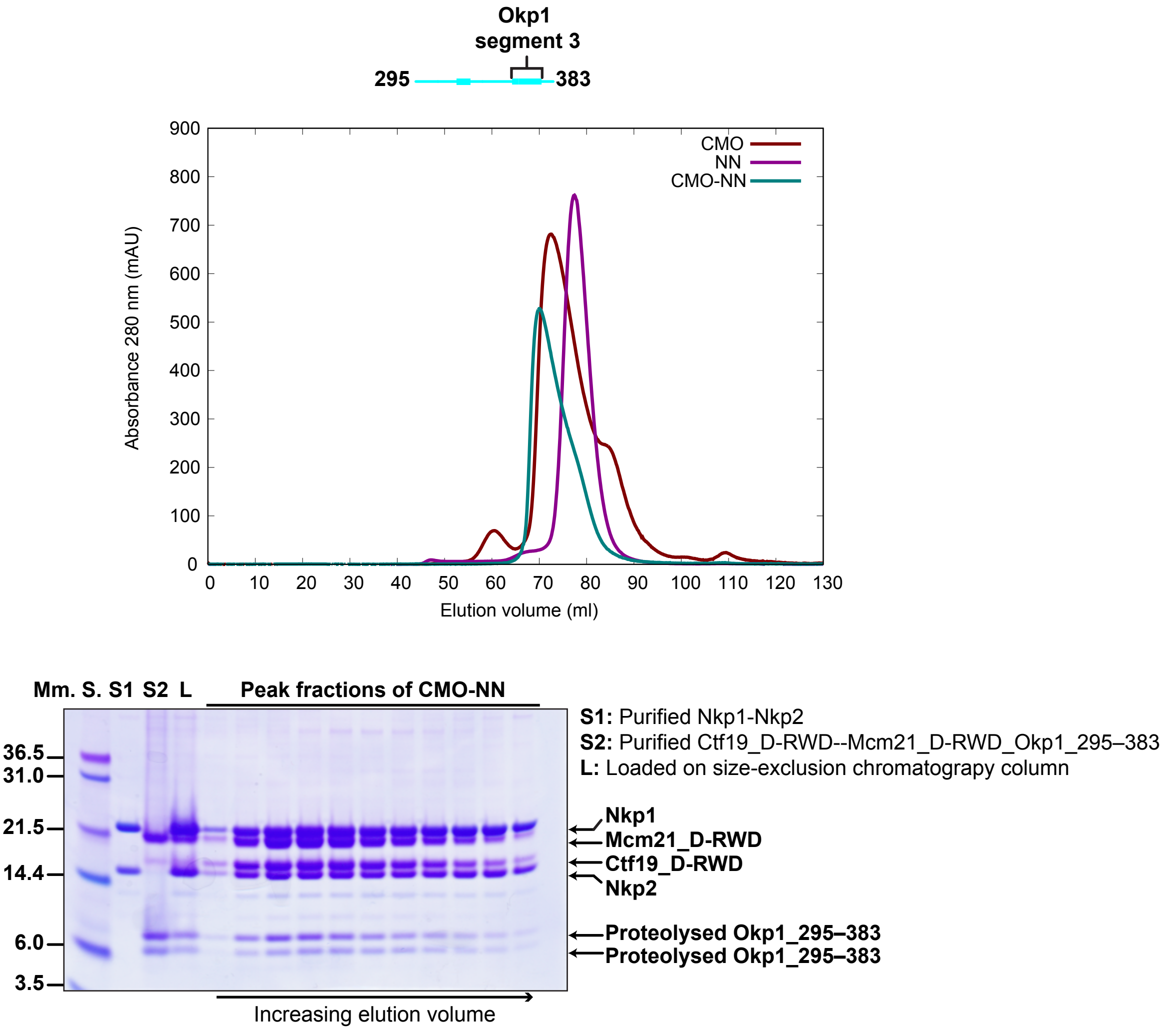

C

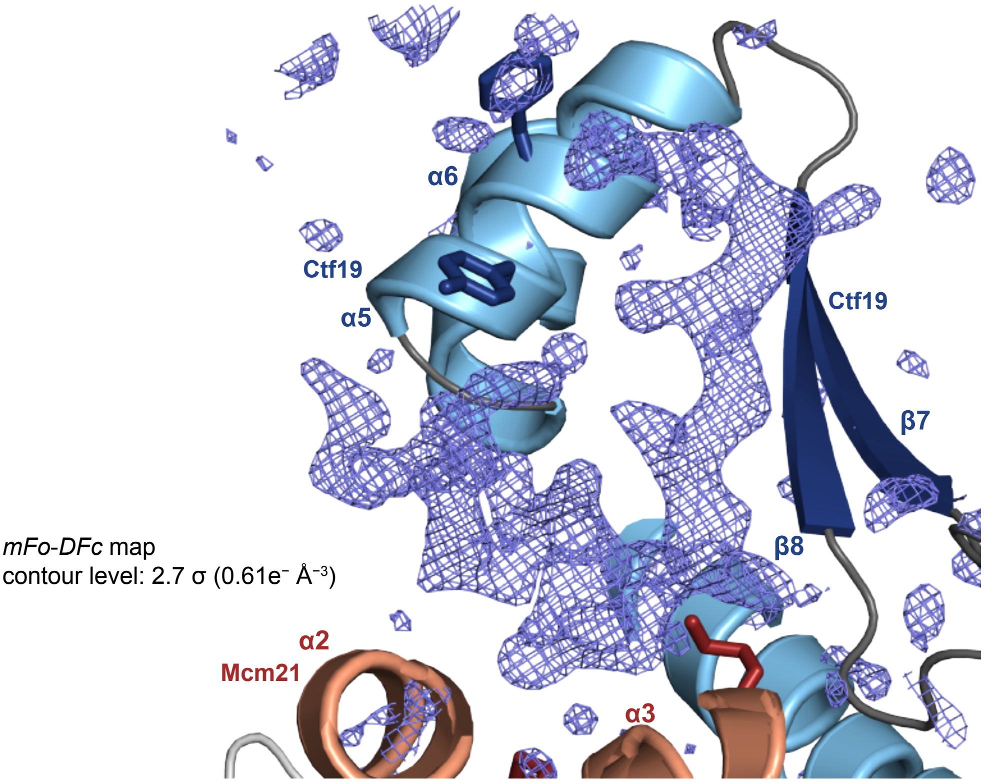

D

Silver-stained SDS-PAGE of dissolved Ctf19$_{D\text{-}RWD}$-Mcm21$_{D\text{-}RWD}$-Okp1$_{295–360}$ crystals

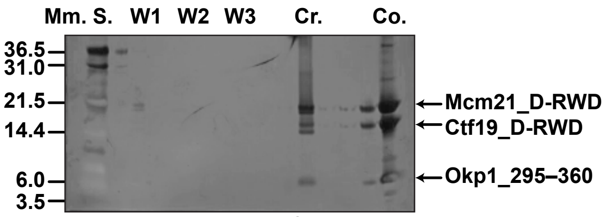

W1, W2, W3: Fractions from serial crystal washes
Cr.: Dissolved crystal
Co: Purified Ctf19_D-RWD--Mcm21_D-RWD--Okp1_295–360

E

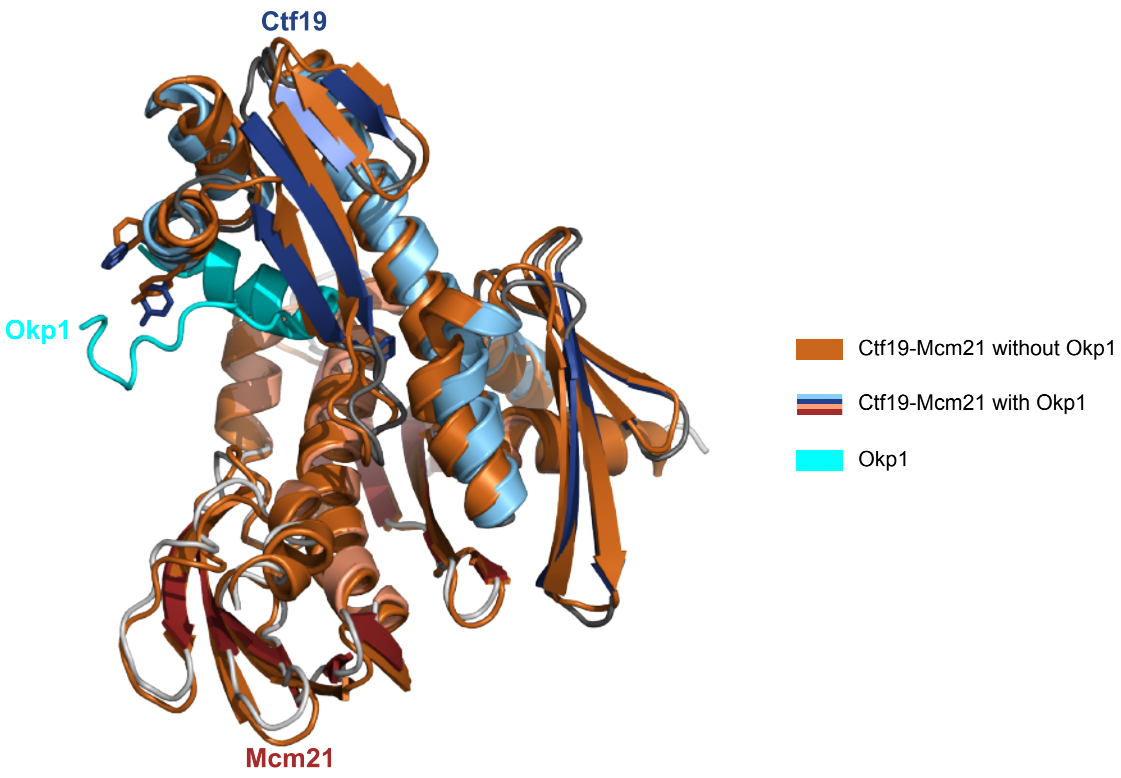

Appendix Figure S8: Relevance of Ctf19-Mcm21 binding motif for COMA assembly and inner kinetochore subunit-interactions

A) Representative image of SDS-PAGE gel with elution fraction (E) from Ni^{2+}-affinity purification of *K. lactis* COMA with Okp1_cmΔ, with polyhistidine-tagged Mcm21; showing that no substantial amount of Okp1_cmΔ-Ame1 co-purifies with Ctf19-Mcm21. Gel lane was deliberately overloaded with sample (to potentially see residual Okp1_cmΔ-Ame1). Migrating position of full-length Okp1 in full COMA is indicated (compare with gel image in **Fig 1A**). **B,C)** Representative images of Western blots of immunoprecipitated fractions from *S. cerevisiae* extracts with Okp1 versions C-terminally tagged with six flag epitopes (6×flag) and Ctf19 C-terminally tagged with four myc epitopes (4×myc). We show blots of extracts from *S. cerevisiae* that have either full-length Okp1 (fl) or Okp1 that lacks the Ctf19-Mcm21 binding motif (cmΔ). We used magnetic Protein G coupled beads that were not coated with anti-flag antibodies as a control. Images of Ponceau S-stained nitrocellulose membranes that we used for Western blots we show below the respective blot images. Full-length Ctf19 was present in lower amount from *S. cerevisiae* extracts with our *Okp1_cmΔ* mutant than from clones with *Okp1_fl*. We could not produce substantial amounts of recombinant *K. lactis* Ctf19-Mcm21 D-RWD domains in the absence of Okp1 (variants). We conclude from these observations and the Ctf19-Okp1 contacts in our structure that Ctf19 and Okp1 stabilize each other. **D)** Representative microscopy images of living haploid *S. cerevisiae* cells with C-terminally GFP-tagged kinetochore subunits Mcm16, Mcm22, Wip1, or Nnf1 that have either Okp1_fl or Okp1_cmΔ. Fluorescence images are deconvoluted and maximum intensity projected. We show fluorescence images for the same fluorophor-fusion kinetochore protein in *Okp1_fl* or *Okp1_cmΔ* on the same intensity scale. Image pixels for images of cells with Mcm16-GFP, Mcm22-GFP, or Wip1-GFP are 2×2 binned; scale bar: 2 µm. **E,F)** Representative images of anti-flag (**E**) Western blot or anti-myc (**F**) Western blot of cell extracts of immunoprecipitated fractions from *S. cerevisiae* clones (see Appendix materials and methods for details). We prepared extracts from our native laboratory *S. cerevisiae* clone (S288C type; identifier DDY904 in **Table EV6**) without additional genetic modifications, or clones with an Okp1 version C-terminally tagged with six flag epitopes (Okp1-6×flag) and Mcm16 C-terminally tagged with thirteen myc epitopes (Mcm16-13×myc), after metaphase growth-arrest with nocodazole. We show blots of extracts from *S. cerevisiae* that have either Okp1_fl or Okp1_cmΔ. Filled circles indicate presence of Okp1_fl-6×flag, Okp1_cmΔ-6×flag, or Mcm16-13×myc. Mcm16-13×myc from the cell-extract fraction migrated at a lower position on our SDS-PAGE gel than Mcm16-13×myc that immunoprecipitated (and was thus purified) with Okp1_fl; an effect that we observed frequently.

Appendix Figure S8

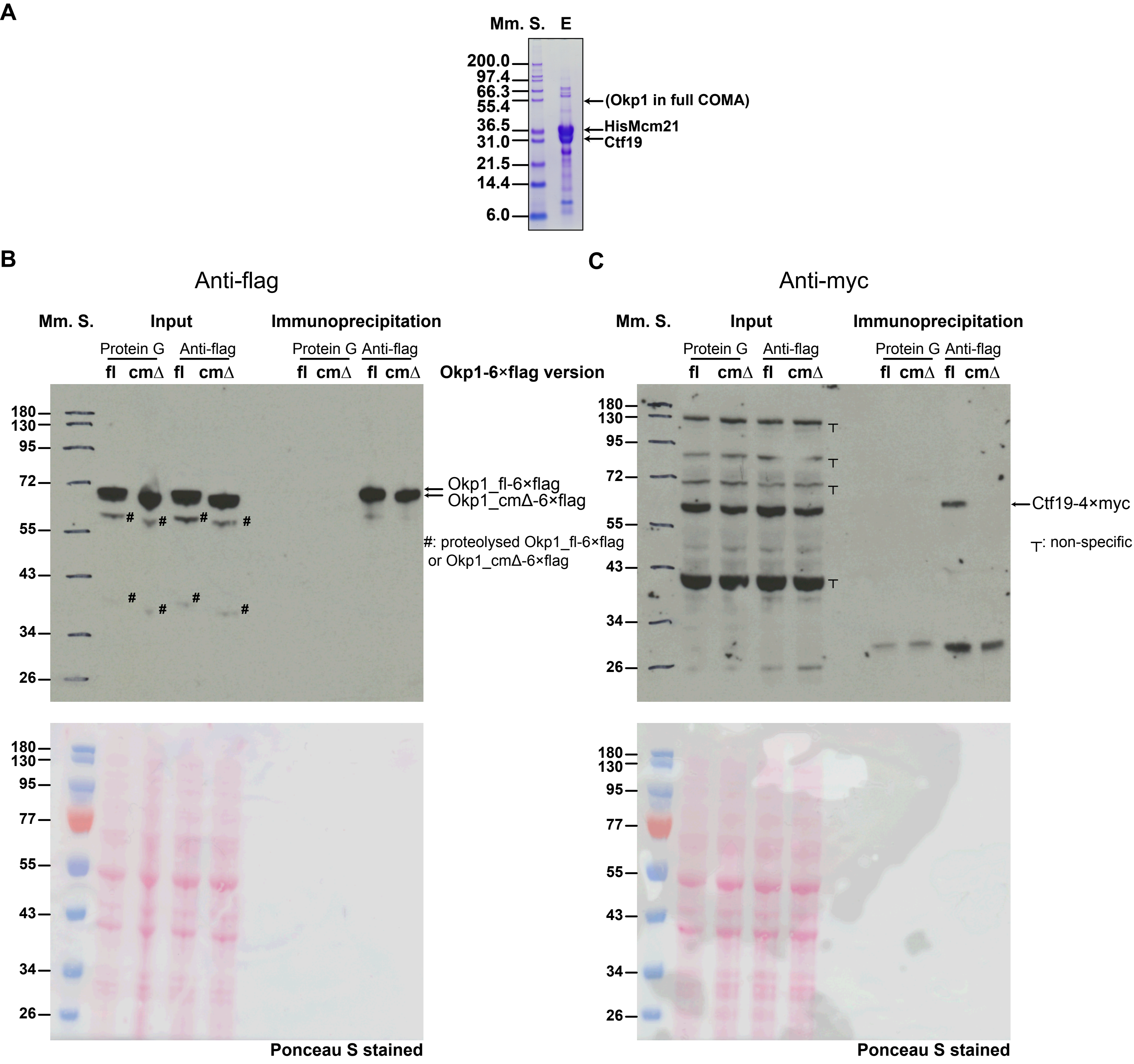

D

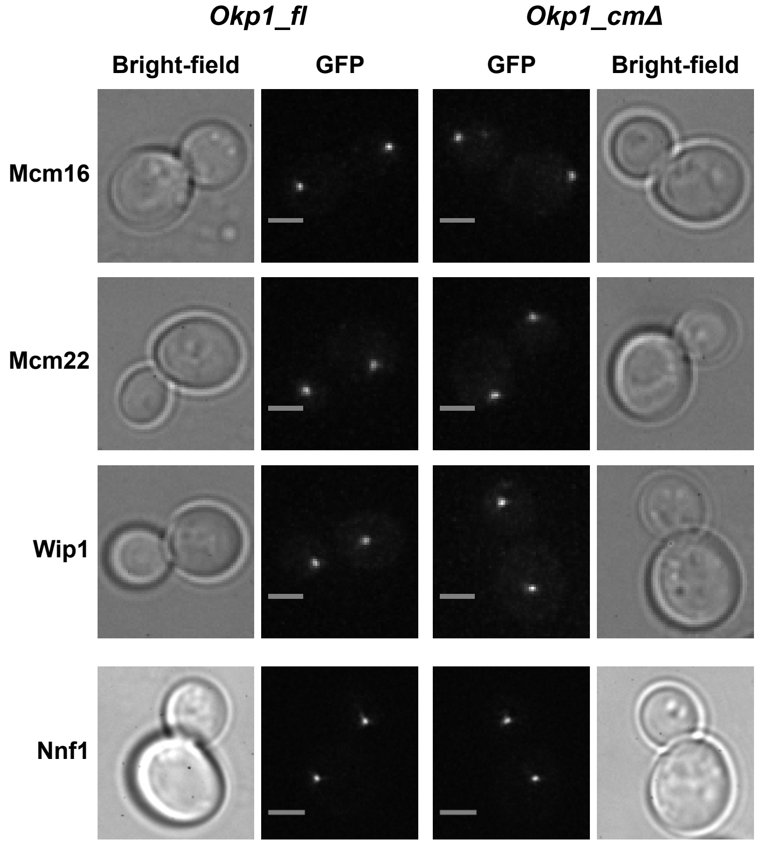

Okp1_fl
Okp1_cmΔ
Bright-field
GFP
GFP
Bright-field
Mcm16
Mcm22
Wip1
Nnf1

E

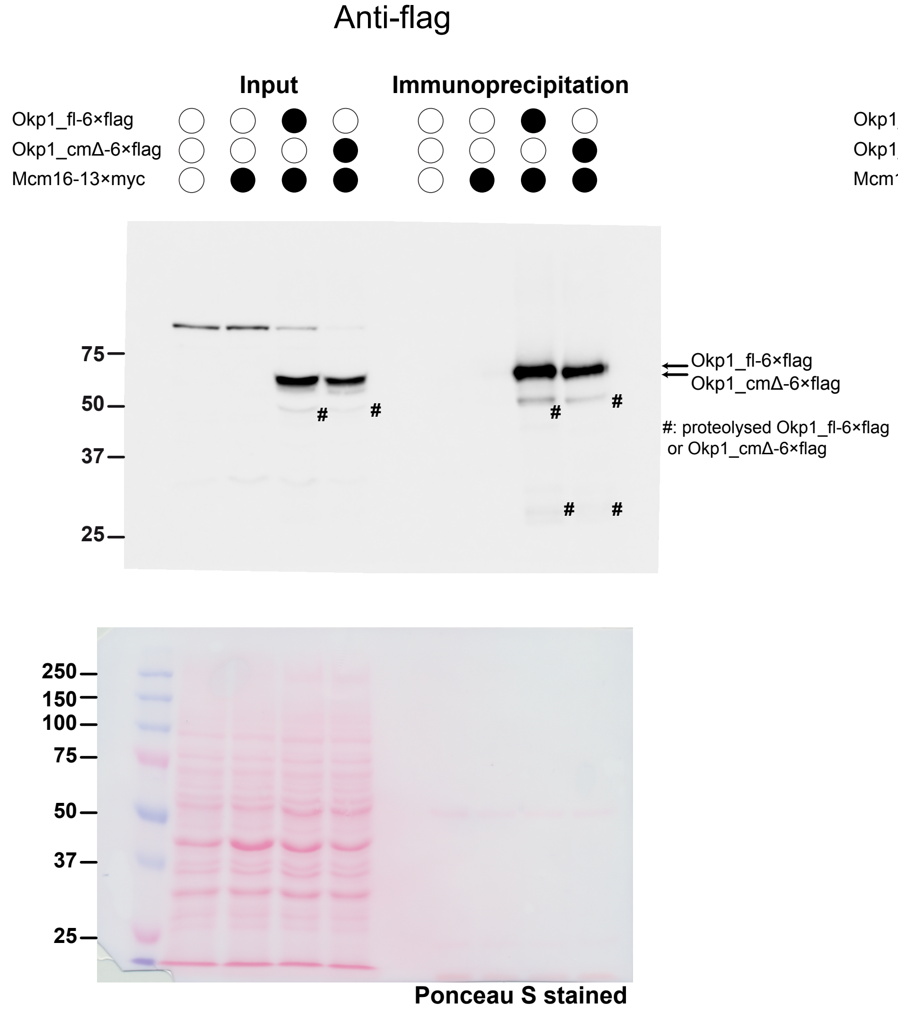

Anti-flag
Input
Immunoprecipitation
Okp1_fl-6×flag
Okp1_cmΔ-6×flag
Mcm16-13×myc
75
50
37
25
Okp1_fl-6×flag
Okp1_cmΔ-6×flag
#: proteolysed Okp1_fl-6×flag or Okp1_cmΔ-6×flag
250
150
100
75
50
37
25
Ponceau S stained

F

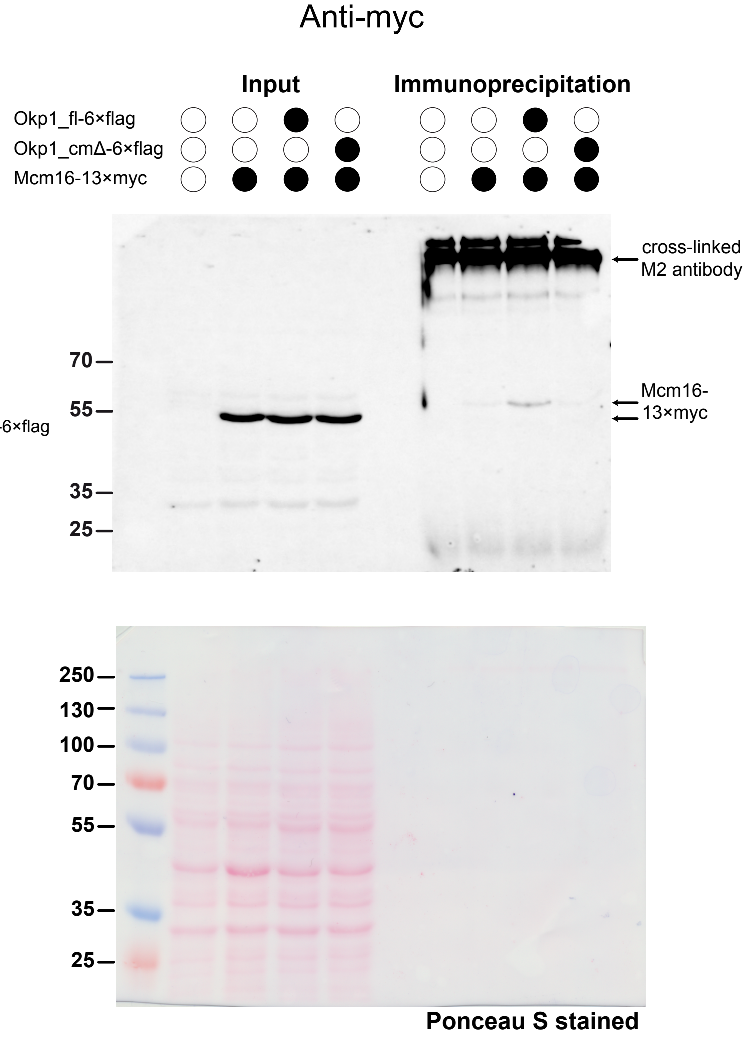

Anti-myc
Input
Immunoprecipitation
Okp1_fl-6×flag
Okp1_cmΔ-6×flag
Mcm16-13×myc
cross-linked M2 antibody
70
55
35
25
Mcm16-13×myc
250
130
100
70
55
35
25
Ponceau S stained

Appendix Figure S9: Relevance of Ctf19-Mcm21 binding motif for cell viability, chromatid segregation, or chromosome segregation

A) Representative images of dilution-series growth assays of haploid *S. cerevisiae* clones with *Okp1_fl, Okp1_cmΔ, Okp1_cmΔnnΔ,* or *Okp1_nnΔ*, after two days of growth on solid YPD agar at 30 °C, or after three days of growth on solid YPD agar with 20 µg ml^{-1} benomyl at 40 °C. We show growth from two unique clones each for *Okp1_fl, Okp1_cmΔ,* or *Okp1_cmΔnnΔ*. We sequentially diluted *S. cerevisiae* cells four fold; similarly as for the dilution-series assays that we show images of in **B** and **E**. Arrow indicates decreasing cell density. **B)** Representative images of dilution-series growth assays of haploid *S. cerevisiae* clones with *ctf3Δ*, *Okp1_fl, Okp1_cmΔ*, *Okp1_fl ctf3Δ*, or *Okp1_cmΔ ctf3Δ*, after two days of growth on solid YPD agar at 25 °C, or after three days of growth on solid YPD agar with 20 µg ml^{-1} benomyl at 40 °C. We show two unique clones each for *Okp1_fl ctf3Δ* or *Okp1_cmΔ ctf3Δ*. **C)** Sister-chromatid mis-segregation rates from chromosome V in mitotically cycling, haploid *S. cerevisiae* with *Okp1_fl* or *Okp1_cmΔ*, as judged by chromosome V GFP-signals in living large budded cells. **D)** Representative microscopy images of living haploid *S. cerevisiae* cells with C-terminally 3×GFP-tagged Bub1 either in *Okp1_fl* or *Okp1_cmΔ*. Fluorescence images are maximum intensity projected. We show fluorescence images of *Okp1_fl* or *Okp1_cmΔ* on the same scale; scale bar (lower left corner): 2 µm. **E)** Representative images of dilution-series growth assay of haploid *S. cerevisiae* clones—a native clone (native; S288C type; identifier DDY904 in **Table EV6**), *Okp1_cmΔ* with genomically encoded OsTIR1 (*Okp1_cmΔ OsTIR1*), a native clone with OsTIR1 (*OsTIR1)*, two unique clones of *Okp1_fl* with OsTIR1 and Mad1-degron-9×myc (*mad1-degron-9×myc OsTIR1 Okp1_fl*), or two unique clones of *Okp1_cmΔ* with OsTIR1 and Mad1-degron-9×myc (*mad1-degron-9×myc OsTIR Okp1_cmΔ*), on solid raffinose-galactose agar with 1 mM of the auxin analogue 1-napthylic acetic acid (NAA), which induces degradation of Mad1-degron-9×myc. **F)** Representative Western-blot image of *S. cerevisiae* extracts from cultures grown in liquid raffinose-galactose medium at 30 °C of a native laboratory *S. cerevisiae* (native; S288C type; identifier DDY904 in **Table EV6**), or clones with *mad1-degron-9×myc OsTIR1 Okp1_fl* (identical clone as the one further towards the top of the two that we show growth of in E), or with *mad1-degron-9×myc OsTIR1 Okp1_cmΔ* (identical clone as the one further towards the top of the two that we show growth of in E). We prepared samples from cultures either from before addition of NAA or from specific time points after addition of NAA. We used extracts from a *S. cerevisiae* clone with *Ndc80-13×myc* as a control (lane 1). We show image of Ponceau S-stained nitrocellulose membrane used for Western blot below the blot image. The higher level of Mad1-degron-9×myc, after addition of NAA, in extracts from *Okp1_cmΔ* with OsTIR1 and Mad1-degron-9×myc, compared with that in extracts from *Okp1_fl* with OsTIR1 and Mad1-degron-9×myc, indicates up-regulation of transcription of *mad1-degron-9×myc* or translation of Mad1-degron-9×myc in *Okp1_cmΔ* cells. This observation is consistent with our growth assays on solid medium that we show in **E**. The growth phenotype of *mad1-degron-9×myc OsTIR1 Okp1_cmΔ* is not as pronounced as that of our *mad1Δ Okp1_cmΔ* clone (see **Fig 8C**), indicating that mitotic checkpoint activity is still present. **G)** Evaluation of chromosome mis-segregation in meiosis. Typical images of haploid spores from tetrad dissection of homozygous diploid *S. cerevisiae* with *Okp1_fl/Okp1_fl*, *Okp1_cmΔ/Okp1_cmΔ*, or *ctf19_1–954Δ/ctf19_1–954Δ*, after two days of growth on solid YPD agar, showing spore viability after meiosis. We placed the four spores from tetrads column-wise. Perhaps reflecting a differential centromere-localization

dependence of Ctf19-Mcm21 in meiosis, compared with its dependence on Okp1(-Ame1) for localization in mitosis, ribosomal profiling showed that Ctf19 and Mcm21 are made early in meiosis 1, while most of Ame1 or Okp1 are made in meiosis 2 (Miller et al, 2012).

Appendix Figure S9

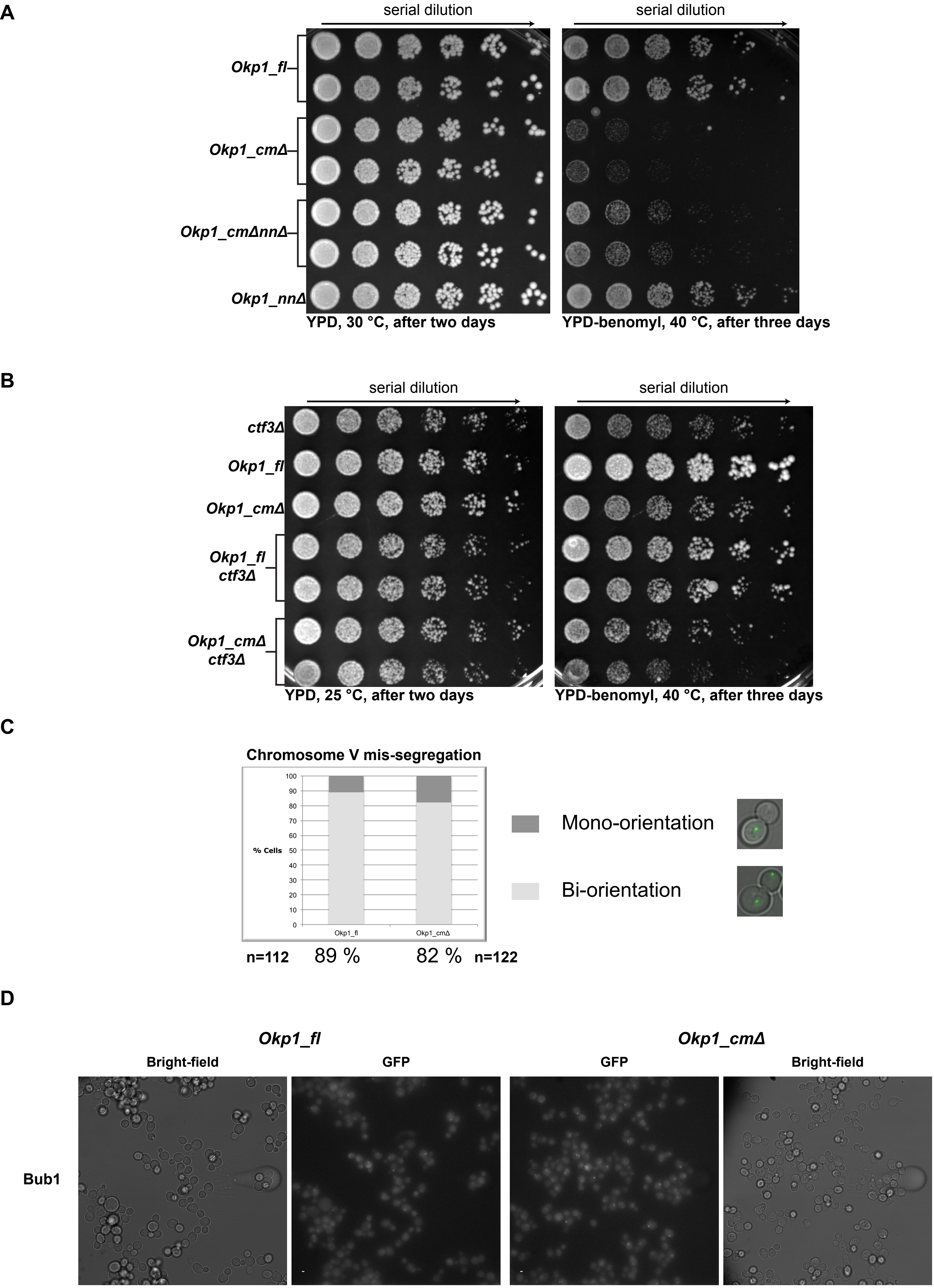

E

Conditional auxin-dependent removal of Mad1

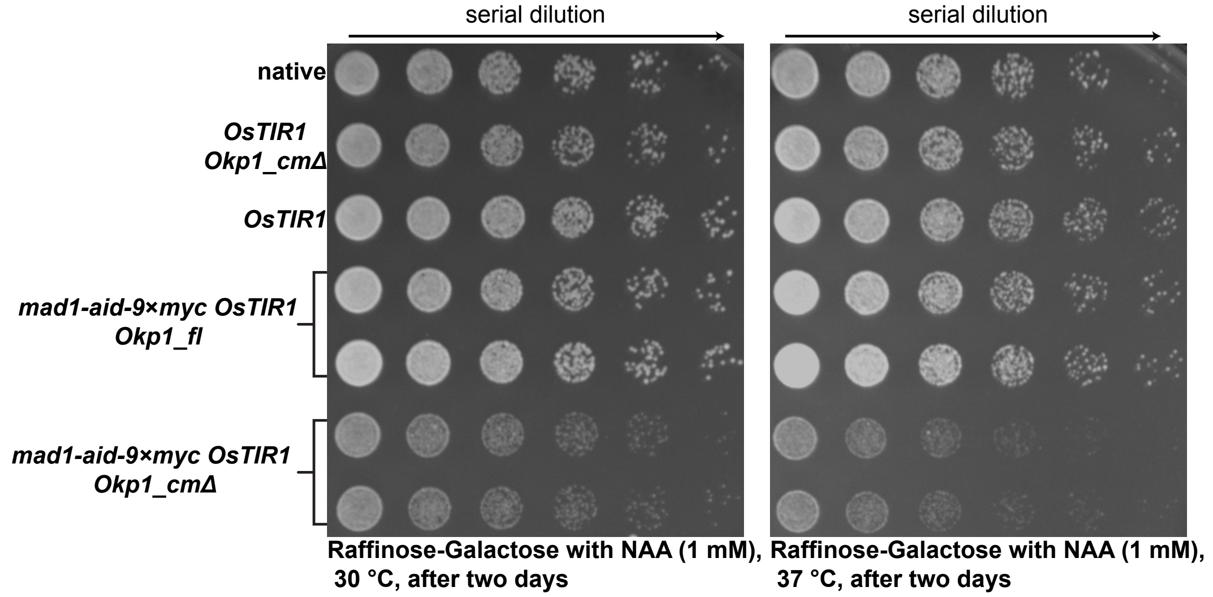

F

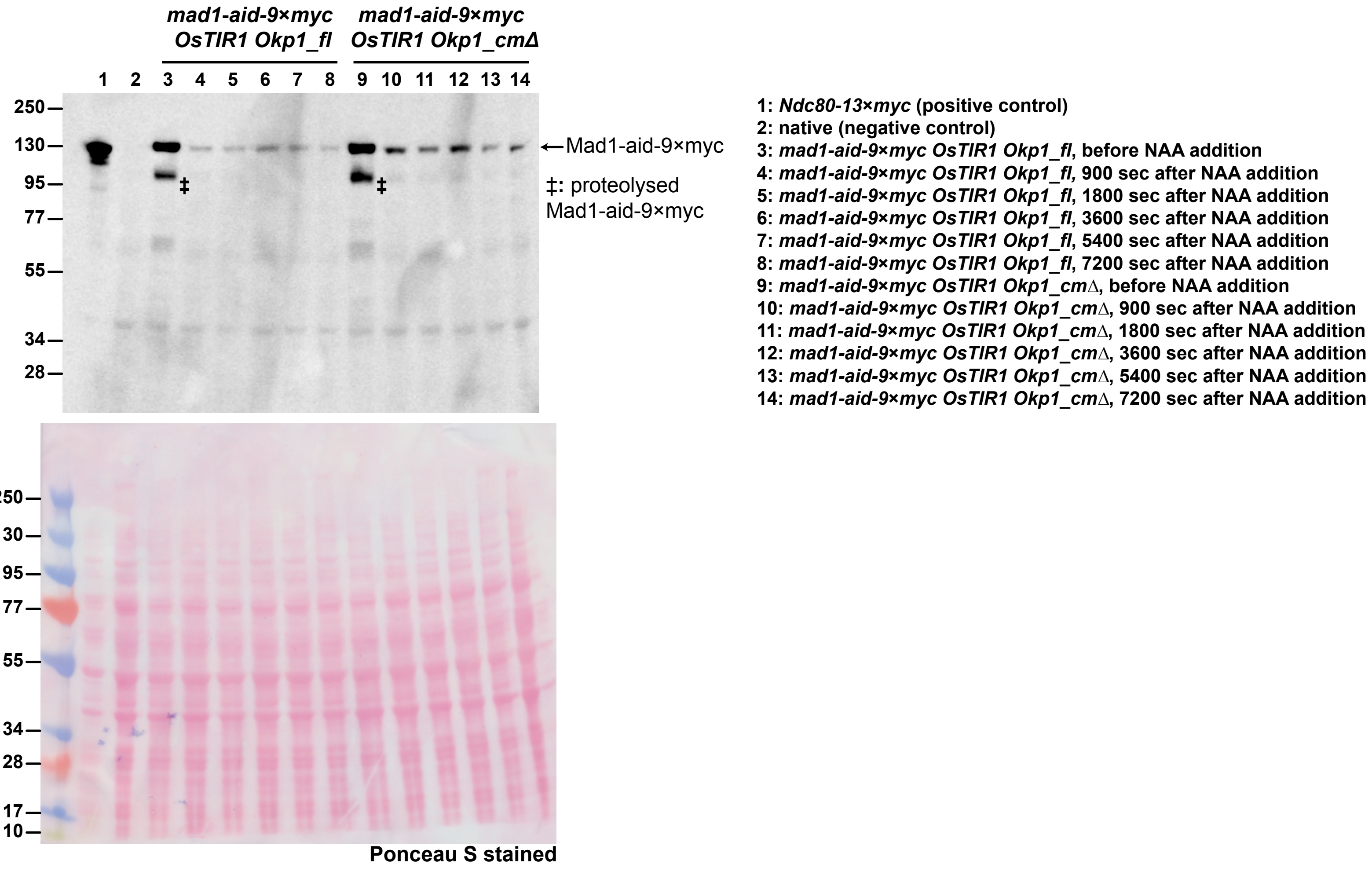

G

Spores from dissection tetrads of homozygous diploid *S. cerevisiae* clones

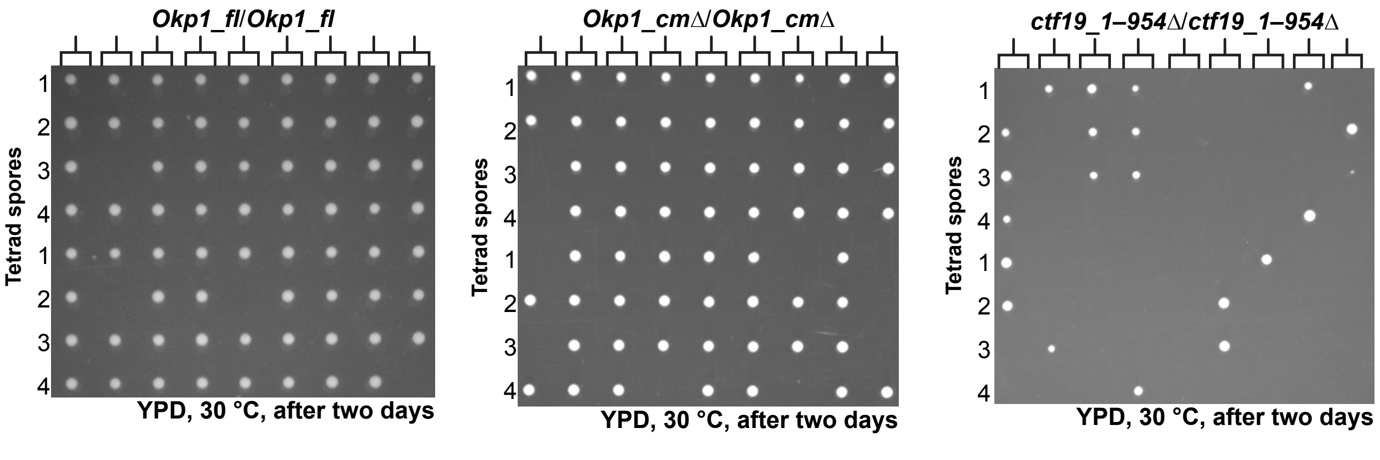

Appendix Figure S10: Multiple sequence alignment of Ame1 proteins from yeasts and CENP-U proteins from animals

Multiple sequence alignment of Ame1 orthologues from budding yeasts (sequences and annotations are as in **Appendix Fig S3F**), *Schizosaccharomyces pombe* Mis17 (Refseq accession-code: NP_001018835.1), and CENP-U orthologues from *Homo sapiens* (Q71F23), *Rattus norvegicus* (NP_001020844.1), *Bos taurus* (XP_005226066.2), *Gallus galllus* (NP_001034388.1). Alignment formatting is analogous to that for alignment that we show in **Appendix Fig S3F**.

Appendix Figure S10

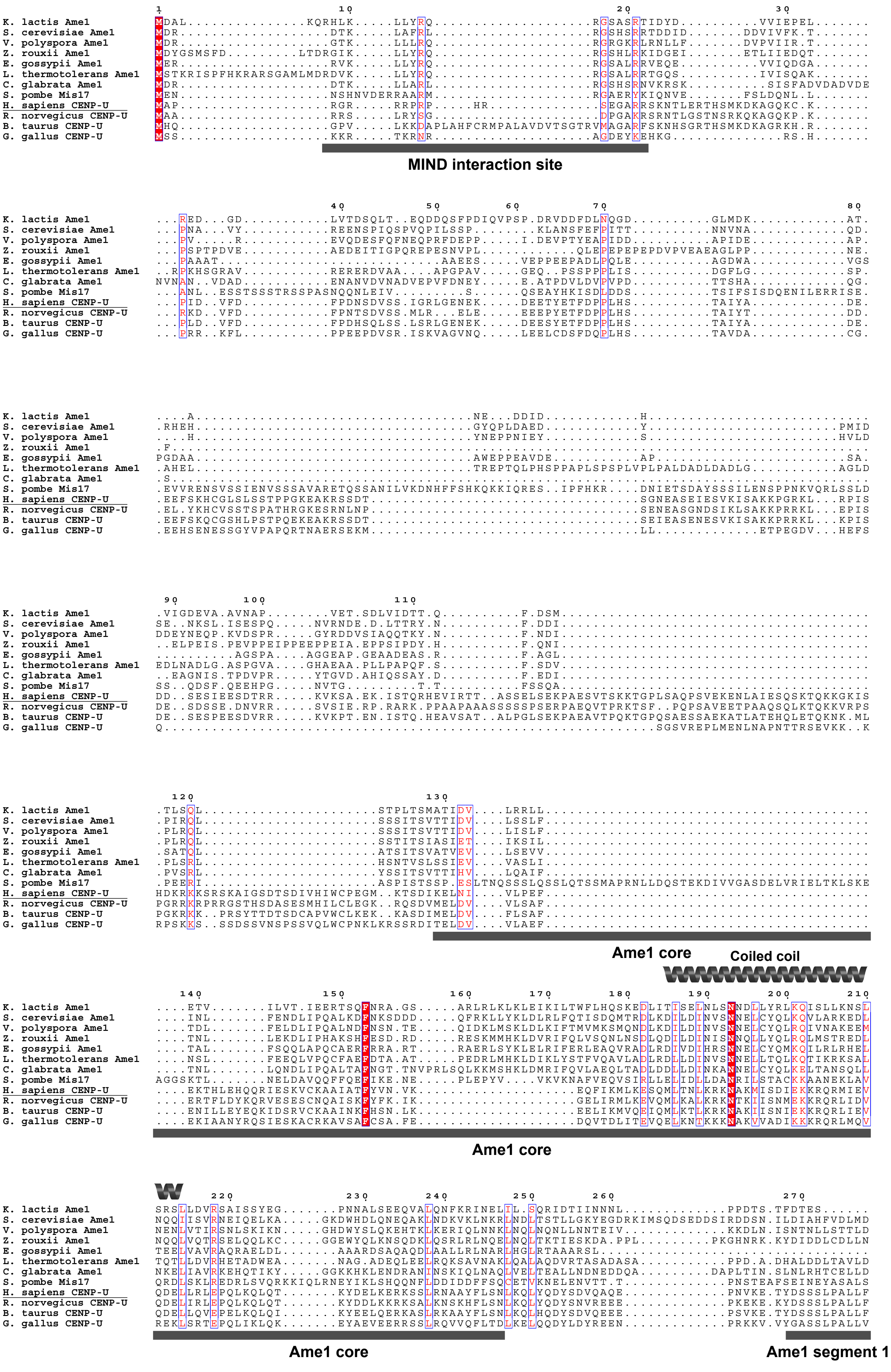

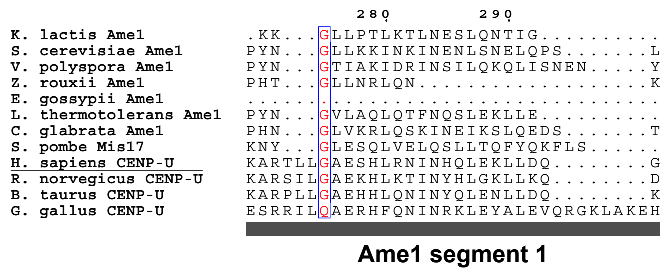
280
290
K. lactis Ame1 .KK...GLLPTLKTLNESLQNTIG..........
S. cerevisiae Ame1 PYN...GLLKKINKINENLSNELQPS.......L
V. polyspora Ame1 PYN...GTIAKIDRINSILQKQLISNEN.....Y
Z. rouxii Ame1 PHT...GLLNRLQN..................K
E. gossypii Ame1 ....
L. thermotolerans Ame1 PYN...GVLAQLQTFNQSLEKLLE..........
C. glabrata Ame1 PHN...GLVKRLQSKINEIKSLQEDS.......T
S. pombe Mis17 KNY...GLESQLVELQSLLTQFYQKFLS......
H. sapiens CENP-U KARTLLGAESHLRNINHQLEKLLDQ........G
R. norvegicus CENP-U KARSILGAEKHLKTINYHLGKLLKQ........D
B. taurus CENP-U KARPLLGAEHHLQNINYQLENLLDQ........K
G. gallus CENP-U ESRRILQAERHFQNINRKLEYALEVQRGKLAKEH
Ame1 segment 1

Appendix supplementary material and methods

Chemicals, reagents, and consumables

For our experiments, we used high-grade chemicals from Sigma-Aldrich (Merck KGaA), Merck Millipore (Merck KGaA), Thermo Fisher Scientific, Bio-Rad Laboratories, Carl Roth, Bernd Kraft, or PanReac AppliChem, unless specified otherwise. Plasmid and PCR-product purification reagents, and DNA-purification columns were from Qiagen, Epoch Life Science, or Carl Roth.

Molecular cloning and plasmid preparation for recombinant protein production

We amplified DNA coding-regions for Ame1, Ctf19*,* Mcm21*,* Nkp1*,* Nkp2*,* or Okp1 by polymerase chain-reaction (PCR) from isolated *Kluyveromyces lactis* genomic DNA or isolated *Saccharomyces cerevisiae* genomic DNA, and prepared polycistronic constructs for Ame1-Okp1 variants, Ctf19-Mcm21-Okp1 variants, COMA variants, or Nkp1-Nkp2 as previously described (Schmitzberger & Harrison, 2012). We inserted PCR products into a ligation-independent cloning compatible pET3aTR expression plasmid, or into a similar plasmid encoding an N-terminal polyhistidine tag (*His*; with sequence MKSSHHHHHHENLYFQSNA), with a tobacco-etch virus (TEV) protease cleavage-site. We prepared polycistronic plasmids encoding *K. lactis* COMA variants with Ame1 variants or Okp1 variants, with the order (relative to T7 DNA-polymerase promoter) of truncated coding regions *His-Mcm21*$_{D\text{-}RWD}$ (encoding the Mcm21 D-RWD domain; residues 108–293; with an N-terminal polyhistidine tag)*, Ctf19*$_{D\text{-}RWD}$ (encoding the Ctf19 D-RWD domain; residues 107–270), *Okp1*, *Ame1*; Ctf19$_{\text{D-RWD}}$-Mcm21$_{\text{D-RWD}}$-Okp1 variants with the order of truncated coding regions *His-Mcm21*$_{108–293}$*, Ctf19*$_{107–270}$, *Okp1*; and Ame1-Okp1 variants with the order *His-Okp1*, *Ame1*, by overlapping PCR as described (Schmitzberger & Harrison, 2012). We prepared a dicistron that encodes *K. lactis* Nkp1-Nkp2 or *S. cerevisiae* Nkp1-Nkp2, with the coding-region order *His-Nkp1*, *Nkp2*, and a polycistron encoding *K. lactis* Nkp1-Nkp2 variants with truncations of Nkp1 or Nkp2 analogously. We carried out site-directed mutagenesis of *Okp1* in our *K. lactis* COMA-encoding polycistron to generate *Okp1_cmΔ* with *PfuTurbo* DNA Polymerase AD (Agilent Technologies) and deoxynucleotide mix from QuikChange Multi Site-directed Mutagenesis Kit (Thermo Fisher Scientific). We used restriction enzymes from New England BioLabs or Fermentas/Thermo Fisher Scientific; and T4 DNA Polymerase (LIC-qualified), for ligation-independent cloning, from Novagen/Merck Millipore.

Coding regions for COMA proteins from *S. cerevisiae* were inserted in plasmid pST39 (Tan, 2001) with the order of coding regions *Mcm21*, *Ctf19*, *Ame1*, and *Okp1*; and encoding a polyhistidine tag with a precision-protease cleavage site that is N-terminal of Ame1 (with sequence MGSSHHHHHHSLEVLFQGPH). The polycistron for production of *S. cerevisiae* Ame1-Okp1 was as described (Hornung et al, 2014); this construct encodes Okp1 followed by Ame1 with a C-terminal polyhistidine tag (HHHHHH).

For construct details and plasmids, see **Table EV5**. Our plasmids and PCR primer-sequences are available upon request.

Small-scale coding-region expression and Ni^{2+}-affinity recombinant protein purification in 96-well plates

We transformed plasmids into *Escherichia coli* BL21 Rosetta 2(DE3)pLysS cells (Novagen/ Merck Millipore). We grew *E. coli* cultures in 1.4 ml of terrific broth (Affymetrix/Thermo Fisher Scientific) (with ~ 0.5 % (v/v) glycerol) supplemented with

50 µg ml^{-1} carbenicillin and 35 µg ml^{-1} chloramphenicol, in 2 ml well-volume 96 deep-well plates that we sealed with oxygen-permeable AirPore tape sheets (Qiagen), at 37 °C, shaking at 400–800 rpm, until an absorbance at 600 nm (A_{600}) of the culture of 1–1.5. We induced recombinant protein production by addition of isopropyl-β-D-thiogalactopyranoside (IPTG; Anatrace or Thermo Fisher Scientific), to a final concentration of 0.2–0.4 mM or 0.8 mM, and grew cells for ~ 16 hrs, shaking at 400–800 rpm, at 18 °C. We harvested cells by centrifugation at 2000 rpm in a J6 centrifuge (Beckman Coulter) or at 4000 rpm in an Allegra X-14R centrifuge (Beckman Coulter), for 1800 sec, at 4 °C. We decanted the supernatant, and vortexed the plate for 60–180 sec. For chemical lysis, we resuspended cell pellets in the 96 deep-well plate, in 240 µl of a buffer of 50 mM Na-phosphate pH 8.0, 500 mM NaCl, 10 % (v/v) glycerol (buffer A), with 1 mM tris(2-carboxyethyl)phosphine (TCEP), 10 mM imidazole, 80 µg ml^{-1} lysozyme, 10 Units ml^{-1} recombinant DNase I (Roche Diagnostics) and protease inhibitors (2 µg ml^{-1} aprotinin (Roche Diagnostics), 0.5 µg ml^{-1} leupeptin (Roche Diagnostics), 0.7 µg ml^{-1} pepstatin (Roche Diagnostics), ~ 1 mM phenylmethanesulfonyl fluorid (PMSF; Sigma-Aldrich)) (lysis buffer) with 1.6 % (v/v) 10× Bugbuster (Novagen) that we had chilled on ice (ice-cold). We freeze-thawed lysates two or three times by submerging the deep-well plate first in liquid N_2 and subsequently transferring it to a lukewarm water bath for 300–600 sec. The plate was shaken at ~ 100 rpm for 600–1200 sec at 4 °C. We separated cell debris by centrifugation at 4000 rpm at 4 °C for 1800–2700 sec. We transferred 50 µl of Ni^{2+}-nitrilotriacetic agarose (NTA; Qiagen) slurry or His60 Ni Superflow resin (Clontech) slurry, which we had washed with ice-cold lysis buffer with 1.6 % (v/v) 10× Bugbuster, per well to a 96-well filter-plate (MultiScreenHTS BV plate, 1.2 µm; Merck Millipore). We transferred the soluble lysate fraction onto equilibrated Ni^{2+}-NTA or His60 Ni Superflow slurry, in the filter-plate. The filter-plate was shaken at ~ 100 rpm for 1200–1800 sec at 4 °C. We fixated the filter-plate over a 96-well plate (0.5 ml volume; USA Scientific), and centrifuged it at 1000–2000 rpm for 300–600 sec, at 4 °C. We washed (centrifugation at 1000–2000 rpm for 300–600 sec) the slurry with immobilized polyhistidine-tagged proteins three times, in the filter-plate, with 240 µl of ice-cold buffer A with 25 mM imidazole and 1 mM TCEP, at 4 °C. We eluted proteins, into a PCR plate (with ~ 200 µl well volume), with 40 µl of ice-cold buffer A with 500 mM imidazole, 0.5 mM TCEP, 2 µg ml^{-1} aprotinin, 0.5 µg ml^{-1} leupeptin, 0.7 µg ml^{-1} pepstatin, ~ 1 mM PMSF, and centrifugation of the plate at 2500 rpm for 300–600 sec at 4 °C. We combined, in PCR tubes, 12–15 µl of eluate with 5 µl of 2× sodium dodecyl sulfate (SDS) sample-buffer (Laemmli; Bio-Rad Laboratories)—supplemented with 715 mM β-mercapto-ethanol, and denatured samples for ~ 600 sec at 95–100 °C. We analysed samples by SDS polyacrylamid gel-electrophoresis (SDS-PAGE). We loaded 15 µl sample per well on 26-well Criterion TGX gels (Bio-Rad Laboratories) (see **Fig 4B**,**C**).

We stained our SDS-PAGE gels with a solution containing 0.06 % (w/v) Coomassie Brilliant Blue R. If we stained gels with silver stain, we used Silver Stain Plus Kit (Bio-Rad Laboratories).

Large-scale recombinant protein production, protein purification, and protein-assembly reconstitution

We produced most of our recombinant protein samples in *E. coli* BL21 Rosetta 2(DE3)pLysS, in terrific broth (Affymetrix or Merck Millipore) cultures (1–8 ltrs) and lysed them, essentially as previously described (Schmitzberger & Harrison, 2012), in

ice-cold lysis buffer (for recipe see above) with 0.5 mM or 1 mM TCEP (Sigma-Aldrich or Thermo Fisher Scientific) by sonication. Most of our protein purifications were similar as those described before for *K. lactis* COMA (Schmitzberger & Harrison, 2012). We purified our proteins at 4 °C. After lysis and removal of cell debris by centrifugation, we passed the soluble lysate fraction over a column with ~ 4–5 ml of immobilized Ni^{2+}-NTA or His60 Ni Superflow resin, by gravity flow and/or with a peristaltic pump. We washed the column with ~ 8–10 bead volumes (BVs) of ice-cold buffer A (see above) with 20 or 25 mM imidazole and 0.5–1.5 mM TCEP followed by 2–4 BVs of buffer A with 40 mM imidazole and 0.5–1.5 mM TCEP. We eluted proteins with 4–6 BVs of buffer A with 500 mM imidazole and 0.5–1.5 mM TCEP with 2 µg ml^{-1} aprotinin, 1 µg ml^{-1} pepstatin, 1 mM PMSF (Sigma-Aldrich), with 0.5 µg ml^{-1} leupeptin or without leupeptin. For most of our purifications (except for samples that we used for Ni^{2+}-affinity binding studies or for some mass-spectrometry experiments (see below)), we combined our eluate with TEV protease (produced from a pRK792 plasmid (Kapust et al, 2001); and purified with Ni^{2+}-affinity chromatography, and ion-exchange chromatography with HiTrap SP HP (GE Healthcare Life Sciences)) in an estimated 15:1–20:1 molar ratio (protein assembly:TEV protease), and dialyzed in Spectra/Por 2 dialysis tubing (12–14 kDa molecular weight cut off (MWCO), Spectrum labs) against 2 ltrs of 40 mM 4-(2-hydroxyethyl)-1-piperazineethanesulfonic acid (Hepes) pH 7.5 or pH 8–8.1, 500 mM NaCl, 10 % (v/v) glycerol, 1–4 mM TCEP for ~ 16–20 hrs at 4 °C. TEV-protease cleavage leaves an N-terminal serine-threonine-alanine residual on the previously polyhistidine-tagged protein. We passed TEV protease cleaved samples over a column with ~ 4–5 ml of Ni^{2+}-NTA or His60 Ni Superflow resin, collected the flowthrough and concentrated it (Amicon Ultra-15 concentrators with 10, 30, 50 or 100 kDa MWCO; Merck Millipore). We loaded the concentrate, after we had diluted it with a buffer of 38–40 mM Hepes pH 7.5 or 8.0, 100 mM NaCl, 10 % (v/v) glycerol, 0.5 mM TCEP, on a 5 ml anion-exchange chromatography column (HiTrap Q HP; GE Healthcare Life Sciences). Most of our protein samples, we eluted with a buffer of 30–40 mM Hepes pH 7.5 or pH 8.0, 10 % (v/v) glycerol, 0.5 mM TCEP and a 100–1000 mM NaCl gradient. For purifications of *K. lactis* COMA or *K. lactis* COMA-Nkp1-Nkp2, we included a plateau at 180 mM NaCl for five column volumes in the NaCl gradient. We concentrated elution fractions that contained our proteins of interest (with Amicon Ultra-15 concentrators), filtered them with Ultrafree-MC GV centrifugal filters (PVDF membrane, 0.22 µm; Merck Millipore), and loaded samples on a size-exclusion chromatography (SEC) column (Superdex 200 HiLoad 16/600 prep grade column; GE Healthcare Life Sciences). We eluted with a buffer of 23–25 mM Hepes pH 7.5, 300 mM NaCl, 5 % (v/v) glycerol, 0.5 mM TCEP, unless stated otherwise. For SDS-PAGE analyses, we generally used 4–20 % (w/v) acrylamide mini-PROTEAN TGX gels (Bio-Rad Laboratories) with a tris(hydroxymethyl)aminomethane (Tris)-glycine SDS buffer pH 8.3, or 10 % (w/v) acrylamide Bis-tris NuPAGE gels (Thermo Fisher Scientific) with a 2-(N-morpholino)ethanesulfonic acid (MES) SDS buffer pH 7.3 (Thermo Fisher Scientific). We concentrated protein samples with Amicon Ultra-4 or Vivaspin 20 concentrators (Sartorius stedim) by centrifugation. We estimated protein concentrations with the protein-sequence based theoretical extinction coefficients at 280 nm (Gasteiger et al, 2005), usually from absorbance measurements on a Nanodrop 1000 UV/VIS spectrophotometer (Thermo Fisher Scientific).

We purified *K. lactis* Nkp1-Nkp2 (**Appendix Fig S1A**) and our Nkp1-Nkp2 truncation variant (**Appendix Fig S5F**) as we describe above. We produced Nkp1 alone from our *Nkp1-Nkp2* polycistron—Nkp1 was present in excess in our purifications. For Nkp1 and Nkp1-Nkp2 samples that we used for deuterium-exchange that we show in **Appendix Fig S5E**, lysis buffer was with cOmplete, EDTA-free Protease Inhibitors Cocktail tablets (one tablet for 50 ml buffer; Roche Diagnostics) and ~ 1 mM PMSF instead of the general recipe that we describe above. We separated Nkp1 from Nkp1-Nkp2 on a HiTrap Q HP column (Nkp1 eluted in the flow-through), and purified it as in our general purification procedure that we describe above.

We reconstituted *K. lactis* COMA-Nkp1-Nkp2 (**Fig 1A**), by combining a TEV protease cleaved and Ni^{2+}-affinity chromatography purified COMA sample and a TEV protease cleaved Nkp1-Nkp2 sample that we had purified with Ni^{2+}-affinity chromatography and anion-exchange chromatography (and stored at −80 °C). We pooled samples of COMA and Nkp1-Nkp2, and carried out anion-exchange chromatography, as we describe in our general procedure above. We subsequently pooled COMA-Nkp1-Nkp2-containing fractions, concentrated them with a 100 kDa MWCO Amicon-15 concentrator, and injected 2 ml on SEC column (Superdex 200 HiLoad 16/600 prep grade); see **Fig 1A**.

We purified $Ame1_{1–260}$-$Okp1_{123–336}$ that we used for our deuterium-exchange experiments (**Fig 6A**; **Appendix Fig S6A**,**C**) or our $Ame1_{1–260}$-$Okp1_{108–308}$ variant (**Appendix Fig S4B**) as in our general procedure that we describe above, with the following modification. For SEC, we used a buffer with 25 mM Hepes pH 7.5, 500 mM NaCl, 5 % (v/v) glycerol, 0.5 mM TCEP. Using a higher NaCl concentration, than in our general purification procedure, reduced aggregation.

We purified samples of $Ctf19_{107–270}$-$Mcm21_{108–293}$-$Okp1_{108–336}$ and $Ctf19_{107–270}$-$Mcm21_{108–293}$-$Okp1_{123–336}$ that we show in **Fig 4D**, as in our general procedure that we describe above, with the following modifications. We pooled the flow-through fraction from chromatography on a HiTrap Q HP column, and subsequently purified it with cation-exchange chromatography on a 5 ml HiTrap SP HP column, with a buffer of 30 mM Hepes pH 8.1, 100 mM NaCl, 10 % (v/v) glycerol, 0.5 mM TCEP and a 100–1000 mM NaCl gradient.

We purified our samples $Ctf19_{107–270}$-$Mcm21_{108–293}$-$Okp1_{229–336}$ and $Ctf19_{107–270}$-$Mcm21_{108–293}$-$Okp1_{229–383}$ (**Fig 5A**,**B**), and $Ctf19_{107–270}$-$Mcm21_{108–293}$-$Okp1_{229–336}$ that we used for our deuterium-exchange experiments (**Fig 6A**; **Appendix Fig S6C**) as in our general procedure that we describe above, with the following modification. We purified by SEC with a buffer of 24 mM Hepes pH 7.5, 150 mM NaCl, 5 % (v/v) glycerol, 0.5 mM TCEP.

We purified our sample $Ctf19_{107–270}$-$Mcm21_{108–293}$-$Okp1_{295–360}$ (**Appendix Fig S7A**) as in our general procedure that we describe above, with the following modification. We carried out SEC with a buffer of 25 mM Hepes pH 7.5, 150 mM NaCl, 5 % (v/v) glycerol, 0.5 mM TCEP.

We purified full-length Ctf19-Mcm21 that we used for our deuterium-exchange experiments (**Fig 6B**; **Appendix Fig S6D**,**E**) as previously described (Schmitzberger & Harrison, 2012).

K. lactis MIND core (MIND-C1; with Dsn1 residues 230–479, Mtw1 residues 1–233, full-length Nnf1, full-length Nsl1) was reconstituted and purified as previously described (Dimitrova et al, 2016), in a buffer of 30 mM HEPES, pH 7.5, 200 mM NaCl, 1 mM TCEP. In the same buffer, we reconstituted *K. lactis* COMA-MIND, for our mass-spectrometry experiments that we show in **Fig EV1A**, by combining

purified COMA and purified MIND-C1 samples, which we had stored on ice after purification, and eluting from a SEC column (Superdex 200 10/300; GE Healthcare Life Sciences).

We affinity-purified *K. lactis* COMA that lacks the Ctf19-Mcm21 binding motif (Okp1_cmΔ; **Appendix Fig S8A**) with lysis buffer with cOmplete, EDTA-free Protease Inhibitor Cocktail tablets (one tablet for 50 ml buffer; Roche Diagnostics) and 1 mM PMSF (Sigma-Aldrich), instead of the protease inhibitors in our general procedure that we describe above.

For Ni^{2+}-affinity-based binding assays (see below), we did not cleave the polyhistidine tag of our purified proteins, and proceeded directly from Ni^{2+}-affinity chromatography to anion-exchange chromatography. We purified recombinant *S. cerevisiae* COMA according to our general procedure that we describe above, but without TEV-protease cleavage and without 2nd Ni^{2+}-affinity chromatography. *S. cerevisiae* Chl4-Iml3 was prepared as previously described (Hinshaw & Harrison, 2013).

We produced *S. cerevisiae* Okp1-Ame1-His in lysogeny broth (LB) medium. We purified recombinant *S. cerevisiae* Okp1-Ame1-His or *S. cerevisiae* His-Nkp1-Nkp2, for our binding experiments (see **Fig EV3B**), similar to our general protein-purification protocol that we describe above with some modifications. For purification of *S. cerevisiae* Okp1-Ame1-His or *S. cerevisiae* His-Nkp1-Nkp2, we resuspended the cell pellet in a buffer of 20 mM Na-phosphate pH 7.5, 500 mM NaCl, 20 mM imidazole, or in a similar lysis buffer as previously described (Hornung et al, 2014). After sonication and centrifugation, we incubated our cleared lysates with Ni^{2+}-NTA, washed Ni^{2+}-NTA with a buffer of 20 mM Na-phosphate pH 7.5, 500 mM NaCl, 20 mM imidazole; and eluted with a buffer of 20 mM Na-phosphate pH 7.5, 500 mM NaCl, 200 mM imidazole. We purified pooled Ni^{2+}-NTA eluates of *S. cerevisiae* Okp1-Ame1-His or *S. cerevisiae* His-Nkp1-Nkp2 with a Superdex 200 10/300 column on an Äkta FPLC system, and with a buffer of 20 mM Na-phosphate pH 6.8, 200 mM NaCl, 2.5 % (v/v) glycerol, 0.5 mM TCEP. We concentrated eluted protein fractions, flash froze the concentrate in liquid N_2, and stored it at −80 °C, until usage.

We stained our SDS-PAGE gels with a solution containing 0.06 % (w/v) Coomassie Brilliant Blue R.

Analytical size-exclusion chromatography

We carried out analytical SEC for samples that we show in **Fig 5A**,**B** and **Fig EV3A** on a Superdex 200 10/300 column on an Äkta FPLC (GE Healthcare Life Sciences) system, at 4 °C. For SEC that we show chromatograms of in **Fig 5A**,**B**, we used an Nkp1-Nkp2 sample that after purification we had stored at −80 °C; and samples of Ctf19-Mcm21-Okp1 variants that after purification we had stored on ice. We loaded ~ 4 nanomole of each protein assembly in a volume of ~ 500 μl. We incubated combinations of protein samples on ice for ~ 3000 sec, before injecting on the column. For this experiment and most of our other analytical SEC experiments, we eluted with a buffer of 24 mM or 25 mM Hepes pH 7.5, 300 mM NaCl, 5 % (v/v) glycerol, 0.5 mM TCEP. We incubated combinations of samples of different protein assemblies for the chromatograms that we show in **Fig EV3A** on ice for ~ 3600 sec, before injecting the combined sample on the column. For the SEC that we show chromatograms of in **Fig EV3A**, we used a sample of full-length Ctf19-Mcm21 that after purification we had stored at −80 °C, and an Nkp1-Nkp2 sample that after purification we had stored on ice. We injected 12–25 nanomole of our protein

samples in a volume of ~ 100 μl. For the SEC that we show in **Appendix Fig S7B**, we used a Superdex 200 HiLoad 10/600 prep grade column, and we used protein samples that we had stored on ice for a few days after purification. We injected samples in a volume of 2 ml. We combined 159 nanomol of Ctf19$_{D-RWD}$-Mcm21$_{D-RWD}$-Okp1$_{295–383}$ sample with 123 nanomole Nkp1-Nkp2 sample (in a volume of ~ 1 ml), and incubated this mixture for ~ 600 sec on ice before loading it on the column. Ctf19$_{D-RWD}$-Mcm21$_{D-RWD}$-Okp1$_{295–383}$ co-eluted with Nkp1-Nkp2 (**Appendix Fig S7B**); in a buffer of 25 mM Hepes pH 7.5, 300 mM NaCl, 5 % (v/v) glycerol, 0.5 mM TCEP, but dissociated from Nkp1-Nkp2 after cryo-freezing in liquid N_2 and storage at −80 °C. For our sample analysis that we show in **Fig EV3B**, we carried out SEC with a Superdex 200 PC 3.2/300 column (GE Healthcare Life Sciences) on an Ettan LC system (GE Healthcare Life Sciences), with a buffer of 20 mM Na-phosphate pH 6.8, 200 mM NaCl, 2.5 % (v/v) glycerol, 0.5 mM TCEP; we incubated combinations of protein samples on ice for ~ 3000 sec, before we injected them on the column; we injected protein samples at a concentration of ~ 5 μM in a volume of 50 μl (we estimated concentrations with Bradford spectrophotometric assay, with Protein Assay Dye Reagent Concentrate (Bio-Rad Laboratories), with bovine serum albumin as standard).

Limited proteolysis experiments, and mass spectrometry of resultant protein fragments or proteins

We used purified protein samples, which we had stored on ice after purification, for our limited proteolysis experiments with trypsin (Sigma-Aldrich) or elastase (Worthington Biochemical Corporation).

We incubated ~12–60 nanomole of COMA-Nkp1-Nkp2 (**Fig EV4A**; **Table EV1**) in a 10:1 molar ratio with trypsin for 180 sec, 240 sec, or 600 sec, or in a 10:1 molar ratio with elastase for 180 sec, in a buffer of 25 mM Hepes pH 7.5, 300 mM NaCl, 5 % (v/v) glycerol, 0.5 mM TCEP, at 22–25 °C. We incubated ~ 25 nanomole of Ame1-Ctf19$_{D-RWD}$-Mcm21$_{D-RWD}$-Okp1 (**Fig EV4B**) in a 10:1 molar ratio with trypsin for 255 sec at 22–25 °C. We stopped our proteolysis reactions by adding 4-(2-aminoethyl)benzenesulfonyl fluoride hydrochloride (AEBSF; Sigma-Aldrich) solution to a concentration of ~ 1.5 mM, and gel-filtered samples on a Superdex 200 HiLoad 16/600 prep grade column, with a buffer of 25 mM Hepes pH 7.5, 300 mM NaCl, 5 % (v/v) glycerol, 0.5 mM TCEP. We pooled fractions corresponding to the principal peak from SEC, concentrated them (Amicon concentrators), and denatured samples by addition of solid high-grade guanidine hydrochloride (cat. no 50933, Sigma-Aldrich) until saturation of the solution.

For limited proteolysis without subsequent SEC, we incubated ~ 910 picomole of COMA-Nkp1-Nkp2 (**Table EV2**) in a molar ratio of 10:1 with elastase or trypsin, in a buffer of 25 mM Hepes pH 7.5, 300 mM NaCl, 5 % (v/v) glycerol, 1.5 mM L-methionine for 180 sec or 600 sec, at 22–25 °C. In a similar buffer with 2.5 mM L-methionine, we incubated ~ 5.8 nanomole of *K. lactis* Nkp1-Nkp2 in a molar ratio of 10:1 with elastase or trypsin, for 300 sec or 600 sec, at 22–25 °C (**Table EV3**). We stopped our limited proteolysis reactions by addition of solid high-grade guanidine hydrochloride until saturation of the solution.

David S. King analysed protein fragments with mass spectrometry by electrospray ionization with Fourier-transform ion resonance-cyclotron or ion-trap mass spectrometers at the Howard Hughes Medical Institute mass spectrometry facility (University of California, Berkeley); and analysed mass spectra. He analogously analysed our purified Ctf19$_{107–270}$-Mcm21$_{108–293}$-Okp1 variant samples

(**Figs 4D**;**5A**,**B**), or other protein samples, such as Nkp1-Nkp2, which we had not actively proteolysed.

Binding assays with *in vitro* translated proteins

Coding regions, as PCR products or in plasmids, for proteins for *in vitro* translation with a Kozak translation-initiation sequence were prepared as previously described (Hinshaw & Harrison, 2013). We produced S^{35}-labeled proteins, either from PCR products (for experiments with *K. lactis* COMA) or, for experiments with *S. cerevisiae* COMA, from pET3aTR plasmids, by *in vitro* translation in rabbit-reticulocyte lysate (TnT lysate systems; Promega Corporation) with S^{35}-labeled L-methionine, following the manufacturer's instructions. For each binding experiment, we incubated S^{35}-labeled proteins with 10 µg or 15 µg of purified *K. lactis* COMA (**Fig EV6A**), which after purification we had stored on ice or at −80 °C, in a buffer of 15 mM Hepes pH 7.5, 110 mM NaCl, 10 mM imidazole, 2.5 % (v/v) glycerol, 0.05 % (v/v) octyl phenoxypolyethoxylethanol (Nonidet P-40), 0.5 mM TCEP (IVT buffer) for 3600 sec on ice. After addition of 30 µl of Ni^{2+}-NTA slurry (equilibrated in IVT buffer) to this mixture, we incubated under rotation for 1800 sec at 4 °C. We used Ni^{2+}-NTA purified polyhistidine-tagged maltose binding protein, which we had stored at −80 °C after purification, as a negative control; and 10 % (v/v) of the reaction mixture (from sample for maltose-binding protein) as positive control. After washing Ni^{2+}-NTA resin three times with 500 µl of IVT buffer, we eluted proteins by addition of 25 µl of SDS sample-buffer with 500 mM imidazole, and denatured eluted proteins by heating for 10 min at 100 °C**.** We separated denatured proteins on 10–20 % (w/v) acrylamide Criterion Tris-HCl or TGX gels (Bio-Rad Laboratories). After drying gels, we transferred signals to a phosphor-imaging plate, and recorded phosphorescence signals on a personal molecular imager (Bio-Rad Laboratories). For binding experiments with *S. cerevisiae* COMA (**Fig EV6B**), our protocol was similar. For each binding experiment, we used 15 µg of recombinant purified *S. cerevisiae* COMA, which we had stored at −80 °C after purification; our IVT buffer was with 20 mM Hepes pH 7.5, 150 mM NaCl, 20 mM imidazole, 5 % (v/v) glycerol, 0.05 % (v/v) Nonidet P-40, 0.5 mM TCEP.

Nanoflow electrospray-ionization mass spectrometry of protein assemblies

For our nanoflow electrospray-ionization mass-spectrometry (for review see (Sharon & Robinson, 2007)), we used purified protein samples that we had flash-frozen in liquid N_2, and had stored at −80 °C and on solid CO_2. We transferred proteins to a buffer of 200 mM ammonium acetate pH 6.7–7.3, with Amicon concentrators. We sprayed samples usually at a concentration of 2–10 µM, unless specified otherwise. We determined concentrations with a PicoDrop UV/VIS spectrophotometer. We acquired mass spectra or tandem mass-spectra (Benesch et al, 2006), in positive ion mode, on a high mass quadruple time-of-flight (Q-TOF)-type instrument (Sobott et al, 2002) adapted for a QSTAR XL platform (MDS Sciex) (Chernushevich & Thomson, 2004). To spray samples, we used in-house prepared gold-coated glass capillaries (Nettleton et al, 1998). Optimized instrument parameters were as follows: ion-spray voltage 1300 Volt, declustering potential 100 Volt, focusing potential 200 Volt and collision energy up to 200 Volt, MCP 2350 Volt. In tandem mass-spectrometry, we selected the relevant $m\ z^{-1}$ range in the second quadrupole. The proteins in that range were subjected to acceleration in the collision cell. We used argon as a collision gas at maximum pressure. We calibrated all spectra externally using a cesium-iodide solution (100 mg ml^{-1}). We derived mean values and standard

deviations for masses (see inset tables in our figures) with the MassLynx software (Waters Corporation), selecting masses from a series of identified m z^{-1} peaks in our spectrum. We carried out partial, in-solution disruption of purified *K. lactis* COMA (**Fig 2A**,**B**; **Appendix Fig S2A**) by adding acetic acid to a concentration of 5 % (v/v) in 100 mM ammonium acetate, to a pH 4.0. After addition of acetic acid, we incubated this mixture on ice for 900 sec, before spraying the solution in the mass spectrometer. For our analysis of Ctf19-Mcm21-Okp1 that we show in **Appendix Fig S2B**, we analogously incubated COMA with 100 mM ammonium acetate pH 3.7, 5 % (v/v) acetic acid. For our analyses of purified samples of COMA (**Figs 1C**;**EV1B**) or COMA-MIND (**Fig EV1A**) we sprayed in 200 mM ammonium acetate pH 6.7–7.3. We sprayed COMA-Nkp1-Nkp2 (**Figs 1B**;**EV2A**,**B**) in 200 mM ammonium acetate pH 7.4.

Hydrogen-deuterium exchange of proteins followed by mass spectrometry of peptides

For our deuterium-exchange experiments, we used purified protein samples that we had flash-frozen in liquid N_2, and had stored −80 °C and on solid CO_2. In the following, we describe experiments and analyses of proteins in COMA and COMA-Nkp1-Nkp2. The procedure for our other samples was similar, unless stated otherwise.

We constructed a reference list of pepsin proteolysed *K. lactis* COMA-Nkp1-Nkp2 peptide masses using a non-deuterated sample as follows. We diluted 5 µl aliquot of protein stock (1.5–3 mg ml^{-1} (estimated from absorbance at 280 nm) in 24 mM Hepes pH 7.5, 300 mM NaCl, 5 % (v/v) glycerol, 0.5 mM TCEP) in a 1:10 ratio, by adding 45 µl of a buffer with 20 mM Tris, pH 8.0, 150 mM NaCl (H_2O Reaction buffer). We acidified this sample by mixing with 10 µl of 2 M glycine pH 2.5 (H_2O Stop Buffer). The sample was digested online using an immobilized pepsin column (Poroszyme, Applied Biosystems, Thermo Fisher Scientific) with 0.07 % (v/v) formic acid in water as mobile phase (flow rate 3.33 µl sec^{-1}). Digested peptides were passed over a C18 trapping column (ACQUITY BEH C18 VanGuard Pre-column, Waters Corporation), and subsequently over a reversed-phase chromatography column (ACQUITY UPLC BEH C18 column, Waters Corporation) with a 6–40 % (v/v) gradient of acetonitrile in 0.1 % (v/v) formic acid at 0.66 µl sec^{-1} using the nanoACQUITY Binary Solvent Manager. Total time of a single run was 810 sec. All fluidics, valves, and columns were maintained at 0.5 °C, using the HDX Manager (Waters Corporation). The pepsin column was kept at 13 °C inside the temperature-controlled digestion compartment of the HDX manager. The C18 VanGuard Pre-column outlet was coupled directly to the ion source of a SYNAPT G2 HDMS mass spectrometer (Waters Corporation). For our experiments comparing assemblies COMA and COMA-Nkp1-Nkp2 (**Fig 3A**,**B**; **Appendix Fig S3A**,**B**,**C**,**E**), the mass spectrometer was working in ion-mobility mode (Pringle et al, 2007). For our other deuterium-exchange experiments, we did not use ion-mobility mode. Leucine-enkephalin solution (Sigma-Aldrich) was used as a lock mass. For protein identification, mass spectra were acquired in MSE mode over the m z^{-1} range of 50–2000. We used the following spectrometer parameters; electrospray-ionization: positive mode, capillary voltage: 3 kV, sampling cone voltage: 35 V, extraction cone voltage: 3 V, source temperature: 80 °C, desolvation temperature: 175 °C, desolvation-gas flow: 222 ml sec^{-1}. Peptides were identified using ProteinLynx Global Server software (Waters Corporation). We input the experimental data of

identified peptides that included mass to charge (m z^{-1}), charge, retention time, and ion-mobility drift time in the DynamX software program (version 2.0; Waters Corporation).

We carried out hydrogen-deuterium exchange experiments essentially as described (Kupniewska-Kozak et al, 2010) for our non-deuterated samples, with our Reaction buffer containing D_2O (99.8 % (v/v); Armar Chemicals); we adjusted pH_{read} (uncorrected meter reading) using DCl or NaOD (Sigma-Aldrich). After mixing 5 µl protein stock with 45 µl D_2O Reaction buffer, the exchange reactions proceeded for 10 s, 60 sec, or 1200 sec, at 22–25 °C. We quenched deuterium-exchange reactions by reducing the pH_{read} to 2.5, through adding the reaction mixture into a microcentrifuge tube that contained Stop buffer (2 M glycine pH_{read} 2.5), which we had cooled on ice. Immediately after quenching, we manually injected the sample into the nanoACQUITY (Waters Corporation) Ultra Performance Liquid Chromatography (UPLC) system. We carried out pepsin digestion, liquid chromatography, and mass-spectrometry analyses as described for our non-deuterated samples. We carried out two kind of control experiments to determine experimental in-exchange and back-exchange values, as previously described (Kupniewska-Kozak et al, 2010). Briefly summarized, to determine the minimum exchange of peptides—our in-exchange control, we added D_2O reaction buffer to Stop buffer cooled on ice. We added this mixture to protein stock and immediately pepsin digested it, before we carried out liquid chromatography coupled to mass-spectrometry analysis, as we describe above. We calculated the deuteration level of peptides from our in-exchange control experiment using DynamX. We used values from this analysis as the minimum exchange (M_{ex}^{0}; see next paragraph). For our determination of back-exchange peptide values, we combined 5 µl protein stock with 45 µl of D_2O Reaction buffer, incubated this mixture ~ 15–19 hrs at 4 °C, before we combined it with Stop buffer, and analyzed it analogously as our in-exchange control. We used determined peptide deuteration-values from our back-exchange experiment as the maximum deuterium exchange (M_{ex}^{100}). For each one of our different (protein assembly) sample comparisons, we used respective uniform in-exchange and back-exchange controls. We repeated all deuterium-exchange and in-exchange and back-exchange control experiments at least three times.

We calculated peptide deuteration-levels with DynamX (version 2.0), using our pepsin-proteolysed peptide list obtained from the ProteinLynx Global Server. We selected peptides in DynamX with the following acceptance criteria: minimum intensity threshold 2000, minimum products per amino acids 0.2. We analysed isotopic envelopes from deuterium-exchange with DynamX with the following parameters: RT deviation ± 15 sec, m z^{-1} deviation ± 12.5 parts per million (ppm), drift time deviation ± 2 time bins (if applicable). We manually verified each isotopic envelope, which had been identified and assigned to peptides by the automated analysis with DynamX, to ensure valid calculation of the mean peptide masses from our deuterium-exchange experiment (M_{ex}) and from our in-exchange and back-exchange experiments (M_{ex}^{0} and M_{ex}^{100}). We discarded ambiguous or overlapping isotopic envelopes from further analyses. We exported our selected deuterium-exchange data to Excel (Microsoft Corporation), and calculated the hydrogen-deuterium exchange mass shifts and fraction of exchange. We calculated the fraction of peptide deuterium-exchange (*f*) with the formula:

$$f = \frac{M_{ex} - M_{ex}^{0}}{M_{ex}^{100} - M_{ex}^{0}}$$

We calculated mean values and standard deviations for the exchange fraction (*f*) from at least three independent experiments. Most of our standard deviations were below 2 % of our mean determined deuteration fractions. We visualized our data with a previously described Excel macro template file (Black et al, 2007) that plots deuterium-exchanged peptide representations that are coloured by fraction of deuterium exchange, underneath their corresponding position in a linear amino-acid sequence representation of the protein the respective peptides originate from.

Dynamic light scattering measurements and multi angle laser-light scattering measurements

For our light scattering measurements, we used protein samples that we had stored on ice after purification. We measured dynamic light-scattering data of *K. lactis* COMA at a concentration of 21 μM (assuming a COMA dimer), and of *K. lactis* Nkp1-Nkp2 or *K. lactis* COMA-Nkp1-Nkp2 at similar concentrations, after filtration through a 0.22 μm Ultrafree-MC GV filter, in a quartz cuvette on a Dynapro instrument (Wyatt Technology) with a laser of 826.2 nm, at 15 °C. Multiple monomodal auto-correlation function were fit in cumulant-expansion analysis mode for our data with the Dynamics software version 5.26.56 (Wyatt Technology) (see **Appendix Fig S2C**).

For multi angle laser-light scattering, we injected our protein samples onto a Superdex 200 10/300 SEC column (GE Healthcare Life Sciences) that was mounted on a high performance liquid-chromatography system (1260 Infinity LC; Agilent Technologies). For samples eluting from this column at a flowrate of 6.7 μl sec^{-1}, at 22–25 °C, we measured multi angle laser-light scattering data with a laser of 663.9 nm on a Dawn Heleos-II detector (Wyatt-846-H2; Wyatt Technology) and refractive indices with a laser of 658 nm with an Optilab T-rEX instrument (Wyatt-512-Trex; Wyatt Technology). Before injection on the column, we filtered protein samples with 0.22 μm Ultrafree-MC GV filters. For measurements of purified *K. lactis* COMA (**Fig 1D**), we injected 100 μl at ~ 43 μM, and eluted with a buffer of 30 mM Hepes pH 7.5, 200 mM NaCl, 1 mM TCEP, 0.4 % (v/v) NaN_3. Injecting lower amounts of COMA did not yield interpretable multi angle laser-light scattering signals. Our multi angle laser-light scattering data of COMA-Nkp1-Nkp2 under similar solution conditions were inconclusive, with respective to the sample's multimeric state (the calculated molar mass was between that for monomeric COMA-Nkp1-Nkp2 and dimeric COMA-Nkp1-Nkp2). For measurements of purified *K. lactis* Nkp1-Nkp2 (**Appendix Fig S1B**), our measurement conditions were similar to those for COMA. We injected 70 μl of sample at ~ 120–150 μM, and eluted with a buffer of 25 mM Hepes pH 7.5, 300 mM NaCl, 5 % (v/v) glycerol 0.5 mM TCEP, 0.4 % (v/v) NaN_3. We used bovine serum albumin for calibration (detector-signal normalization, peak broadening). We analysed our multi angle laser-light scattering data with the Astra software (version 6.1.5.22; Wyatt Technology), with protein concentrations determined from in-line refractive index measurements, and using a dn/dc value of 0.185 ml g^{-1} (a refractive index of 1.33 was chosen for the aqueous solution). We fit our light scattering data with a first order Zimm function with linear regression, as implemented in the Astra software.

Sedimentation-equilibrium analytical ultracentrifugation analyses

For our sedimentation-equilibrium ultracentrifugation analyses, we used protein samples that we had stored on ice after purification. We recorded sedimentation equilibrium analytical ultracentrifugation data with a ProteomeLab Optima XL-I

analytical ultracentrifuge (Beckman Coulter) and an An-60 Ti rotor (Beckman Coulter) equipped with a 12-mm wide Epon six-chamber double-sector sample cell. Our protein samples were in a buffer of 25 mM Hepes pH 7.5, 300 mM NaCl, 5 % (v/v) glycerol, 0.5 mM TCEP. We used three different protein concentrations corresponding to an absorbance at 280 nm of 0.25, 0.5, and 0.75, and estimated protein concentrations with the protein-sequence based theoretical extinction coefficients at 280 nm (Gasteiger et al, 2005). We centrifuged our *K. lactis* COMA sample sequentially at 8000, 10000, 15000, 23000 rotations per minute (rpm) at 4 °C (**Appendix Fig S1C**). We centrifuged our *K. lactis* Ctf19-Mcm21 sample sequentially at 9000, 12500, 15000, 18000, 22000 rpm at 4 °C (**Appendix Fig S1D**). To determine sedimentation, we measured the absorbance at 280 nm every four hours during centrifugation. We fit our Ctf19-Mcm21 data with a global fit (with data from three different concentrations), with a 'Single Species of Interacting System' model, without molecular mass conservation constraints in SEDPHAT. We fit our data of COMA with a global fit (with data from three different concentrations) with a 'Monomer-Dimer Self-Association' model, fixed the molecular mass to that corresponding to monomeric COMA, left molecular mass conservation-constraints out, and allowed the K_a to vary during the fit calculation in SEDPHAT. Our sedimentation-equilibrium analytical ultracentrifugation data are available upon request.

Protein crystallization

For our crystallization trials, we used protein samples that we had stored on ice after purification. We screened for crystallization with several commercially available 96-well based crystallization screens, including MIDAS (Grimm et al, 2010), Morpheus (Gorrec, 2009), PACT premier (Newman et al, 2005), PGA (Hu et al, 2008), and Proplex (Radaev et al, 2006) (all from Molecular Dimensions), in 400 nl hanging drops in 96-well sitting-drop iQ plates (TTP Labtech), with a Mosquito crystallization-robot (TTP Labtech). Crystals formed by vapour diffusion at 20 °C. We obtained crystals of a *K. lactis* COMA variant that contains the Ctf19-Mcm2 D-RWD domains and full-length Ame1-Okp1 (Ame1-Ctf19$_{107–270}$-Mcm21$_{108–293}$-Okp1; for chromatogram see Fig 1C in (Schmitzberger & Harrison, 2012)), which did not diffract to higher than 50 Å resolution (data not shown), with 300–500 mM K-tartrate. For our *K. lactis* Ctf19$_{107–270}$-Mcm21$_{108–293}$-Okp1$_{295–360}$ assembly, we obtained crystals, which diffracted to high resolution (**Table EV4**), under many different chemical conditions. We scaled up crystallization in EasyXtal 15-well tools crystallization plates with screw caps (Qiagen), by preparing 2 µl or 3 µl hanging drops with a 1:1, 2:1, or 1:2 ratio of protein solution to precipitant solution; using a protein solution at a concentration of 62–370 µM. Crystals with dimensions of 50 µm × 50 µm × 50 µm – 100 µm × 100 µm × 100 µm grew typically within 1–4 days, at 20 °C. Crystals that we obtained in conditions with 35 % (v/v) glycerol ethoxylate, 200 mM Li-citrate (condition G6, MIDAS screen) diffracted to highest resolution (~ 2 Å). We mounted crystals in nylon loops (cryoloops, Hampton Research) immobilized on magnetic bases (CrystalCap, Hampton Research). We cryo-cooled crystals in liquid N_2, directly from crystallization drops; or after we had added a solution of 45 % (v/v) glycerol ethoxylate, 300 mM Li-citrate, 10 % (v/v) glycerol, 1 mM TCEP (cryo-solution) to crystallization drops, and after we had subsequently transferred crystals to this cryo-solution.

X-ray diffraction data collection, crystal-structure determination, and refinement

From our crystals, cryo-cooled at −173.5 °C, we collected X-ray diffraction data images (2×2 binned) at beamline 24-ID-E of the Advanced Photon Source (Argonne National laboratory), with a charge-coupled device detector (Quantum 315; Area Detector Systems Corporation), and a microdiffractometer with 50 µm aperture. We indexed and integrated X-ray diffraction data with XDS (version January 2014) (Kabsch, 1993), and scaled our integrated data with Aimless (version 0.1.27) (Evans & Murshudov, 2013) of the CCP4 suite (Winn et al, 2011) keeping Friedel pairs separated. We used cTruncate (French & Wilson, 1978) for conversion of indexed intensities into structure-factor amplitudes. We assessed our data by evaluation of $CC_{1/2}$ (Karplus & Diederichs, 2012) (**Table EV4**). We determined our crystal structure in space group $P22_12_1$ by molecular replacement with the coordinates of the Ctf19-Mcm21 (PDB code: 3ZXU; (Schmitzberger & Harrison, 2012)) D-RWD modules ($Ctf19_{107–270}$-$Mcm21_{108–293}$) with phenix Phaser (version 2.5.6) (McCoy et al, 2007). Two assemblies are the asymmetric unit. We also determined structures, which originated from crystals from different crystallization conditions than the one that yielded our high resolution structure, to lower resolution (3–4 Å) than for the structure that we present in our manuscript. These structures are in space group I222 or space group $P4_32_12$, with one or two Ctf19-Mcm21-Okp1 assemblies as asymmetric unit, respectively. These structures are overall similar to our high resolution structure that we solved in space group $P22_12_1$. After molecular replacement, we used phenix.AutoBuild (Adams et al, 2010) for initial rebuilding and refinement of our structure. We subsequently refined against structure-factor amplitudes with phenix.refine (version 1.9) (Afonine et al, 2012), with a maximum likelihood target-function, with a test set of 2.4 % of randomly selected reflection indices, and two-fold non-crystallographic symmetry (NCS) torsion-angle restraints (*B* factors were not restrained); and manually rebuilt our model in σ_A-weighted (Read, 1986) electron-density maps with COOT (versions 0.7.2 and 0.8.7) (Emsley & Cowtan, 2004), until convergence of the R_{free}. Hydrogens were added in the riding positions throughout refinement. For the last refinement rounds we omitted NCS restraints. We show statistics for X-ray diffraction data-collection, refinement, and final protein model in **Table EV4**.

Isothermal titration calorimetry (ITC) measurements and microscale-thermophoresis (MST) measurements with Okp1-derived synthetic peptides and Ctf19-Mcm21

For our ITC measurements and MST measurements, we purified full-length *K. lactis* Ctf19-Mcm21 analogously to our general procedure that we describe above in the section ‘Large-scale recombinant protein production, protein purification, and protein-assembly reconstitution‘, with some modifications. Lysis buffer was with cOmplete, EDTA-free Protease Inhibitor Cocktail tablets (one tablet for 50 ml; Roche Diagnostics) and 1 mM PMSF, instead of the protease inhibitors that we describe in our section ‘Large-scale recombinant protein production, protein purification, and protein-assembly reconstitution‘. We concentrated Ctf19-Mcm21 samples with a 10 kDa MWCO Vivaspin 20 concentrator. For SEC, we used a buffer of 25 mM Hepes pH 7.5, 150 mM NaCl, 5 % (v/v) glycerol, 0.5 mM TCEP. For our ITC measurements or MST measurements, we used a sample of full-length Ctf19-Mcm21 that we had stored on ice after purification and/or labeling (see below).

Mathias Madalinski at the Protein Chemistry core-facility (Research Institute of Molecular Pathology (IMP), Vienna) synthesized our Okp1-derived peptides. For our MST experiments we usually used peptide solutions that we had prepared within a few hours before our measurements, or had stored on ice for one or two days. For concentration determination of Okp1-derived peptide solutions and Okp1-derived peptide-variant solutions, we labeled sample aliquots of peptide solutions in a buffer of 25 mM Hepes pH 7.5, 150 mM NaCl, 5 % (v/v) glycerol, 0.5 mM TCEP**,** ~ 0.0375 % (v/v) or 0.05 % (v/v) Tween 20 (Nanotemper Technologies) (ITC/MST buffer), with o-Phthaldialdehyde (fluoraldehyde (OPA) reagent solution; Thermo Fisher Scientific), according to the manufacturer's instructions. We measured fluorescence in a 96-well Clear Flat Bottom Polystyrene NBS assay plate (cat. no: 3651, Corning) on a Synergy H1 Hybrid Multi-Mode Microplate Reader (BioTek instruments), with excitation at 340 nm and emission at 425 nm, at 37 °C. For Okp1-derived peptide-concentration determinations, we used the fluorescence signal of known concentrations (from weighing out specific amounts) of a synthetic peptide with sequence 'KGKGK' to derive a non-linear standard function. For our Okp1-derived peptide variants that only have two lysines, rather than three lysines (see **Fig 6G**), we derived concentrations from the absorbance at 205 nm of Okp1-peptide solutions, in a buffer of 25 mM Hepes pH 7.5, 150 mM NaCl, 5 % (v/v) glycerol, 0.5 mM TCEP, at 205 nm in 0.2 mm glass cuvettes with a Chirascan Plus circular dichroism spectrometer (Applied Photophysics), at 22–25°C. From these absorbance measurements, we calculated peptide-sample concentrations, after subtracting the absorbance of our buffer of 25 mM Hepes pH 7.5, 150 mM NaCl, 5 % (v/v) glycerol, 0.5 mM TCEP, using peptide extinction coefficients calculated based on their amino-acid sequence with https://spin.niddk.nih.gov/clore/ (Anthis & Clore, 2013).

For ITC experiments (**Fig 6F**), we transferred our purified Ctf19-Mcm21 and Okp1-derived synthetic peptide to a buffer of 25 mM Hepes pH 7.5, 150 mM NaCl, 5 % (v/v) glycerol, 0.5 mM TCEP**,** 0.05 % (v/v) Tween 20. The presence of 0.05 % Tween 20 was important to prevent precipitation of Ctf19-Mcm21 in the calorimeter reaction-cell. Immediately prior to ITC measurements, we degassed solutions of *K. lactis* Okp1-derived synthetic peptide with sequence 'SNEIYQQDKIDFNLKLKTDF' (corresponding to residues 318–337; **Fig 6E**) and Ctf19-Mcm21 with a MicroCal ThermoVac degasser (GE Healthcare Life Sciences) for 300 sec at 20 °C. We carried out ITC measurements on a MicroCal VP-ITC calorimeter (GE Healthcare Life Sciences). We transferred 1.42 ml of Ctf19-Mcm21 solution at 13 µM to the calorimeter reaction-cell that was kept at 25 °C. Okp1-derived peptide at 120 µM was injected into the reaction cell; the first injection was with 5 µl (over 10 sec), followed by 29 injections, each lasting 20 sec, of 10 µl. Injections were repeated every 300 sec. The ITC reaction-cell was under continuous stirring at 307 rpm. The Wiseman (*c*) parameter for our measurements with Ctf19-Mcm21 was ~ 290. We did three independent measurements that yielded similar ITC data, from which we derived mean value and standard error of regression (**Fig 6F**). We subtracted from these data the reference signal of our Okp1-derived peptide titrated to ITC/MST buffer. Our data are consistent with a 1:1 binding stoichiometry of Okp1 peptide: Ctf19-Mcm21. We used Origin software (version 7.0552; OriginLab), to derive the K_d, by fitting a non-linear single set of sites binding-function with chi^2 minimization to our buffer reference-subtracted data (**Fig 6F**). We derived an experimental standard deviation of the K_d of ± 6 nM, and a standard deviation of the fit (standard error of regression) of the K_d of ± 8 nM.

For our MST measurements (Wienken et al, 2010), we labeled purified Ctf19-Mcm21 with a red fluorescent dye (NT-647-NHS), which covalently modifies lysine sidechains, in company-provided 'labelling buffer' at 22–25 °C, according to the manufacturer's instructions (Nanotemper Technologies), with a 1:4 molar ratio of Ctf19-Mcm21:dye. Labeling efficiency for Ctf19-Mcm21 was ~ 33 %. The low labeling efficiency did not impact our K_d calculations, because the total concentration of labeled and unlabeled Ctf19-Mcm21 that we used in our measurements is lower than the K_d (see equation below). We transferred fluorescently labeled Ctf19-Mcm21 and Okp1-derived peptide with sequence 'SNEIYQQDKIDFNLKLKTDF', or variants of Okp1-derived peptide (**Fig 6G**) to ITC/MST buffer. We prepared dilution series of peptides, and combined with a uniform volume of Ctf19-Mcm21 in PCR tubes, centrifuged them for 3600 sec, before transferring to NT.115 MST premium coated glass capillaries (Nanotemper Technologies). For all our measurements, the total Ctf19-Mcm21 concentration in the capillaries was ~ 27 nM. We measured MST data on an NT.115 Monolith BLUE/RED instrument (Nanotemper Technologies) at 22–25 °C. Samples were heated with an infrared laser (λ: 1474 nm ± 15 nm; max power: 120 mW) set to 20 % MST power (laser on for 30 sec; laser off for 5 sec), and fluorophors excited with a laser (excitation λ: 625 nm; emission λ: 680 nm) set to 60 % LED power. For all our MST measurements, fluorescence-intensity counts were 350–500. We derived our binding data from the 'thermophoresis + T jump' fluorescence signal. For each unique peptide sample, we measured data from three separately prepared dilution series. For our MST data-analyses, we used NTAffinityAnalysis software (version 2.0.2; Nanotemper Technologies). We derived K_d values (**Fig 6G**) by least squares fitting the following law of mass action function, as previously described (Seidel et al, 2013):

$$y = U + (B - U) * \frac{(x + c_{labeled} + K_d - \sqrt{(x + c_{labeled} + K_d)^2 - 4 * x * c_{labeled}})}{2 * c_{labeled}}$$

U: fluorescence of unbound Ctf19-Mcm21 (base level); *B*: fluorescence of bound Ctf19-Mcm21 (saturation level); $c_{labeled}$: concentration of labeled protein; K_d: dissociation constant; *x*: concentration of peptide; *y*: fluorescence.

Culturing, genetic modification, and growth assays of *Saccharomyces cerevisiae*

All our *S. cerevisiae* clones are derivatives of S288C type, unless specified otherwise in our enclosed clone list (**Table EV6**). General *S. cerevisiae* genetic manipulation methods, and media recipes for culturing in liquid medium and growth assays on solid medium were similar as described (Amberg et al, 2005). Most media reagents (bacto yeast extract, bacto peptone, Difco yeast nitrogen base) for *S. cerevisiae* culturing were from Becton Dickinson Biosciences; glucose from Amresco.

For our *Okp1_fl* native locus integration-construct, we amplified from *S. cerevisiae* genomic DNA, in one PCR a fragment of 250 bp of 5' untranslated region (UTR) immediately upstream of *Okp1* and the coding region for Okp1; in a separate PCR, 250 bp 3' UTR immediately downstream of *Okp1*; and from a pU6 plasmid a coding region for a 6×flag epitope. We assembled by PCR, from these fragments, a single DNA fragment, which has an NheI restriction-enzyme site between the 3'UTR and 5'UTR sequences, that we ligated into plasmid pRS305 (Sikorski & Hieter, 1989), with isothermal assembly (Gibson et al, 2009), to generate plasmid *Okp1_fl-6×flag*-pRS305. We generated our *Okp1* integration constructs that lack coding regions for

specific Okp1 segments by Quikchange-based site-directed mutagenesis of *Okp1-6×flag*-pRS305 plasmid with *PfuTurbo* DNA Polymerase AD or *PfuUltra* II Fusion Hot-Start DNA polymerase (Agilent Technologies). For genomic integration in *S. cerevisiae*, we digested our plasmids with NheI (New England BioLabs), transformed into diploid *S. cerevisiae*, and selected single colonies that grew on solid agar with minimal synthetic complete medium without leucine. We sporulated clones in liquid sporulation medium and tetrad dissected on solid agar with yeast-extract pepton dextrose (YPD) medium, with a dissection microscope (MSM, Singer Instruments). Where applicable, we isolated haploid spores and genotyped them on solid agar with minimal synthetic complete medium that lacked one of the following amino acids—adenine, histidine, leucine, lysine, tryptophane, or uracil; and on solid agar with minimal synthetic complete medium that we had plated one of our two mating-type *S. cerevisiae* tester clones (identifiers DDY55 or DDY56; see **Table EV6**) on.

We constructed most of our *S. cerevisiae* clones with specific genes removed or with genes (at native gene loci) encoding C-terminal fluorophor-fusion proteins by PCR-based methods and homologous recombination, as described (Longtine et al, 1998). For protein fusions with C-terminal GFP or myc epitopes, we used plasmids pFA6a–GFP(S65T) kanMX6 or pFA6a–13×myc kanMX6 (Longtine et al, 1998), respectively as templates for PCR products. We transformed PCR products into our haploid cells with *Okp1_fl, Okp1_cmΔ,* or *Okp1_nnΔ*, and selected clones on solid YPD agar with ~ 20 µg/ml or 50 µg/ml geneticin (G-418, Sigma-Aldrich). For C-terminal tagging of Nuf2 with mCherry fluorophor, we used as template a modified pFA6a His3MX6 plasmid that contained mCherry (Stefan Westermann laboratory). We transformed PCR products for tagging Nuf2 with mCherry into our haploid cells with *Okp1_fl* or *Okp1_cmΔ* that encoded Ctf19-GFP or Mcm21-GFP, and selected clones on minimal synthetic complete medium without histidine. For a complete *Ctf19* gene removal or removal of the 5' 954 basepairs of *Ctf19*, we used plasmid pRS303 (Sikorski & Hieter, 1989) as PCR template. We transformed PCR products into diploid *S. cerevisiae*, selected transformants that grew on minimal synthetic medium without histidine, and isolated haploid spores by tetrad dissection, analogously as for *Okp1* clones. For our PCRs, we used Phusion High fidelity DNA polymerase or Phusion Hot Start II DNA polymerase (Thermo Fisher Scientific or New England Biolabs), or Ranger DNA polymerase (Bioline). Except for the PCR product for transformation for our *Ctf19* removal that we transformed directly into *S. cerevisiae*, we gel-purified all PCR products before transformation into *S. cerevisiae*. We verified genomic modifications for gene removals or for genes encoding GFP-fusion kinetochore proteins, with PCRs from isolated genomic DNA and DNA sequencing (except for the modifications of Nuf2-mCherry, Bub1-3×GFP, or GFP-labeled chromosome V (see section 'Live cell fluorescence-image recording and image analyses' below), which we verified with fluorescence microscopy only). The 13×myc epitope-encoding DNA sequence 3' of *Ctf19* frequently recombined, resulting in a number of encoded myc epitopes that was lower than 13. We chose *Ctf19-myc* clones that encoded four myc epitopes.

We generated *Okp1_fl* or *Okp1_cmΔ* with GFP-labeled chromosome V by mating (with clone with identifier SWY285; see **Table EV6**), zygote selection, sporulation in liquid medium, and tetrad dissection. We generated *Okp1_fl* with GFP-labeled Bub1 (Bub1-3×GFP) or *Okp1_cmΔ* with GFP-labeled Bub1 (Bub1-3×GFP), by mating our clones with *Okp1_fl* or *Okp1_cmΔ* with a clone with Bub1-3×GFP (identifier SWY622; *S. cerevisiae* W303 type; see **Table EV6**), selection of clones on minimal synthetic complete medium without leucine and with geneticin, sporulation of

multiple single colonies in liquid medium, and tetrad dissection. We generated our clones *Okp1_fl cnn1Δ or Okp1_cmΔ cnn1Δ,* or our clones *Okp1_fl ctf3Δ or Okp1_cmΔ ctf3Δ* in a similar manner, by mating with a *cnn1Δ* clone (identifier SWY914; see **Table EV6**) or a *ctf3Δ* mutant clone (identifier GPY75; see **Table EV6**), respectively. We generated clones with *Okp1_fl* with *Mcm16-13×myc* or *Okp1_cmΔ* with *Mcm16-13×myc* by mating (identifier GPY97; see **Table EV6**) (Pekgoz Altunkaya et al, 2016), sporulation, and tetrad dissection.

We generated *S. cerevisiae* clones (native, *Okp1_fl*, or *Okp1_cmΔ*) that are compatible for auxin-inducible degradation of a target protein, by first integrating in their *Ura3* genomic locus the *Oryza sativa TIR1* coding region (encoding the F-box transporter inhibitor response 1 protein)—*OsTIR1* under a galactose-inducible promoter, as described (Nishimura et al, 2009). To do so, we transformed clones with plasmid BYP7434 (Kubota et al, 2013) (see **Table EV5**) that we had digested with restriction enzyme Stu**I.** After verifying *OsTIR1* genomic integration, we genetically modified *mad1* so that it encodes Mad1 that is fused at its C terminus to an auxin-inducible degron and nine myc epitopes.

For a list of *S. cerevisiae* clones that we used in our study, see **Table EV6**. Clones (from storage at −80 °C) and PCR primer-sequences for our *S. cerevisiae* genetic modifications are available upon request.

Our growth experiments with *S. cerevisiae* clones that encode Mad1 with an auxin-inducible degron (**Appendix Fig S9E**) were done on solid agar with 2 % (w/v) raffinose and 2 % (w/v) galactose, and 1 mM of the synthetic auxin analogue 1-napthylic acetic acid (NAA; from Carl Roth; dissolved in 5 M NaOH); similarly as described (Nishimura et al, 2009).

Co-immunoprecipitation assays, growth assays in liquid medium of clones with Mad1 degron, and Western blotting

For Western blots that we show images of in **Fig 7B** and **Appendix Fig S8B**,**C**, we grew 50 ml cultures of *S. cerevisiae* clones at 30 °C, shaking at 190 rpm in an orbital shaker, to an A_{600} of 0.7–2.3. After centrifugation at 2500 rpm, we resuspended the cell pellet in a buffer of 25 mM Hepes pH 7.5, 150 mM NaCl, 5 % (v/v) glycerol, 2 mM EDTA pH 8.0, (lysis buffer #2) with 0.5 mM TCEP. We transferred these cell resuspensions to 2 ml microtubes (Sarstedt), centrifuged for 60 sec, discarded the supernatant, froze the pellet in liquid N_2, and transferred the pellets to −80 °C. After thawing, we resuspended the pellet in 700 µl ice-cold lysis buffer #2 with protease inhibitors (Protease inhibitor cocktail set IV (Calbiochem, Merck Millipore), dilution: 10 µl for 1 ml lysis buffer; (final concentration of inhibitors: 1 mM AEBSF, 15 µM E-64, 20 µM Pepstatin A, and 5 mM 1,10-Phenanthroline)) and phosphatase inhibitors (4.9 mM tetra-sodiumdiphosphat decahydrat, 5 mM NaN_3, 10 mM NaF, 0.4 mM Na-orthovanadate). After addition of BioSpec glass beads (0.5 mm diameter, BioSpec Products, VWR), we lysed cells by shaking extracts in microtubes for 120–180 sec in a Minibeadbeater (BioSpec Products) at 4 °C. We added nonyl phenoxypolyethoxylethanol (NP-40 Alternative; Calbiochem) to ~ 0.1 % (v/v), and prepared cleared lysates by centrifugation in a TLA55 rotor at 45000 rpm for 1200 sec in an Ultracentrifuge (Optima Max-XP, Beckmann Coulter), at 4 °C. We transferred the lysate supernatant to protein Lobind microcentrifuge tubes (Eppendorf), and determined total protein concentrations by Bradford protein-assay (with Protein assay dye reagent concentrate; Bio-Rad Laboratories) and bovine serum albumin as standard protein. We coated protein G coupled magnetic beads (Dynabeads, Thermo Fisher Scientific), by adding 50 µl of 1 mg ml^{-1} monoclonal M2

anti-flag antibodies solution (F1804, Sigma-Aldrich) to 100 µl of bead slurry, in phosphate-buffered saline (10.5 mM Na-phosphate, 2.7 mM potassium chloride, 137 mM sodium chloride) pH 7.3 with 0.5 % (v/v) Tween 20. We cross-linked antibodies to Protein G with ~ 20 mM dimethyl pimelimidate (Thermo Fisher Scientific) in 0.2 M Na-borate pH 9.0, for 1800 sec at 22–25 °C. For negative controls, we used protein G-coupled magnetic beads that were not coated with anti-flag antibodies. We blocked beads in lysis buffer #2 with 1 mg ml^{-1} bovine serum albumin for 3600 sec. For co-immunoprecipitation experiments, we used a uniform total protein amount for different samples. We incubated 400 µl of *S. cerevisiae* extracts (total protein amount ~ 3.2 mg) with 30 µl of bovine serum albumin-blocked Dynabeads—either coated with antibodies or uncoated—in microcentrifuge tubes, for ~ 16–18 hrs at 4 °C. We washed Dynabeads in tubes on a magnet, five times with 200 µl lysis buffer #2. After removal of the supernatant, we denatured samples by incubating in 40 µl of SDS sample buffer at 95 °C. We analysed 3 µl and 15 µl of immunoprecipitated samples for Western blots with anti-flag and anti-myc antibodies, respectively (**Fig 7B**; **Appendix Fig S8B**,**C**) on SDS-PAGE gels (10 % Bis-tris gel NuPAGE, with MOPS buffer pH 7.7; Thermo Fisher Scientific). For the input fractions, we loaded a total protein amount of 30 µg or 48 µg on SDS-PAGE for anti-flag and anti-myc blots, respectively. We transferred proteins from SDS-PAGE gel onto a nitrocellulose membrane (0.45 µm, Amersham Protran BA, GE Healthcare Life Sciences) in a buffer of 25 mM Tris, 190 mM glycine, 20 % (v/v) methanol, 0.02 % (w/v) SDS. We verified protein transfer by Ponceau S staining. We probed one membrane with monoclonal anti-flag M2 horse-radish peroxidase (HRP) coupled antibodies (from mouse; A8592, Sigma-Aldrich). We probed a separate membrane with monoclonal 9e10 anti-myc antibodies (from mouse; Covance), and subsequently with an HRP coupled polyclonal anti-mouse antibodies (from goat; Jackson ImmunoResearch laboratories). We added ECL Western blotting detection reagent (GE Healthcare Life Sciences) as HRP substrate, and recorded chemiluminescence on high performance chemiluminescence film (Amersham Hyperfilm ECL; GE Healthcare Life Sciences).

For our co-immunoprecipitation experiment that we show Western-blots images of in **Appendix Fig S8E**,**F**, our protocol was similar to the one that we describe above, with the following deviations. After growing our *S. cerevisiae* clone cultures to an A_{600} of 0.4, we added nocodazole (Sigma-Aldrich) to a concentration of 15 µg ml^{-1}, continued to grow cultures for 135 min, and collected cells by centrifugation. After preparing cell extracts, we froze extracts at −80 °C, before usage. We incubated 400 µl of *S. cerevisiae* extracts (total protein amount ~ 2 mg) with 50 µl of antibodies-coated, crosslinked, and bovine serum albumin-blocked Dynabead slurry, for ~ 16–18 hrs at 4 °C. After incubation, we washed Dynabeads in tubes on a magnet four times with 400 µl of phosphate-buffered saline pH 7.3 with 0.1 % (v/v) Tween 20. We analysed 6 µl and 20 µl of immunoprecipitated samples for Western blots with anti-flag antibodies and anti-myc antibodies, respectively on SDS-PAGE gels that we show Western-blot images of in **Appendix Fig S8E**,**F**. We recorded chemiluminescence with a charge-coupled device of an Amersham Imager 600 (GE Healthcare Life Sciences).

To prepare *S. cerevisiae* extracts for our Western blots that we show an image of in **Appendix Fig S9F**, we grew an initial culture of our clones in liquid YPD medium for ~ 15–18 hrs at 30 °C shaking at 180 rpm in an orbital shaker. We collected cells by centrifugation, washed them twice with yeast-extract pepton (YEP) medium, and resuspended the pellet in 6 ml YEP medium with 2 % (w/v) raffinose and 2 % (w/v) galactose. We used this resuspension to inoculate six 50 ml liquid

cultures of YEP with 2 % (w/v) raffinose and 2 % (w/v) galactose to an A_{600} of 0.3, and grew them at 30 °C shaking at 180 rpm. After two hrs, we collected cells from one of these cultures by centrifugation (time point 0; **Appendix Fig S9F**); and we added NAA to the other five cultures to a concentration of 1 mM. We collected cells from one culture each after specific time points (**Appendix Fig S9F**) by centrifugation. We resuspended cell pellets in 1 ml lysis buffer #2 with protease inhibitors (Protease inhibitor cocktail set IV (Protease inhibitor cocktail set IV (Calbiochem, Merck Millipore); see above) and with 0.5 mM TCEP, centrifuged samples for 60 sec, decanted supernatants, froze cell pellets in liquid nitrogen, and stored them at −80 °C. After thawing, we resuspended these pellets in 350 µl ice-cold lysis buffer #2 with protease inhibitors (Protease inhibitor cocktail set IV (Calbiochem, Merck Millipore); see above) and with 0.5 mM TCEP. We added nonyl phenoxypolyethoxylethanol (NP-40 Alternative; Calbiochem) to ~ 0.1 % (v/v), and prepared cleared lysates by centrifugation in a TLA55 rotor at 45000 rpm in an Ultracentrifuge (Optima Max-XP, Beckmann Coulter), at 4 °C. We transferred lysate supernatants to protein Lobind microcentrifuge tubes (Eppendorf), and determined total protein concentrations by Bradford protein-assay (with Protein assay dye reagent concentrate; Bio-Rad Laboratories) and bovine serum albumin as standard protein. For SDS-PAGE, we loaded a total protein amount of 30 µg. We transferred proteins to a nitrocellulose membrane and incubated with 9e10 anti-myc antibodies and a secondary HRP coupled polyclonal anti-mouse antibodies, as we describe above. We recorded chemiluminescence (**Appendix Fig S9F**) with a charge-coupled device of an Amersham Imager 600 (GE Healthcare Life Sciences).

Live cell fluorescence-image recording and image analyses

For our imaging, we grew *S. cerevisiae* clone cultures at 25 °C shaking at 180 rpm for ~ 16–18 hrs. With this type of culture, we inoculated liquid minimal synthetic complete medium without tryptophane to an A_{600} ~ 0.4. We omitted tryptophane to reduce the background fluorescence-signal in our imaging. We grew cells asynchronously in this medium for ~ 4–5 hrs at 30 °C, shaking at 180 rpm. We prepared cultures of our cells with Nuf2-mCherry analogously, in a similar medium with extra adenine (final concentration: 0.21 % (w/v)). We added adenine, to reduce background red fluorescence-signal originating from adenine biosynthesis. We immobilized our cultured cells (if cell density was too high, we diluted cultures with minimal synthetic complete medium without tryptophane, or without tryptophane and added adenine) on coverslips in glass bottom culture dishes (No. 0 coverglass, 0.085–0.13 mm; MatTek corporation) with concanavalin A (Sigma-Aldrich), and covered immobilized cells in minimal synthetic complete medium without tryptophane or a similar medium with added adenine. For each type of cells with GFP-tagged kinetochore subunits, we imaged at least two isolated clones.

For live cell imaging of *Okp1_fl* with Nkp1-GFP or Nkp2-GFP, or *Okp1_nn*Δ with Nkp1-GFP or Nkp2-GFP (**Fig 5C**), we grew cultures as we describe above. We imaged cells, within ~ 4 hrs after immobilization, at 22–25 °C, by confocal microscopy on an inverted Nikon Ti-E microscope equipped with an Andor AOTF laser combiner and a Yokogawa CSU-X1 spinning disc unit (max. 5000 rpm). We recorded data with an iXON DU-897 monochrome back-illuminated electron multiplying charge-coupled device (EMCCD, Andor Technology) with a 16 µm × 16 µm pixel size, and an image array of 512 pixels × 512 pixels. We used an oil-immersion CFI Apo TIRF objective lens with numerical aperture of 1.49, with 100× magnification; and type F immersion liquid (Leica Camera AG) with refractive index

of 1.518. We excited GFP with a diode laser (50 mW) with 15 % laser power and at 488 nm, and recorded fluorescence at 538 nm (spectral bandwidth: 50 nm). We used an integration time of 0.5 sec for recording fluorescence signal. We controlled acquisition with the Andor IQ3 software (Andor Technology). We recorded 24 stacks, with planes separated by 0.4 µm in the stage position (z) with respect to the objective, on a Prior Scientific NanoScanZ Piezo stage. We analysed and processed our images with Fiji (Schindelin et al, 2012). For image preparation of images that we show in **Fig 5C**, we converted 14-bit depth images to 8-bit depth images.

All our other living *S. cerevisiae* cells (see **Fig 7C**,**D**; **Appendix Fig S8D**), we imaged within ~ 5 hrs after immobilization, at 22–25 °C, with a DeltaVision (Applied Precision, GE Healthcare Life Sciences) wide-field, inverted microscope with a Xenon Lamp (250 Watts) and a Coolsnap HQ charge-coupled device (Photometrics) with a pixel size of 6.45 µm × 6.45 µm. For most of our imaging, we used a 100× magnification, oil-immersion plan-apochromat objective lens (Olympus) with numerical aperture of 1.4, and immersion oil with refractive index of 1.516 (GE healthcare Life Sciences). For each image (image array: 512 pixels × 512 pixels) (see **Fig 7C**,**D**; **Appendix Fig S8D**), we recorded 22 stacks, with planes separated by 0.4 µm in the stage position (z) with respect to the objective. For recording green fluorescence, the microscope-filter settings were: excitation λ: 490 nm (spectral bandwidth: 20 nm), emission λ: 528 nm (spectral bandwidth: 38 nm). For recording of red fluorescence the settings were; excitation λ: 555 nm (spectral bandwidth: 28 nm), emission λ: 617 nm (spectral bandwidth: 73 nm). We used an integration time of 0.6 sec and 0.8 sec for recording green fluorescence signal and red fluorescence signal, respectively for our images that we did not bin. Green fluorescence and red fluorescence signals for each z position were recorded immediately in sequence. For images of cells with Chl4-GFP, Ctf3-GFP, Iml3-GFP, Mcm16-GFP, Mcm22-GFP, Mif2-GFP, Nkp1-GFP, or Nkp2-GFP that we binned 2×2 (see below), we used an integration time of 1 sec for recording green fluorescence signal. We recorded images that we show in **Fig 7C**,**D** and **Appendix Fig S8D** under identical microscopy settings and comparable ambient image-acquisition conditions. We maximum intensity projected fluorescence signals from our stacks to two-dimensional images using softWoRx software (version 6.0.0; DeltaVision, Applied Precision, GE Healthcare Life Sciences). We deconvoluted images (**Fig 7C**,**D**; **Appendix Fig S8D**) with softWoRx. For images of cells with Chl4-GFP, Ctf3-GFP, Iml3-GFP, Mcm16-GFP, Mcm22-GFP, Mif2-GFP, Nkp1-GFP, or Nkp2-GFP (see **Fig 7C**,**D**; **Appendix Fig S8D**), we binned image pixels 2×2 with softWoRx. We analysed and processed our images with Fiji. For figure preparations, we converted deconvoluted and maximum intensity projected images with 16-bit depth to 8-bit depth. We show images in **Figs 5C**,**D**;**7C**,**D**; **Appendix Fig S8D**, for the same GFP-tagged kinetochore subunit in *Okp1_fl* or *Okp1_cmΔ* on the same pixel-value/intensity scale (same minimum pixel-value and maximum pixel-value), to facilitate direct visual comparison.

For live cell imaging of *Okp1_fl* with GFP-labeled chromosome V or *Okp1_cmΔ* with GFP-labeled chromosome V (**Appendix Fig S9C**), we used a 60× magnification plan-apochromat objective with numerical aperture of 1.4 and oil with refractive index of 1.516. Our imaging conditions were generally similar to those that we describe above for imaging with a Deltavision microscope, with minimal synthetic complete medium without tryptophane, with the following modifications; we recorded images with an image array of 1024 pixels × 1024 pixels, 20 stacks with planes separated by 0.4 µm in the stage position (z) with respect to the objective, with an

integration time of 0.6 sec for recording fluorescence signal. For live cell imaging of *Okp1_fl* with Bub1-3×GFP or *Okp1_cmΔ* with Bub1-3×GFP (**Appendix Fig S9D**), our imaging conditions were similar to those that we describe above for imaging on a Deltavision microscope, with minimal synthetic complete medium without tryptophane with extra adenine, with the following difference. We used a 60× plan-apochromat objective with numerical aperture of 1.4; 22 stacks with planes separated by 0.4 μm in the stage position (z) with respect to the objective and an integration time of 0.6 sec or 0.8 sec for recording fluorescence signal; we recorded images with an image array of 1024 pixels × 1024 pixels.

Data-plot preparations
We used Gnuplot (version 5.0.3) for preparation of chromatography plots, light scattering plots, and MST plots for our manuscript figures.

Appendix supplementary references

Adams PD, Afonine PV, Bunkoczi G, Chen VB, Davis IW, Echols N, Headd JJ, Hung LW, Kapral GJ, Grosse-Kunstleve RW, McCoy AJ, Moriarty NW, Oeffner R, Read RJ, Richardson DC, Richardson JS, Terwilliger TC, Zwart PH (2010) PHENIX: a comprehensive Python-based system for macromolecular structure solution. *Acta Crystallogr D* **66:** 213-221

Afonine PV, Grosse-Kunstleve RW, Echols N, Headd JJ, Moriarty NW, Mustyakimov M, Terwilliger TC, Urzhumtsev A, Zwart PH, Adams PD (2012) Towards automated crystallographic structure refinement with phenix.refine. *Acta Crystallogr D* **68:** 352-367

Amberg DC, Burke DJ, Strathern JN (2005) *Methods in Yeast Genetics*: Cold Spring Harbor Laboratory course manual.

Anthis NJ, Clore GM (2013) Sequence-specific determination of protein and peptide concentrations by absorbance at 205 nm. *Protein Science* **22:** 851-858

Benesch JLP, Aquilina JA, Ruotolo BT, Sobott F, Robinson CV (2006) Tandem mass spectrometry reveals the quaternary organization of macromolecular assemblies. *Chemistry & biology* **13:** 597-605

Black BE, Brock MA, Bedard S, Woods VL, Jr., Cleveland DW (2007) An epigenetic mark generated by the incorporation of CENP-A into centromeric nucleosomes. *Proceedings of the National Academy of Sciences of the United States of America* **104:** 5008-5013

Chernushevich IV, Thomson BA (2004) Collisional cooling of large ions in electrospray mass spectrometry. *Anal Chem* **76:** 1754-1760

Dimitrova YN, Jenni S, Valverde R, Khin Y, Harrison SC (2016) Structure of the MIND Complex Defines a Regulatory Focus for Yeast Kinetochore. *Cell* **167:** 1014-1027 e1012

Emsley P, Cowtan K (2004) Coot: model-building tools for molecular graphics. *Acta Crystallogr D Biol Crystallogr* **60:** 2126-2132

Evans PR, Murshudov GN (2013) How good are my data and what is the resolution? *Acta Crystallogr D* **69:** 1204-1214

French S, Wilson K (1978) Treatment of Negative Intensity Observations. *Acta Crystallographica Section A* **34:** 517-525

Gasteiger E, Hoogland C, Gattiker A, Duvaud S, Wilkins MR, Bairoch A (2005) Protein Identification and Analysis Tools of the ExPASy Server. In *The Proteomics Protocols Handbook*, Walker JM (ed), pp 571-607. Humana Press

Gibson DG, Young L, Chuang RY, Venter JC, Hutchison CA, 3rd, Smith HO (2009) Enzymatic assembly of DNA molecules up to several hundred kilobases. *Nat Methods* **6:** 343-345

Gorrec F (2009) The MORPHEUS protein crystallization screen. *Journal of Applied Crystallography* **42:** 1-8

Grimm C, Chari A, Reuter K, Fischer U (2010) A crystallization screen based on alternative polymeric precipitants. *Acta Crystallogr D* **66:** 685-697

Hinshaw SM, Harrison SC (2013) An Iml3-Chl4 heterodimer links the core centromere to factors required for accurate chromosome segregation. *Cell reports* **5:** 29-36

Hornung P, Troc P, Malvezzi F, Maier M, Demianova Z, Zimniak T, Litos G, Lampert F, Schleiffer A, Brunner M, Mechtler K, Herzog F, Marlovits TC, Westermann S (2014) A cooperative mechanism drives budding yeast kinetochore assembly downstream of CENP-A. *The Journal of cell biology* **206:** 509-524

Hu TC, Korczynska J, Smith DK, Brzozowski AM (2008) High-molecular-weight polymers for protein crystallization: poly-gamma-glutamic acid-based precipitants. *Acta Crystallogr D* **64:** 957-963

Kabsch W (1993) Automatic processing of rotation diffraction data from crystals and initially unknown symmetry and cell constants. *J Appl Cryst* **26:** 795-800

Kapust RB, Tozser J, Fox JD, Anderson DE, Cherry S, Copeland TD, Waugh DS (2001) Tobacco etch virus protease: mechanism of autolysis and rational design of stable mutants with wild-type catalytic proficiency. *Protein Eng* **14:** 993-1000

Karplus PA, Diederichs K (2012) Linking crystallographic model and data quality. *Science* **336:** 1030-1033

Kubota T, Nishimura K, Kanemaki MT, Donaldson AD (2013) The Elg1 replication factor C-like complex functions in PCNA unloading during DNA replication. *Molecular cell* **50:** 273-280

Kupniewska-Kozak A, Gospodarska E, Dadlez M (2010) Intertwined Structured and Unstructured Regions of exRAGE Identified by Monitoring Hydrogen-Deuterium Exchange. *Journal of molecular biology* **403:** 52-65

Longtine MS, McKenzie A, Demarini DJ, Shah NG, Wach A, Brachat A, Philippsen P, Pringle JR (1998) Additional modules for versatile and economical PCR-based gene deletion and modification in Saccharomyces cerevisiae. *Yeast* **14:** 953-961

McCoy A, Grosse-Kunstleve R, Adams P, Winn M, Storoni L, Read R (2007) Phaser crystallographic software. *J Appl Cryst* **40:** 658-674

Miller MP, Unal E, Brar GA, Amon A (2012) Meiosis I chromosome segregation is established through regulation of microtubule-kinetochore interactions. *eLife* **1:** e00117

Nettleton EJ, Sunde M, Lai ZH, Kelly JW, Dobson CM, Robinson CV (1998) Protein subunit interactions and structural integrity of amyloidogenic transthyretins: Evidence from electrospray mass spectrometry. *Journal of molecular biology* **281:** 553-564

Newman J, Egan D, Walter TS, Meged R, Berry I, Ben Jelloul M, Sussman JL, Stuart DI, Perrakis A (2005) Towards rationalization of crystallization screening for small- to medium-sized academic laboratories: the PACT/JCSG plus strategy. *Acta Crystallogr D* **61:** 1426-1431

Nishimura K, Fukagawa T, Takisawa H, Kakimoto T, Kanemaki M (2009) An auxin-based degron system for the rapid depletion of proteins in nonplant cells. *Nature Methods* **6:** 917-U978

Pekgoz Altunkaya G, Malvezzi F, Demianova Z, Zimniak T, Litos G, Weissmann F, Mechtler K, Herzog F, Westermann S (2016) CCAN Assembly Configures Composite Binding Interfaces to Promote Cross-Linking of Ndc80 Complexes at the Kinetochore. *Current biology : CB* **26:** 2370-2378

Pringle SD, Giles K, Wildgoose JL, Williams JP, Slade SE, Thalassinos K, Bateman RH, Bowers MT, Scrivens JH (2007) An investigation of the mobility separation of some peptide and protein ions using a new hybrid quadrupole/travelling wave IMS/oa-ToF instrument. *Int J Mass Spectrom* **261:** 1-12

Radaev S, Li S, Sun PD (2006) A survey of protein-protein complex crystallizations. *Acta Crystallogr D* **62:** 605-612

Read RJ (1986) Improved Fourier Coefficients for Maps Using Phases from Partial Structures with Errors. *Acta Crystallographica Section A* **42:** 140-149

Schindelin J, Arganda-Carreras I, Frise E, Kaynig V, Longair M, Pietzsch T, Preibisch S, Rueden C, Saalfeld S, Schmid B, Tinevez JY, White DJ, Hartenstein V, Eliceiri K, Tomancak P, Cardona A (2012) Fiji: an open-source platform for biological-image analysis. *Nat Methods* **9:** 676-682

Schmitzberger F, Harrison SC (2012) RWD domain: a recurring module in kinetochore architecture shown by a Ctf19-Mcm21 complex structure. *EMBO reports* **13:** 216-222

Seidel SAI, Dijkman PM, Lea WA, van den Bogaart G, Jerabek-Willemsen M, Lazic A, Joseph JS, Srinivasan P, Baaske P, Simeonov A, Katritch I, Melo FA, Ladbury JE, Schreiber G, Watts A, Braun D, Duhr S (2013) Microscale thermophoresis quantifies biomolecular interactions under previously challenging conditions. *Methods* **59:** 301-315

Sharon M, Robinson CV (2007) The role of mass spectrometry in structure elucidation of dynamic protein complexes. *Annual review of biochemistry* **76:** 167-193

Sikorski RS, Hieter P (1989) A System of Shuttle Vectors and Yeast Host Strains Designed for Efficient Manipulation of DNA in Saccharomyces-Cerevisiae. *Genetics* **122:** 19-27

Sobott F, Hernandez H, McCammon MG, Tito MA, Robinson CV (2002) A tandem mass spectrometer for improved transmission and analysis of large macromolecular assemblies. *Anal Chem* **74:** 1402-1407

Tan S (2001) A modular polycistronic expression system for overexpressing protein complexes in Escherichia coli. *Protein Expr Purif* **21:** 224-234

Wienken CJ, Baaske P, Rothbauer U, Braun D, Duhr S (2010) Protein-binding assays in biological liquids using microscale thermophoresis. *Nature communications* **1**

Winn MD, Ballard CC, Cowtan KD, Dodson EJ, Emsley P, Evans PR, Keegan RM, Krissinel EB, Leslie AGW, McCoy A, McNicholas SJ, Murshudov GN, Pannu NS, Potterton EA, Powell HR, Read RJ, Vagin A, Wilson KS (2011) Overview of the CCP4 suite and current developments. *Acta Crystallogr D* **67:** 235-242
