# Supplementary material for: Molecular basis for inner kinetochore configuration through RWD domain–peptide interactions
Source: EMBO J. 2017 Oct 18;36(23):3458–82. doi: 10.15252/embj.201796636 (PMC5709738; doi:10.15252/embj.201796636)
Supplement: Supplementary file 2 — Expanded View Figures PDF [file EMBJ-36-3458-s002.pdf]

Expanded View Figures

Figure EV1. Dimeric reconstituted *K. lactis* COMA-MIND and topology of reconstituted *K. lactis* COMA.

A Nanoflow mass spectrum of reconstituted *K. lactis* COMA-MIND sprayed in 200 mM ammonium acetate (pH 6.7–7.3), which shows high-intensity signals for COMA-MIND dimers; core *K. lactis* MIND variant (MIND-C1, see Dimitrova *et al*, 2016) with Dsn1 residues 230–479 (with an N-terminal SNA residual), Mtw1 residues 1–233, full-length Nnf1, full-length Nsl1; expected mass for Mcm21 includes N-terminal SNA residual.

B Representative nanoflow mass spectrum of COMA acquired by spraying in 200 mM ammonium acetate (pH 6.7–7.3), showing COMA dimers. We selected the 41+ charge state of dimeric COMA for tandem mass spectrometry (inset mass spectrum) applying high collision energy in the gas phase. This species gave rise to COMA dimers that were stripped of subunits. Expected mass for Mcm21 includes polyhistidine tag.

© 2017 The Authors

A Nanoflow mass-spectrum of COMA-MIND

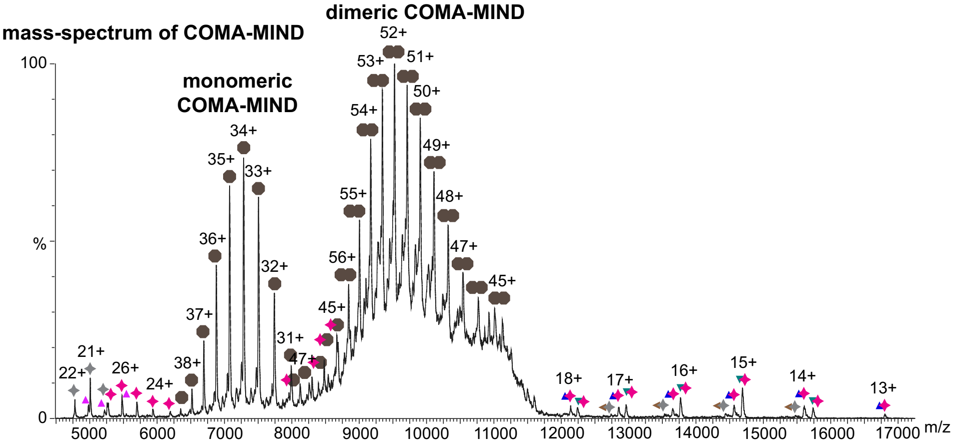

Protein assembly	Expected molecular mass (Da)	Measured molecular mass (Da)
Dsn1-Mtw1-Nsl1	81556	81302 ± 4
Ame1-Ctf19-Mcm21-Okp1 (COMA)	142337	142408 ± 17
Dsn1-Mtw1-Nnf1-Nsl1 (MIND)	105443	105139 ± 4
Ame1-Mcm21-Okp1-MIND	216975	216622 ± 26
Ame1-Ctf19-Mcm21-Okp1-Dsn1-Nnf1-Nsl1 (COMA-Dsn1-Nnf1-Nsl1)	220426	220339 ± 22
Ame1-Ctf19-Mcm21-Okp1-Mtw1-Nnf1-Nsl1 (COMA-Mtw1-Nnf1-Nsl1)	218733	218494 ± 10
Ame1-Ctf19-Mcm21-Okp1-Dsn1-Mtw1-Nnf1-Nsl1 (COMA-MIND)	247780	247681 ± 14
$Ame1_2$-$Ctf19_2$-$Mcm21_2$-$Okp1_2$-Dsn1-Mtw1-Nnf1-Nsl1 ($COMA_2$-MIND)	390118	390031 ± 32
$Ame1_2$-$Ctf19_2$-$Mcm21_2$-$Okp1_2$-$Dsn1_2$-$Mtw1_2$-$Nnf1_2$-$Nsl1_2$ ($(COMA\text{-}MIND)_2$)	495560	495286 ± 32

B Nanoflow mass-spectra of COMA

Protein or protein assembly	Expected molecular mass (Da)	Measured molecular mass (Da)
Ctf19	30805	30808 ± 5
Mcm21	35845	35852 ± 6
Ctf19-Mcm21	66650	66677 ± 17
Ame1-Ctf19-Okp1	108544	108449 ± 15
Ame1-Mcm21-Okp1	113584	113481 ± 32
Ctf19-Mcm21-Okp1	111536	111405 ± 4
Ame1-Ctf19-Mcm21-Okp1 (COMA)	144389	144375 ± 16
$Ame1_2$-$Ctf19_2$-$Mcm21_2$-$Okp1_2$ ($COMA_2$)	288777	288764 ± 22
$Ame1_2$-$Ctf19_2$-Mcm21-$Okp1_2$ (COMA-Ame1-Ctf19-Okp1)	252933	252840 ± 16
$Ame1_2$-Ctf19-$Mcm21_2$-$Okp1_2$ (COMA-Ame1-Mcm21-Okp1)	257972	257908 ± 15
$Ame1_2$-Ctf19-Mcm21-$Okp1_2$ (COMA-Ame1-Okp1)	222128	222025 ± 19
DnaK (from *Escherichia coli*)	69115	69022 ± 9

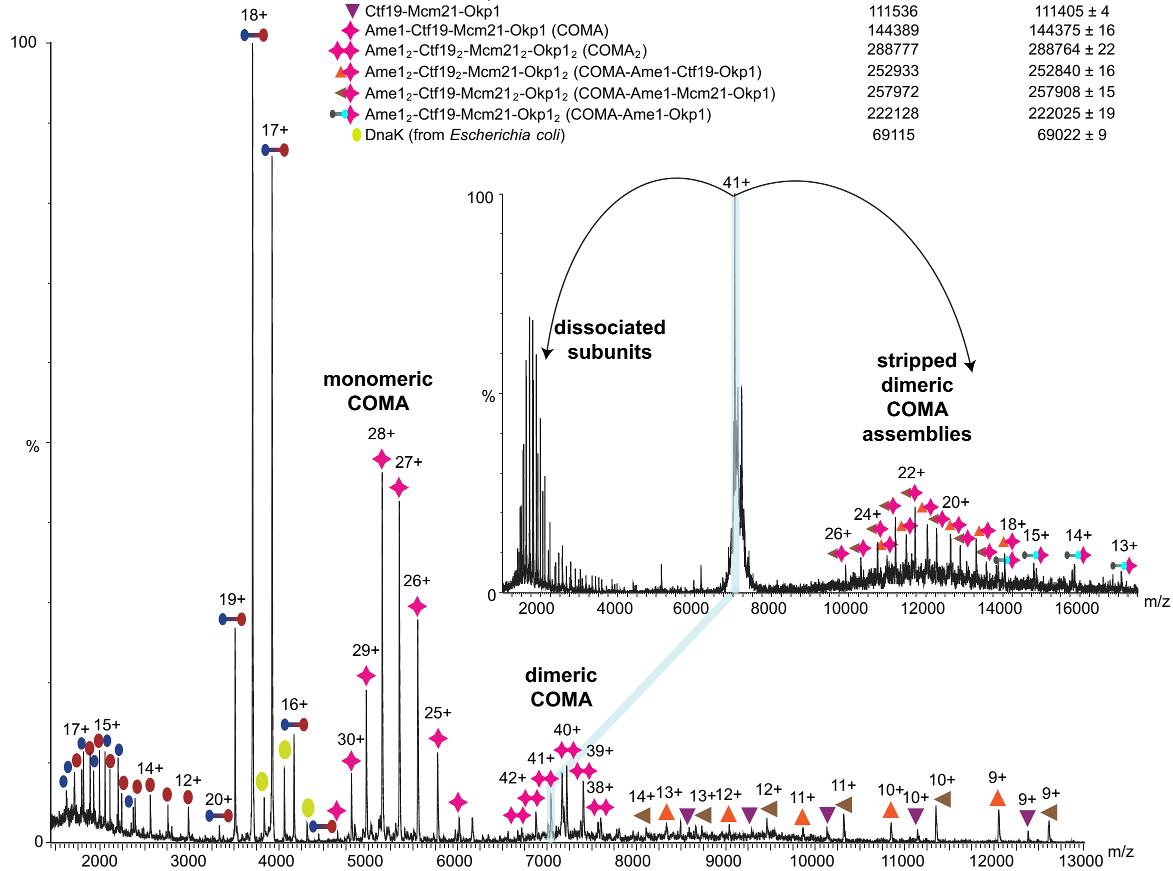

Figure EV1.

Figure EV2. Subunit topology of *K. lactis* COMA-Nkp1-Nkp2.

A Representative nanoflow mass spectrum of COMA-Nkp1-Nkp2 acquired with COMA-Nkp1-Nkp2 at a concentration of ~3 μM, in 200 mM ammonium acetate (pH 7.4), showing charge states for COMA-Nkp1-Nkp2, COMA-Nkp1-Nkp2 dimers, stripped dimeric COMA-Nkp1-Nkp2 subassemblies and single subunits. The COMA-Nkp1-Nkp2 sample that we used for our presented experiment contained a mixture of polyhistidine-tagged Nkp1 (HisNkp1) and Nkp1 without polyhistidine tag (with an N-terminal SNA residual). Expected mass for Mcm21 includes an N-terminal SNA residual.

B Mass spectrum from tandem mass spectrometry of the 33+ charge state of COMA-Nkp1-Nkp2 (shown in A). After applying high collision energy in the gas phase, we observed dissociated subunits or dissociated dimers that were expelled from COMA-Nkp1-Nkp2 in the m/z range of 1,000–4,500, and COMA-Nkp1-Nkp2 subassemblies of which subunits were stripped (stripped pentamers) in the m/z range of 6,600–16,000.

© 2017 The Authors

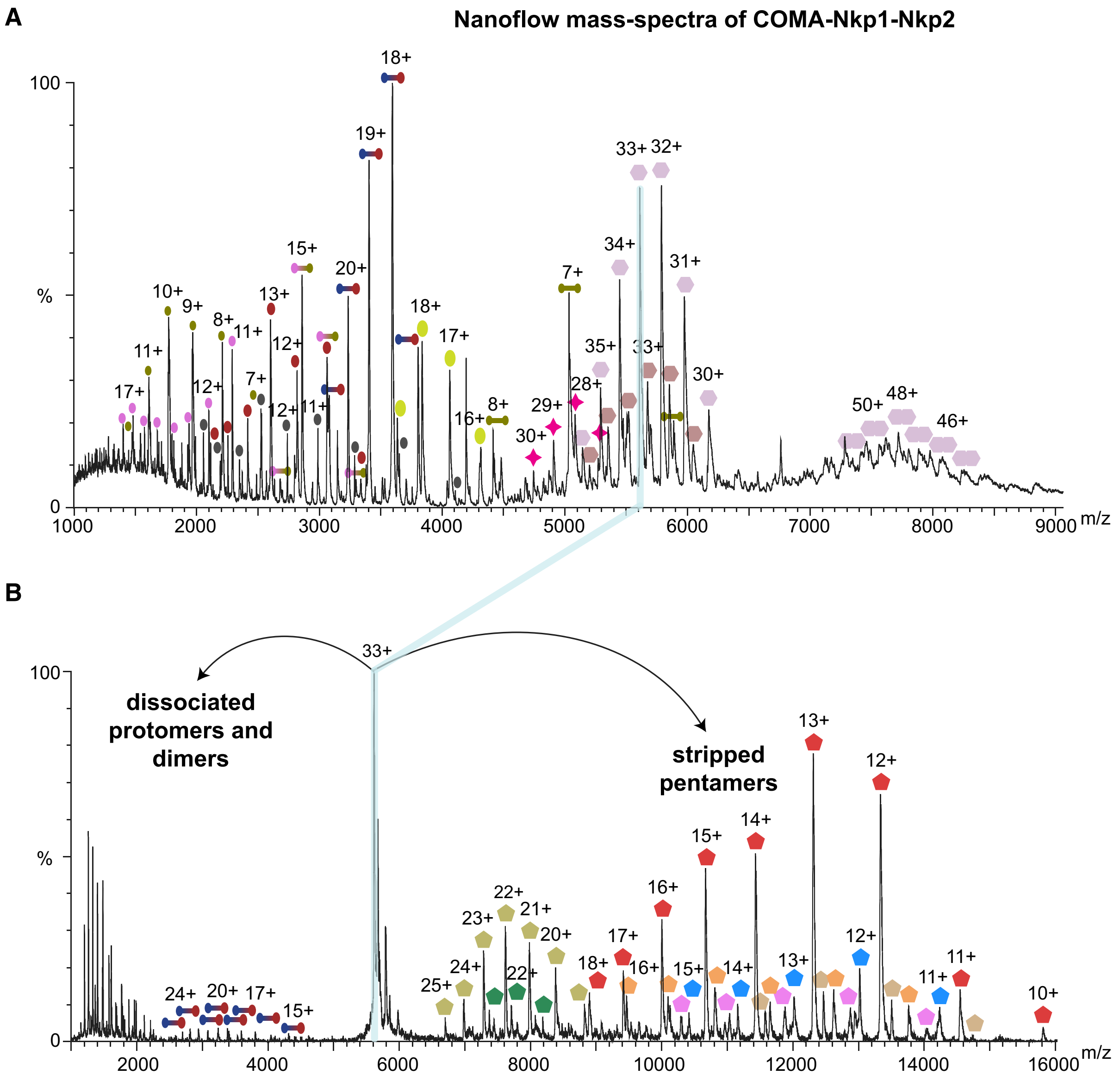

Protein or protein assembly	Expected molecular mass (Da)	Measured molecular mass (Da)
Ame1	32853	32855 ± 12
Mcm21	33793	33800 ± 14
Nkp1	25164	25169 ± 7
Nkp2	17658	17672 ± 18
Ctf19-Mcm21	64599	64729 ± 26
Nkp1-Nkp2	42822	42897 ± 11
Nkp2-Nkp2	35316	35318 ± 21
Ame1-Ctf19-Mcm21-Okp1 (COMA)	142337	142448 ± 12
Ame1-Ctf19-Mcm21-Okp1-HisNkp1 (COMA-HisNkp1)	169552	169524 ± 28
Ame1-Ctf19-Mcm21-Okp1-Nkp1 (COMA-Nkp1)	167501	167569 ± 23
Ame1-Ctf19-Mcm21-Okp1-Nkp2 (COMA-Nkp2)	159996	160021 ± 15
Ame1-Ctf19-Okp1-Nkp1-Nkp2	151366	151445 ± 24
Ame1-Mcm21-Okp1-HisNkp1-Nkp2	156405	156169 ± 28
Ame1-Mcm21-Okp1-Nkp1-Nkp2	154354	154445 ± 24
Ctf19-Mcm21-Okp1-HisNkp1-Nkp1	161863	162019 ± 9
Ame1-Ctf19-Mcm21-Okp1-Nkp1-Nkp2 (COMA-HisNkp1-Nkp2)	187210	187350 ± 22
Ame1-Ctf19-Mcm21-Okp1-Nkp1-Nkp2 (COMA-Nkp1-Nkp2)	185159	185317 ± 20
Ame1$_2$-Ctf19$_2$-Mcm21$_2$-Okp1$_2$-Nkp1$_2$-Nkp2$_2$ ((COMA-Nkp1-Nkp2)$_2$)	370318	370861 ± 65
DnaK (from *Escherichia coli*)	69115	69116 ± 3

Figure EV2.

Figure EV3. Ame1-Okp1 binds Nkp1-Nkp2.

A (Left) Representative overlayed SEC chromatograms showing absorbance at 280 nm, of *K. lactis* Ctf19-Mcm21 (CM), *K. lactis* Nkp1-Nkp2 (NN) and Ctf19-Mcm21 combined with Nkp1-Nkp2 (CM-NN). (Right) Image of SDS–PAGE gel of principal peak fractions from SEC of CM-NN.

B (Top) Representative overlayed SEC chromatograms showing absorbance at 280 nm, of recombinant *S. cerevisiae* Ame1-Okp1 (AO) with polyhistidine-tagged Ame1 (Ame1-His), recombinant *S. cerevisiae* Nkp1-Nkp2 (NN) with polyhistidine-tagged Nkp1 (His-Nkp1) and of a sample of Ame1-His-Okp1 combined with His-Nkp1-Nkp2 (AO-NN). (Bottom) Images of SDS–PAGE gels that we analysed equivalent elution fractions from SEC on. The SEC elution volume range of principal peak fractions that we analysed with SDS–PAGE is indicated below the chromatogram.

© 2017 The Authors

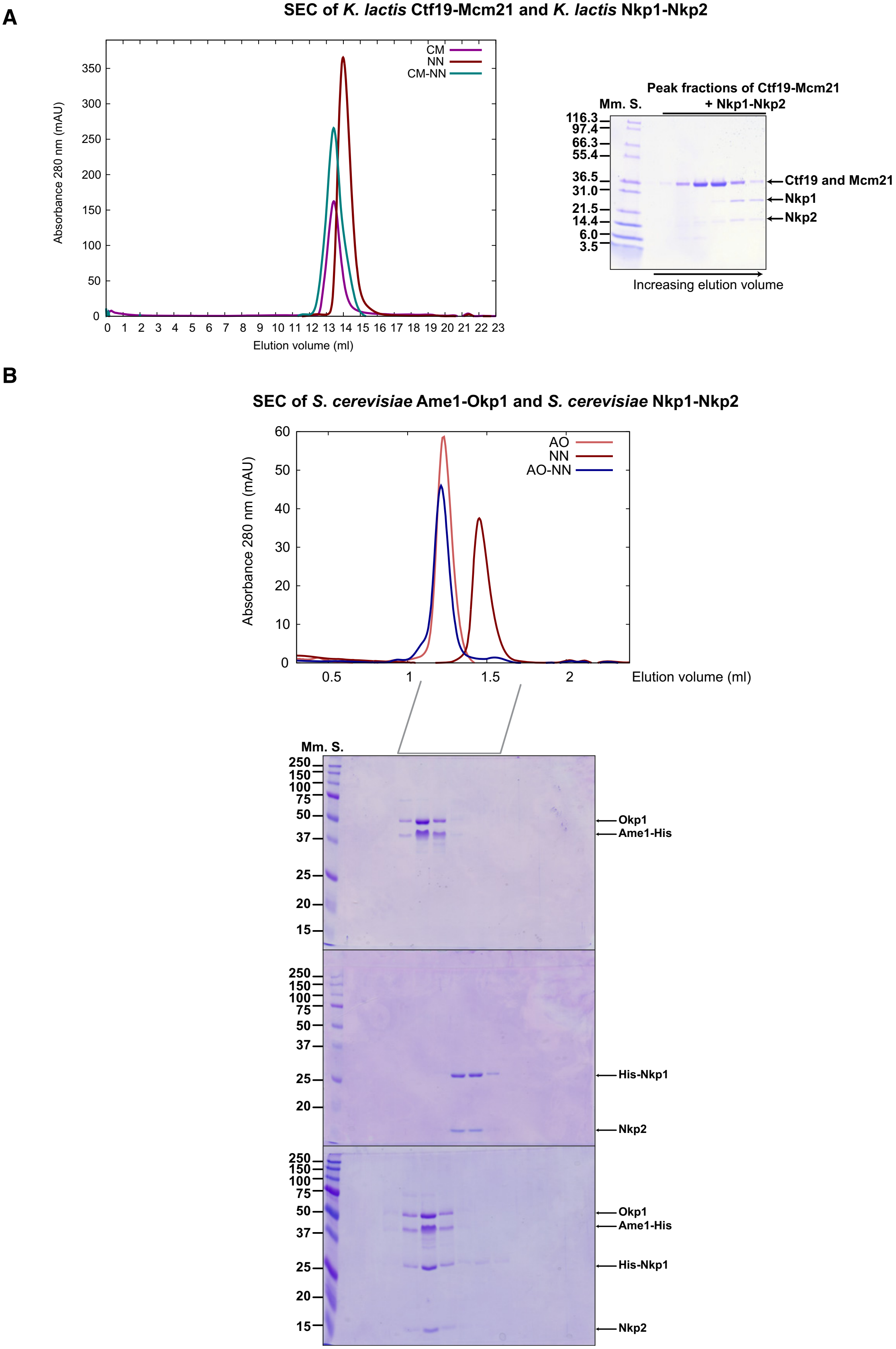
A
SEC of K. lactis Ctf19-Mcm21 and K. lactis Nkp1-Nkp2
CM
NN
CM-NN
Absorbance 280 nm (mAU)
Elution volume (ml)
Peak fractions of Ctf19-Mcm21 + Nkp1-Nkp2
Mm. S.
116.3
97.4
66.3
55.4
36.5
31.0
21.5
14.4
6.0
3.5
Ctf19 and Mcm21
Nkp1
Nkp2
Increasing elution volume
B
SEC of S. cerevisiae Ame1-Okp1 and S. cerevisiae Nkp1-Nkp2
AO
NN
AO-NN
Absorbance 280 nm (mAU)
Elution volume (ml)
Mm. S.
250
150
100
75
50
37
25
20
15
Okp1
Ame1-His
His-Nkp1
Nkp2

Figure EV3.

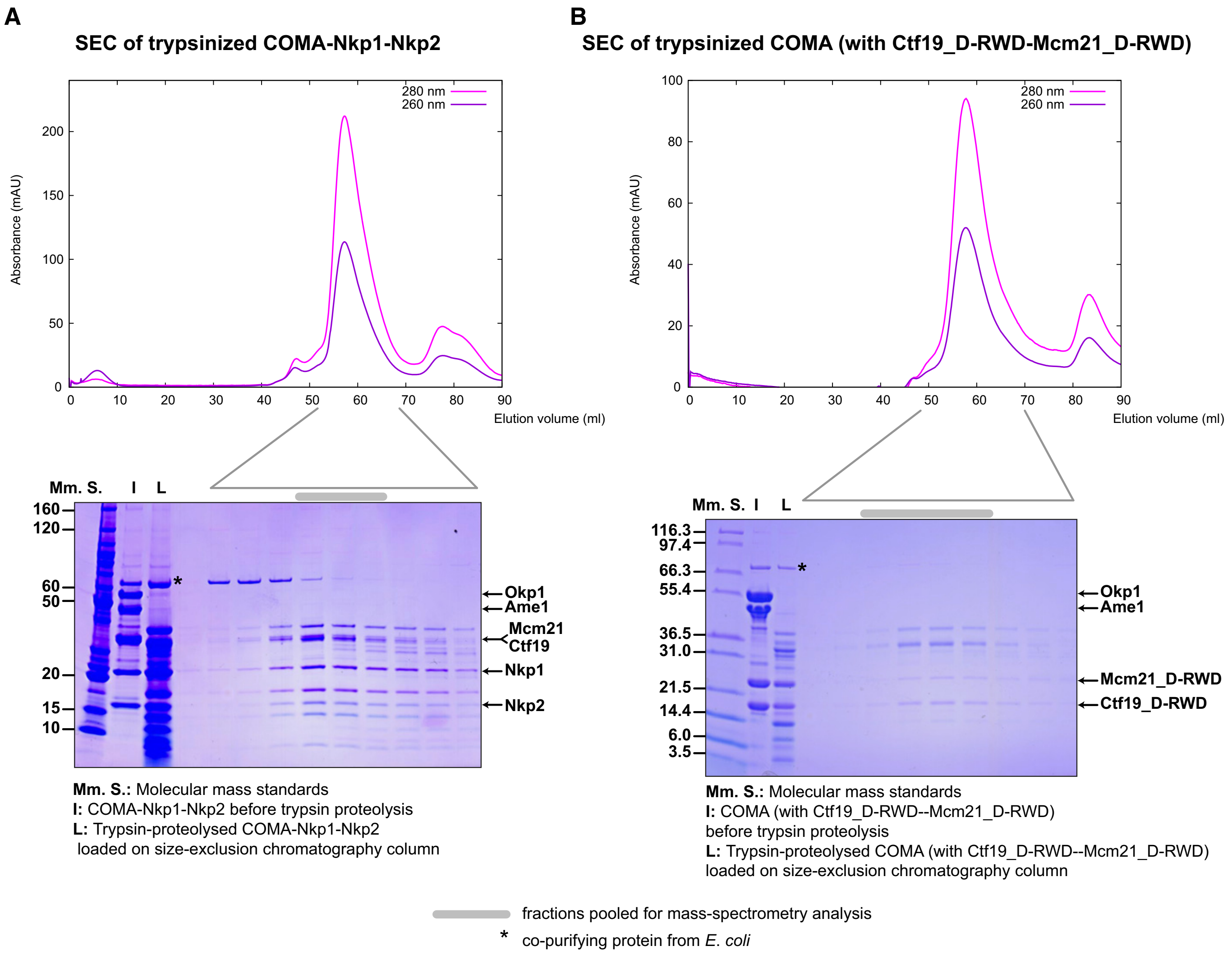

Protein fragment identified by mass spectrometry	Molecular mass (Da)
Ame1 11–281	30460
Ame1 22–292(C terminus)	30343
Ctf19 102–270	19407
Mcm21 90–163	8472
Mcm2 164–293(C terminus)	15138
Okp1 111–361	29725
Okp1 111–383(C terminus)	32178
Okp1 117–361	29036
Okp1 117–383(C terminus)	31490
Nkp1	25163
Nkp2	17658

Protein fragment identified by mass spectrometry	Molecular mass (Da)
Ame1 22–273	28321
Ame1 22–274	28449
Ctf19 107–270	19028
Mcm21 108–293	22045
Okp1 111–361	29725
Okp1 117–361	29036

Figure EV4.

 © 2017 The Authors

Figure EV4. Comparative limited proteolysis of COMA-Nkp1-Nkp2 and COMA.

A, B Representative SEC chromatograms and images of SDS–PAGE gels with fractions from the principal SEC peak of full-length COMA-Nkp1-Nkp2—after 240 s of incubation with trypsin (10:1 molar ratio of COMA-Nkp1-Nkp2 to trypsin) (A), or COMA with full-length Ame1 and Okp1, and $Ctf19_{D\text{-}RWD}$-$Mcm21_{D\text{-}RWD}$—after 255 s of incubation with trypsin (10:1 molar ratio of COMA to trypsin) (B). Migrating positions of full-length COMA-Nkp1-Nkp2 proteins, or the N-terminally truncated Ctf19 or Mcm21 versions—$Ctf19_{D\text{-}RWD}$ (residues 107–270) or $Mcm21_{D\text{-}RWD}$ (residues 108–293), are indicated by arrows. Pooled elution fractions that we used for our mass spectrometry analysis are indicated by a grey bar above SDS–PAGE gel images.

Data information: inset tables at the bottom of each panel list protein fragments (numbers are amino acid residue numbers) identified by mass spectrometry, from the pooled fractions, and their molecular masses (expected masses for Mcm21, $Mcm21_{D\text{-}RWD}$ or Nkp1 include an SNA residual; $Ctf19_{D\text{-}RWD}$ has an M at the N-terminus). Full-length Okp1 in our recombinant *K. lactis* COMA samples was prone to spontaneous proteolysis (see Fig 1A). In mass spectra of purified $Ctf19_{D\text{-}RWD}$-$Mcm21_{D\text{-}RWD}$ with Okp1 fragments that we separated chromatographically from Ame1-$Ctf19_{D\text{-}RWD}$-$Mcm21_{D\text{-}RWD}$-Okp1 by anion-exchange chromatography, we found Okp1 peptides for residues 234–383, the product of spontaneous proteolysis of full-length Okp1. We found a fragment of similarly N-terminally truncated Okp1 (residues 234–336) in samples of our purified $Ctf19_{D\text{-}RWD}$-$Mcm21_{D\text{-}RWD}$-$Okp1_{123\text{–}336}$ or $Ctf19_{D\text{-}RWD}$-$Mcm21_{D\text{-}RWD}$-$Okp1_{106\text{–}336}$ variants. Comparison of the deuterium-exchange patterns of peptides from $Ctf19_{D\text{-}RWD}$-$Mcm21_{D\text{-}RWD}$ with co-purifying Okp1 fragments, which we had purified from the production of COMA (Ame1-$Ctf19_{D\text{-}RWD}$-$Mcm21_{D\text{-}RWD}$-Okp1), with the exchange patterns of those from $Ame1_{1\text{–}260}$-$Okp1_{123\text{–}336}$ showed us that Okp1 segment 2 exchanged rapidly in the absence of Ame1, but was protected with Ame1 bound. We subsequently found an Okp1 fragment that spanned residues 296–361 in samples from our limited proteolysis reactions of COMA-Nkp1-Nkp2 (Table EV2). And we found fragments spanning a similar residue range in mass spectra of purified samples of $Ctf19_{D\text{-}RWD}$-$Mcm21_{D\text{-}RWD}$-$Okp1_{106\text{–}336}$ or $Ctf19_{D\text{-}RWD}$-$Mcm21_{D\text{-}RWD}$-$Okp1_{229\text{–}336}$, from which Okp1 variants had spontaneously proteolysed (see manuscript text).

Figure EV5. Sequence conservation of Okp1 structured segments.

Multiple sequence alignment of Okp1 orthologues from budding yeasts *Kluyveromyces lactis* (Refseq accession code: XP_455759.1), *Saccharomyces cerevisiae* (NP_011695.1), *Vanderwaltozyma polyspora* (XP_001646874.1), *Zygosaccharomyces rouxii* (XP_002495422.1), *Eremothecium gossypii* (NP_982404.1), *Lachancea thermotolerans* (XP_002551957.1), *Candida glabrata* (XP_446545.1). Among the sequences shown, identical amino acid residues are on red background and similar residues are in red letters. Every 10th residue in the *K. lactis* Okp1 sequence is numbered above the alignment. Positions of proteolysis sites from fragments of our limited proteolysis experiments (see Tables EV1 and EV2) are indicated with vertical arrows. Structured segments and predicted coiled-coil regions are as we show in Fig 3A.

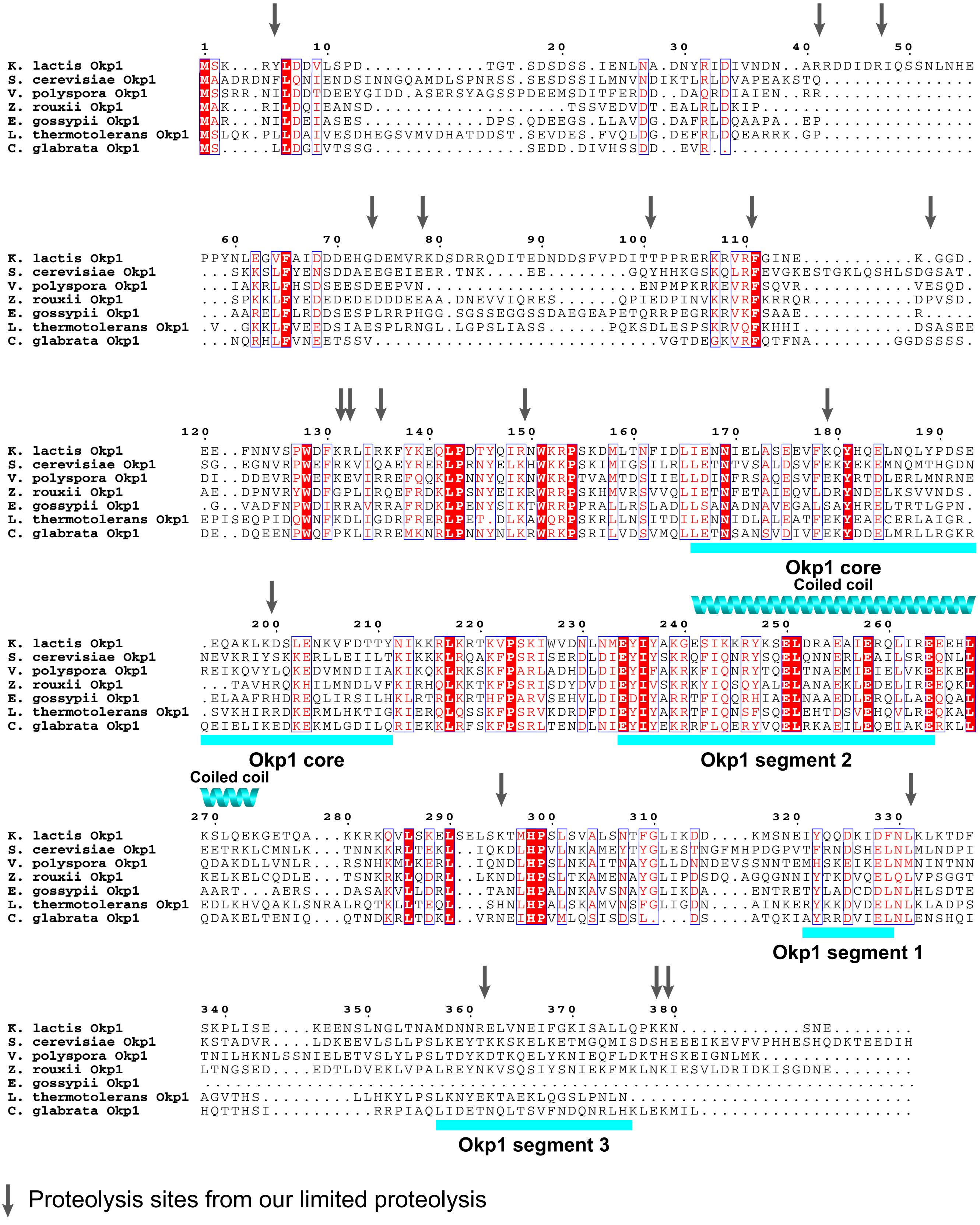

Figure EV5.

 © 2017 The Authors

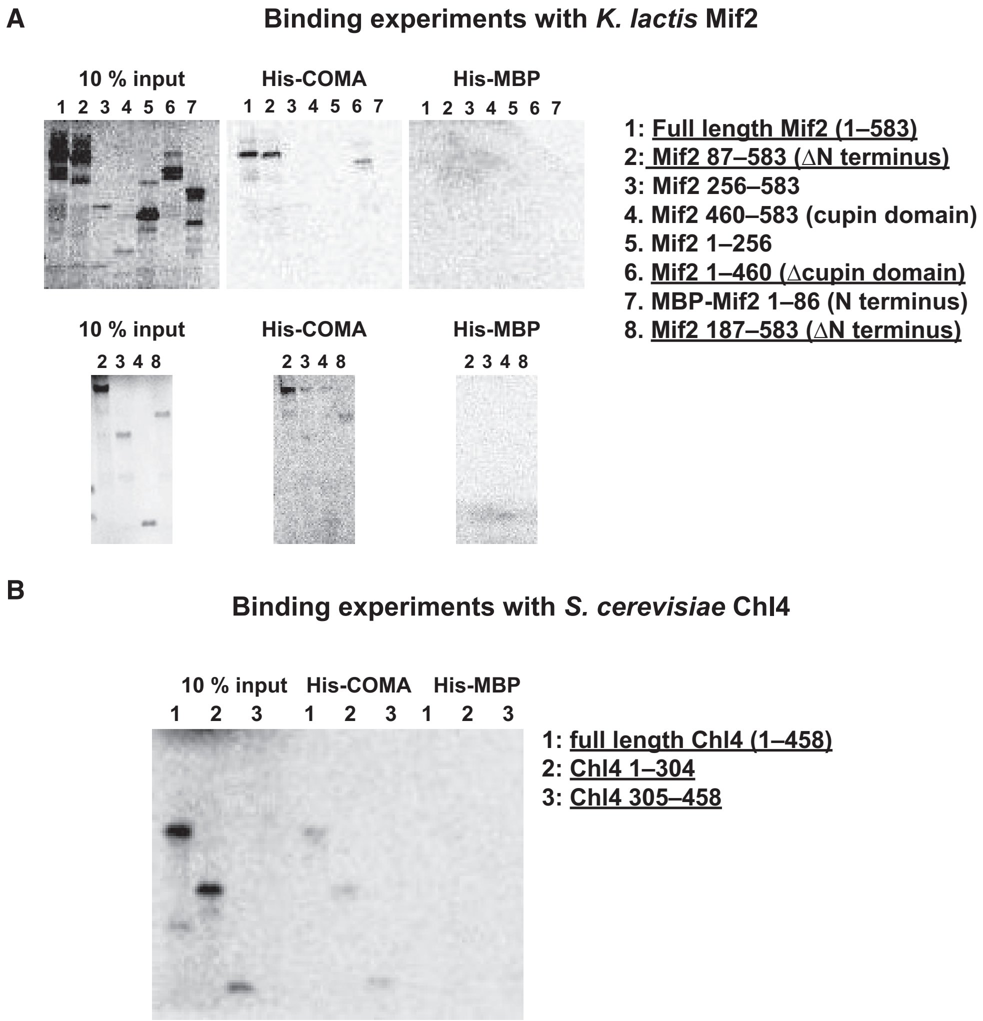

Figure EV6. Interactions of COMA with CCAN proteins.

A Representative images of radiographs with phosphorescence signal from binding experiments of *K. lactis* COMA with polyhistidine-tagged Mcm21 (His-COMA) and *in vitro* translated S^{35}-labelled samples of *K. lactis* Mif2 or truncated *K. lactis* Mif2 variants. 10% (v/v) input fractions are shown. For this experiment and the one that we show in (B), we used polyhistidine-tagged maltose binding protein (His-MBP) as a negative control. The Mif2 N-terminal domain that contains the MIND binding site and the Mif2 C-terminal cupin-fold domain are dispensable for binding COMA.

B Representative image of radiographs with phosphorescence signal from binding experiments of *S. cerevisiae* COMA with polyhistidine-tagged Ame1 (His-COMA), and *in vitro* translated S^{35}-labelled samples of *S. cerevisiae* Chl4 or truncated *S. cerevisiae* Chl4 variants.

Figure EV7. Multiple sequence alignments of Okp1 orthologues from yeasts and CENP-Q orthologues from animals. ▶

Multiple sequence alignment of Okp1 orthologues from budding yeasts (sequences as in Fig EV5), *Schizosaccharomyces pombe* Fta7 (Refseq accession code: NP_587733.1) and CENP-Q orthologues from *Homo sapiens* (Q7L2Z9), *Rattus norvegicus* (NP_001014237.1), *Bos taurus* (NP_001071533.1), *Gallus gallus* (NP_001038114.1). Structured segments that we identified with our analyses (see Fig 3A) are indicated below the alignment. Alignment formatting and annotations are analogous to that for the alignment in Fig EV5.

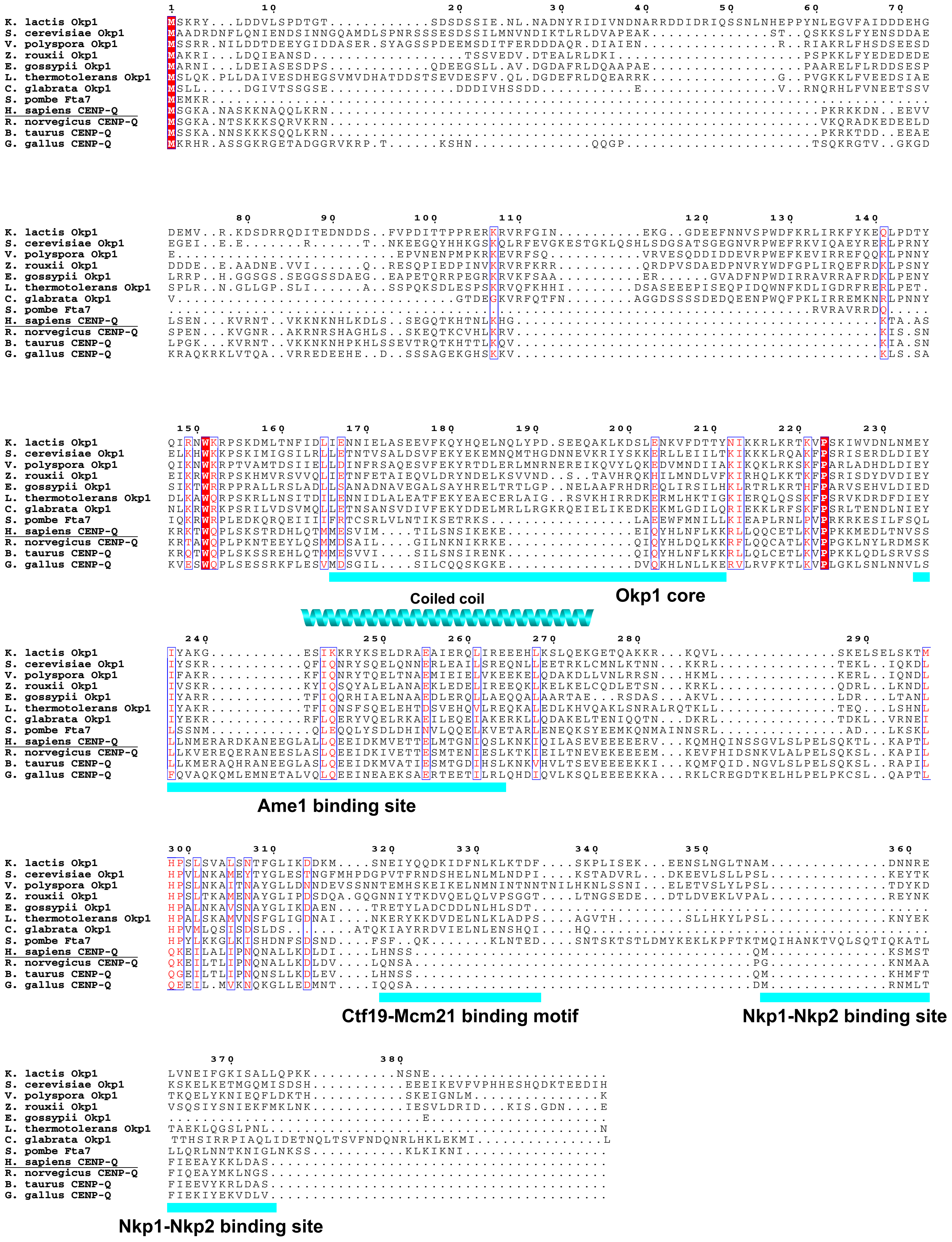

Figure EV7.

 © 2017 The Authors
