# Supplementary material for: Molecular basis for inner kinetochore configuration through RWD domain–peptide interactions
Source: EMBO J. 2017 Oct 18;36(23):3458–82. doi: 10.15252/embj.201796636 (PMC5709738; doi:10.15252/embj.201796636)
Supplement: Supplementary file 3 — Table EV1 [file EMBJ-36-3458-s003.docx]

## Table EV1: Protein fragments identified in mass spectra from our limited proteolysis experiments with trypsin or elastase of *K. lactis* COMA-Nkp1-Nkp2, after size exclusion chromatography

| Protein | Fragment (residue numbers *K. lactis* proteins) |
| --- | --- |
| Ame1 | 11–281, 22–292, 104–292 |
| Ctf19 | 53–207, 102–270 |
| Mcm21 | 35–138, 90–163, 64–293, 164–293 |
| Okp1 | 5–135, 101–383, 111–361, 111–383, 117–361, 117–383, 132–199, 133–378, 133–379 |
| Nkp1 | 1–97 |
| Nkp2 | 1–97, 1–115, 71–139 |

from multiple experiments
