# Supplementary material for: Molecular basis for inner kinetochore configuration through RWD domain–peptide interactions
Source: EMBO J. 2017 Oct 18;36(23):3458–82. doi: 10.15252/embj.201796636 (PMC5709738; doi:10.15252/embj.201796636)
Supplement: Supplementary file 4 — Table EV2 [file EMBJ-36-3458-s004.docx]

## Table EV2: Protein fragments identified in mass spectra from our limited proteolysis experiments with trypsin or elastase of *K. lactis* COMA-Nkp1-Nkp2, without subsequent chromatography

| Protein | Fragment (residue numbers *K. lactis* proteins) |
| --- | --- |
| Ame1 | 98–292, 104–292 |
| Ctf19 | 2–75, 102–270, 105–261 |
| Mcm21 | 1–16, 23–58, 90–163, 91–293, 106–293 |
| Okp1 | 1–131, 42–78, 48–78, 74–331, 150–179, 296–361 |
| Nkp1 | 1–87, 1–97, ­1–155, 156–210 |
| Nkp2 | 1–115, 31–65, 116–139, 119–139 |

from multiple experiments

Ame1 was frequently proteolysed into many small fragments that were difficult to distinguish and detect in mass spectrometry. We only included abundant mass species in this table.
