# Supplementary material for: Molecular basis for inner kinetochore configuration through RWD domain–peptide interactions
Source: EMBO J. 2017 Oct 18;36(23):3458–82. doi: 10.15252/embj.201796636 (PMC5709738; doi:10.15252/embj.201796636)
Supplement: Supplementary file 5 — Table EV3 [file EMBJ-36-3458-s005.docx]

## Table EV3: Nkp1 and Nkp2 fragments identified in mass spectra from our limited proteolysis experiments with trypsin or elastase of *K. lactis* Nkp1-Nkp2, without subsequent chromatography

| Protein | Fragment (residue numbers *K. lactis* proteins) |
| --- | --- |
| Nkp1 | 1–71, 1–145, 1–149, 1–150, 1–152, 1–155, 72–97, 134–155, 153–210, 156–210 |
| Nkp2 | 1–89, 1–112, 1–115, 1–117, 116–139, 119–139 |

from multiple experiments
