# Supplementary material for: Molecular basis for inner kinetochore configuration through RWD domain–peptide interactions
Source: EMBO J. 2017 Oct 18;36(23):3458–82. doi: 10.15252/embj.201796636 (PMC5709738; doi:10.15252/embj.201796636)
Supplement: Supplementary file 6 — Table EV4 [file EMBJ-36-3458-s006.docx]

## ****Table** **EV****4: Statistics of X-ray diffraction data-collection, coordinate refinement, and final structure model of Ctf19-Mcm21-Okp1

| X-ray diffraction data collection and integration |  |
| --- | --- |
| Synchrotron X-ray source | APS 24-ID-E |
| Wavelength (Å) | 0.97918 |
| Space group | P22_1_2_1_ |
| Unit cell parameters (Å) | a=93.34, b=105.48, c=122.98 |
| Resolution range in Å (highest resolution shell) | 122.98–2.10 (2.15–2.10) |
| Number of unique reflections (multiplicity) | 71441 (5.3) |
| Completeness in % (highest resolution shell) | 99.9 (99.9) |
| Mean <I>/σ<I> (highest resolution shell) | 10.1 (0.5) |
| *CC_1/2_*^1^ (highest resolution shell) | 0.998 (0.184) |
| *R_meas_*^2^ (highest resolution shell) | 0.124 (4.247) |
| *R_sym_*^2^ (highest resolution shell) | 0.099 (3.407) |
| Wilson B factor (Å^2^) | 45 |
| Model-coordinate refinement |  |
| Number of reflections in working set/test set | 134132/3247 |
| *R_cryst_*^3^ overall (****2.13–2.10**** Å) | 0.2217 (0.4758) |
| *R_free_*^4^ overall (2.13–2.10 Å) | 0.2483 (0.4395) |
| *CC**^5^ for 2.13–2.10 Å shell | 0.555 |
| *CC_work_*^6^ for 2.13–2.10 Å shell | 0.408 |
| *CC_free_*^6^ for 2.13–2.10 Å shell | 0.378 |
| Average refined B factor^7^ (Å^2^) | 49.4 |
| No. of non-hydrogen atoms  Protein  Water  Ligand | 6126  338  1 |
| Final model analysis |  |
| Estimated coordinate error^8^ (Å) | 0.42 |
| R.m.s.d. bond lengths^9^ (Å) | 0.002 |
| R.m.s.d. bond angles^9^ (˚) | 0.456 |
| No. of residues in Ramachandran plot^10^ in  allowed region  generously allowed region  disallowed region | 706  25  1 |
| Molprobity score^10^ | 1.10 (100^th^ percentile from n=11758, 2.100 Å ± 0.25 Å) |
| Molprobity clash score^10^ | 1.38 (100^th^ percentile from n=576, 2.100 Å ± 0.25 Å) |
| Protein Data Bank (PDB) accession code | **5MU3** |

1) *CC_1/2_* is the Pearson correlation-coefficient between the average intensities of two random half data sets;

2) ;; with *I_h_* the intensity of reflection index *h*; and and *n_h_* multiplicity;

3) ; with *F_obs_* and *F_calc_* observed and calculated structure factors respectively; and *h* reflection indices;

4) *R_free_*: cross-validation of *R_crys_*_t_;

5)

6) *CC_work_* and *CC_free_* are standard and cross-validated correlations of the experimental intensities with the intensities calculated from our final refined model coordinates;

7) Refined *B* factor from phenix.refine;

8) Estimated coordinate error based on the *R_free_* value as calculated by phenix.refine.

9) Root mean square deviation (r.m.s.d.) from ideal values as calculated with phenix.refine;

10) Calculated with MolProbity. A score in the 100th percentile range is the best possible among crystal structures refined at comparable resolution. A score in the 0th percentile is the worst.
