# Supplementary material for: Molecular basis for inner kinetochore configuration through RWD domain–peptide interactions
Source: EMBO J. 2017 Oct 18;36(23):3458–82. doi: 10.15252/embj.201796636 (PMC5709738; doi:10.15252/embj.201796636)
Supplement: Supplementary file 9 — Movie EV1 [file EMBJ-36-3458-s009.zip › schmitz_movleg.docx]

Movie of crystal structure of Ctf19-Mcm21 bound with Okp1 binding segment, rotating.

Ctf19-Mcm21 D-RWD module is shown in transparent surface representation (Ctf19:blue; Mcm21:brown) with secondary structure cartoon underneath. Okp1 binding segment is shown in turquois colour in secondary structure cartoon representation.
