# Supplementary material for: Molecular basis for inner kinetochore configuration through RWD domain–peptide interactions
Source: EMBO J. 2017 Oct 18;36(23):3458–82. doi: 10.15252/embj.201796636 (PMC5709738; doi:10.15252/embj.201796636)
Supplement: Supplementary file 10 — Source Data for Appendix [file EMBJ-36-3458-s015.zip › emboj-2017_96636R-source_data_for_expanded_view_and_appendix/legend-to-files_README.docx]

## Legend to figure source data for Appendix supplementary figures

Folder-name: content

**SourceData_for_Appendix_FigS3AB:** deuterium exchange plots for Ctf19 or Mcm21 in COMA or COMA-Nkp1-Nkp2

**SourceData_for_Appendix_Fig5E:** deuterium exchange plots for Nkp1 in Nkp1 alone or in Nkp1-Nkp2

**SourceData_for_Appendix_FigS6AB:** deuterium exchange plots for Okp1 or Ame1 in Ame1-Okp1 or COMA
